# Genome-Wide Identification, Phylogenetic and Expression Pattern Analysis of GATA Family Genes in Cucumber (*Cucumis sativus* L.)

**DOI:** 10.3390/plants10081626

**Published:** 2021-08-07

**Authors:** Kaijing Zhang, Li Jia, Dekun Yang, Yuchao Hu, Martin Kagiki Njogu, Panqiao Wang, Xiaomin Lu, Congsheng Yan

**Affiliations:** 1 College of Agriculture, Anhui Science and Technology University, Fengyang 233100, China; zhangkj@ahstu.edu.cn (K.Z.); dekun_yang1@163.com (D.Y.); yuchao_hu1@163.com (Y.H.); luxiaomin88@163.com (X.L.); 2 Key Laboratory of Genetic Improvement and Ecophysiology of Horticultural Crop, Institute of Horticulture, Anhui Academy of Agricultural Sciences, Hefei 230001, China; jiali820@aaas.org.cn; 3 Department of Plant Science, Chuka University, Chuka P.O. Box 109-60400, Kenya; martnnjogu@gmail.com; 4 College of Horticulture, Henan Agricultural University, Zhengzhou 450002, China; panqiaowang@henau.edu.cn

**Keywords:** cucumber, GATA, gene family, expression pattern, stress response

## Abstract

GATA transcription factors are a class of transcriptional regulatory proteins that contain a characteristic type-IV zinc finger DNA-binding domain, which play important roles in plant growth and development. The GATA gene family has been characterized in various plant species. However, GATA family genes have not been identified in cucumber. In this study, 26 GATA family genes were identified in cucumber genome, whose physicochemical characteristics, chromosomal distributions, phylogenetic tree, gene structures conserved motifs, *cis*-regulatory elements in promoters, homologous gene pairs, downstream target genes were analyzed. Tissue expression profiles of cucumber GATA family genes exhibited that 17 GATA genes showed constitutive expression, and some GATA genes showed tissue-specific expression patterns. RNA-seq analysis of green and virescent leaves revealed that seven GATA genes might be involved in the chloroplast development and chlorophyll biosynthesis. Importantly, expression patterns analysis of GATA genes in response to abiotic and biotic stresses indicated that some GATA genes respond to either abiotic stress or biotic stress, some GATA genes such as Csa2G162660, Csa3G017200, Csa3G165640, Csa4G646060, Csa5G622830 and Csa6G312540 were simultaneously functional in resistance to abiotic and biotic stresses. Overall, this study will provide useful information for further analysis of the biological functions of GATA factors in cucumber.

## 1. Introduction

GATA transcription factors, a group of transcriptional regulatory proteins, are encoded by small multigene families. GATA protein sequences contain either one or two highly conserved type-IV zinc-finger motifs (C-X_2_-C-X_17–20_-C-X_2_-C) and a DNA-binding domain recognized as the DNA consensus sequence (A/T)GATA(A/G) [1]. The GATA family is widely distributed in eukaryotic organisms including animals, plants and fungi. In animals, GATA factors typically contain two conserved C-X_2_-C-X_17_-C-X_2_-C zinc-finger domains, but only the C-terminal finger is involved in DNA binding, and they have been shown to play critical roles in the processes of development, differentiation and cell proliferation [2]. In plants, the majority of GATA factors contain only a single C-X_2_-C-X_18_-C-X_2_-C or C-X_2_- C-X_20_-C-X_2_-C zinc finger domain, and several GATA factors encode two zinc finger domains. Plant GATA factors play important roles in plant growth and development, biotic and abiotic stresses, secondary metabolism and other biological processes [3]. The fungal GATA factors generally contain a C-X_2_-C-X_17_-C-X_2_-C or C-X_2_-C-X_18_-C-X_2_-C domain, and they have been shown to be involved in the global regulation of nitrogen metabolism, light-regulated photomorphogenesis, circadian regulation and mating-type switching [4,5].

The first plant GATA gene (*NTL1*) was cloned from *Nicotiana tabacum* [6]. The GATA family was subsequently identified in a number of plants, such as *Oryza sativa* [3], *Arabidopsis thaliana* [3,7], *Glycine max* [8], *Malus domestica* [9], *Vitis vinifera* [10], *Solanum lycopersicum* [11], *Gossypium raimondii*, *Gossypium arboreum*, *Gossypium hirsutum* [12], *Brassica napus* [13], *Brachypodium distachyon* [14], *Capsicum annuum* [15] and so on. In higher plants, GATA genes are involved in various biological processes. For instance, the *Arabidopsis* GATA2 (*At2g45050*) is a key light-signaling transcription factor that mediates photomorphogenesis [16]. GATA factor, Nitrate-inducible, Carbon metabolism-involved (*GNC*) and Cytokinin-responsive GATA1/GNC-Like (*CGA1*/*GNL*) serve important functions in chlorophyll synthesis and potentially regulate carbon and nitrogen metabolism [17]. It was also found that GATA transcription factor *PdGNC* regulates chloroplast ultrastructure and photosynthesis in poplar [18]. *ZIM* (*At4g24470*) is a GATA transcription factor involved in inflorescence and flower development [19]. The GATA transcription factor *HANABA TARANU* is required in early embryo development of *Arabidopsis* [20]. GATA factors have also been implicated in the regulation of nitrogen assimilation in plants. GATA motifs have been identified in the regulatory regions of many genes involved in nitrate assimilation such as nitrate reductase (*NIA*), nitrite reductase (*NiR*) and glutamine synthetase [21,22]. Furthermore, GATA factors are responsive to hormone signals, such as auxin and gibberellin signals, which regulate the downstream target genes *GNC* and *GNL* during plant growth and development; these signals also regulate brassinosteroid, which participates in the regulation of the GATA transcription factor *GATA2* during *Arabidopsis* photomorphogenesis [16,23]. These studies reported that the GATA transcription factors were involved in the regulation of photomorphogenesis, nitrogen metabolism, light-responsive development, chlorophyll biosynthesis, flowering transition and abiotic stress response. However, the biological functions of GATA transcription factor family members remain poorly understood.

Cucumber (*Cucumis sativus* L.), one of the most economically important vegetable crop species, is the first vegetable crop whose complete genome sequencing project has been finished [24]. Lots of gene families such as WRKY [25], MADS-box [26], NBS [27], bZIP [28], LEA [29], CLE [30] and so on have been reported in cucumber. However, the GATA transcription factors family has not been identified in cucumber. In addition, the functional analysis of GATA transcription factors mainly focused on the abiotic stress such as low nitrogen [8], light [9], cold, drought, salt [11,13] and phytohormones [10,14], but lack of expression patterns analysis of GATA genes in response to biotic stress. Therefore, in this study, we performed the systematic bioinformatics analysis of GATA transcription factors and analyzed the expression profiles of GATA family genes under the abiotic and biotic stresses in cucumber, which provides valuable information and candidate genes for cucumber resistance breeding.

## 2. Results

### 2.1. Genome-Wide Identification and Chromosomal Distribution of GATA Family Genes in Cucumber

A total of 26 GATA family members were identified from cucumber genome using HMMER 3.0 software. The physical and chemical properties of 26 cucumber GATA genes and encoded proteins, including coding sequence (CDS) sizes, number of amino acids, molecular weights, protein isoelectric points (pI), instability indexes, aliphatic indexes, grand average of hydropathicity (GRAVY) values, and genomic locations, were analyzed as shown in Table 1. The CDS size of 26 cucumber GATA genes ranged from 420 bp (Csa6G312540) to 1620 bp (Csa3G017200), with the number of amino acids of GATA proteins accordingly ranging from 139 to 539 aa. The molecular weights of 26 GATA proteins ranged from approximately 15.13 to 59.97 kD. The aliphatic indexes of 26 GATA proteins ranged from 32.48 (Csa6G502700) to 77.40 (Csa7G405980). The pI of 26 GATA proteins varied from 4.86 (Csa2G370420) to 9.83 (Csa4G286370). The instability index was greater than 40 for each GATA protein except Csa4G286370 and Csa7G405980, which suggested that most GATA proteins were stable proteins except Csa4G286370 and Csa7G405980. The GRAVY values of all 26 GATA proteins were less than zero, indicating that these proteins were hydrophilic.

Based on the physical positions of GATA genes annotated in the cucumber_ChineseLong_v2 GFF file, the chromosomal locations of 26 GATA genes were marked on the physical map of cucumber. The 26 GATA genes were located on all the seven cucumber chromosomes with different densities (Supplementary Figure S1). Chromosome 3 contained the largest number of GATA genes with six GATA genes. Chromosome 5 contained the lowest number of GATA genes with only one GATA gene.

### 2.2. Phylogenetic Analysis and Sequence Alignment of GATA Proteins

**Figure 1:**
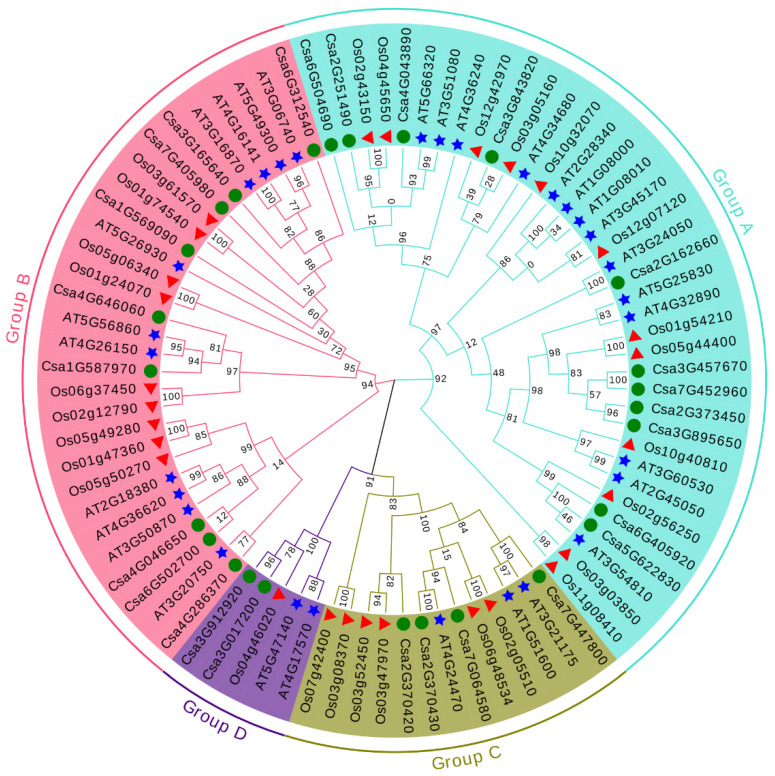
The phylogenetic tree of the total GATA proteins from cucumber, *Arabidopsis* and rice. Phylogenetic relationship of GATA proteins from cucumber (26), *Arabidopsis* (30) and rice (28) were performed with MEGA 7.0.26 using the maximum likelihood method with 1000 bootstrap replicates. The arcs with different colors represent four major groups of GATA proteins. GATA members of cucumber, *Arabidopsis*, and rice were represented by green circles, blue stars, and red triangles, respectively. The number represented the bootstrap replicates.

**Figure 2:**
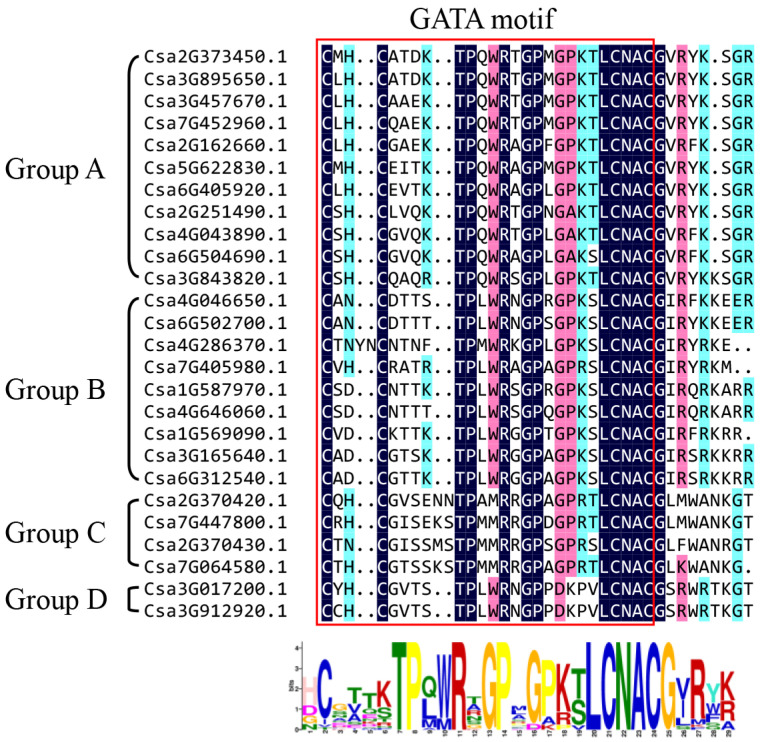
Alignment of conserved domain sequences from 26 GATA proteins in cucumber. GATA motif and amino acid sites were shown at the top, and sequence identities were shown at the bottom.

To analyze the phylogenetic relationship of the GATA genes among different species and classify the GATA genes identified in cucumber, a maximum likelihood phylogenetic tree was constructed based on the multiple sequences alignment of 26 cucumber GATA proteins, 30 *A. thaliana* GATA proteins and 28 rice GATA proteins (Figure 1). According to the classification of *Arabidopsis* and rice GATA proteins, cucumber GATA family proteins were divided into four groups (A, B, C and D). Among the four classified groups, group A had the largest number of cucumber GATA proteins (11 GATA proteins), accounting for 42.3% of the total cucumber GATA proteins. Group D had the least number of GATA proteins with only two members (8.0%), namely, Csa3G017200 and Csa3G912920. To further analyze the sequence features of the 26 cucumber GATA proteins, their conserved domain sequences were aligned. The multiple sequence alignment revealed that all GATA proteins contained the conserved domain C-X_2_-C-X_18–20_-C-X_2_-C with the exception of Csa4G286370 which possessed two extra amino acids to form C-X_4_-C-X_18_-C-X_2_-C (Figure 2). The characteristics of cucumber GATA domains in each group were generally consistent with previously studied GATA domains in *A. thaliana*. For example, all GATA members in group C had an insertion of two amino acids. The GATA motifs and conserved amino acid sites in different groups may contribute to the various functions of these GATA proteins.

### 2.3. Phylogenetic, Gene Structure and Conserved Motif Analysis of Cucumber GATA Proteins

**Figure 3:**
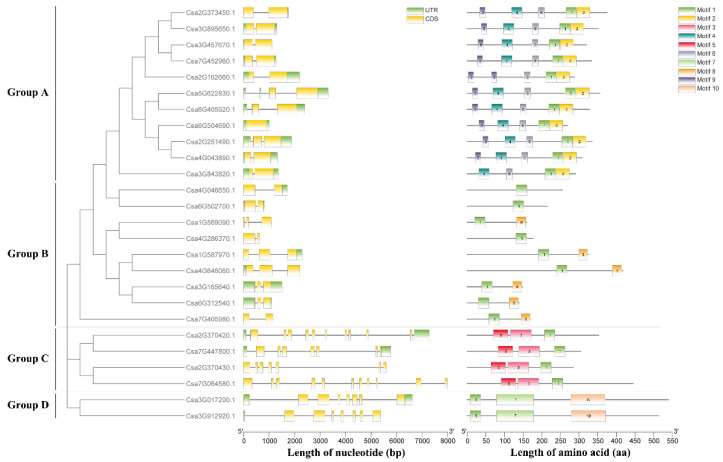
Phylogenetic relationship, gene structure, and conserved motif analysis of cucumber GATA genes. Left: Phylogenetic tree of 26 cucumber GATA proteins. The neighbor-joining phylogenetic tree was constructed using MEGA 7.0.26, with 1000 replicates. Middle: Exon-intron structures of cucumber GATA genes. Orange boxes represent exons, black lines represent introns, and the upstream/downstream regions of GATA genes are represented by green boxes. Right: Conserved motifs of cucumber GATA proteins. Ten conserved motifs are shown in different colored boxes, and the details of the motifs are provided in Table S1.

Analysis of the exon/intron organization of 26 cucumber GATA genes revealed that the numbers of exon in GATA genes varied from 1 (Csa6G504690) to 11 (Csa7G064580). Group A contained the lowest average number of exons per gene, 1.9, while group C had the highest, 8.8. Furthermore, the structural characteristics of GATA genes in the same group were similar but varied among different groups (Figure 3). For example, in group C, each GATA gene contained more than seven exons, while each GATA gene in group B comprised two or three exons. A total of 10 conserved motifs, designated as motifs 1–10, were identified in the 26 cucumber GATA proteins. The amino acid sequences of each conserved motif were shown in Supplementary Table S1. Most GATA proteins in the same group generally contained similar conserved motif compositions (Figure 3). For example, GATA proteins in group A had an average of five conserved motifs, including motif 1, which was annotated as the GATA zinc finger domain according to the Pfam database, and the other motifs 2, 4, 6, 9. In addition to motif 1, all GATA proteins in group C contained conserved motifs 3 and 5, representing CCT and TIFY domains, respectively. All GATA proteins in group D contained motif 7 and motif 10 (representing ASXH and RPN13_C domains, respectively). Taken together, the conserved motif compositions of GATA proteins in the same group were similar but varied among different groups.

### 2.4. Homologous Gene Pairs and Synteny Analysis

**Figure 4:**
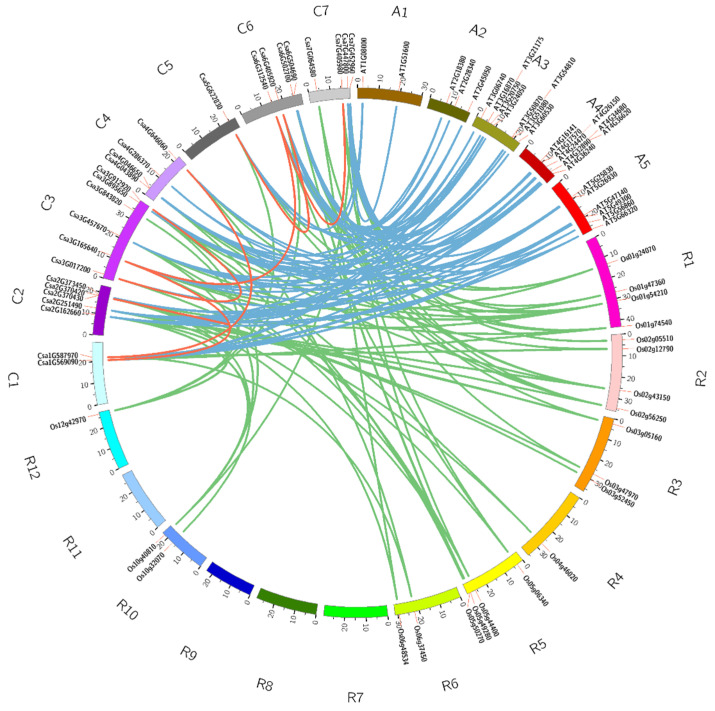
Syntenic relationships of GATA gene family in cucumber, *Arabidopsis* and rice. The red lines represent the segmentally duplicated GATA genes in cucumber. The blue lines represent the orthologous relationships of GATA genes between cucumber and *Arabidopsis*. The green lines represent the orthologous relationships of GATA genes between cucumber and rice.

Analysis of cucumber GATA gene duplication events identified seven pairs of putative paralogous genes including one tandem duplication (Csa2G370420/Csa2G370430) and six segmental duplications (Csa1G569090/Csa3G165640, Csa1G587970/Csa4G646060, Csa3G017200/Csa3G912920, Csa3G165640/Csa6G312540, Csa5G622830/Csa6G405920, Csa6G312540/Csa7G405980), which suggest that segmental duplication played a crucial role in the expansion of the GATA gene family in cucumber. The orthologous GATA gene pairs among cucumber, *A. thaliana* and rice were also investigated in this study. The results indicated that 22 cucumber GATA genes and 28 *A. thaliana* GATA genes were orthologous gene pairs, which resulted in the 71 syntenic relationships across these two species (Supplementary Table S2). 25 cucumber GATA genes and 21 rice GATA genes were orthologous gene pairs with 59 syntenic relationships (Figure 4 and Supplementary Table S3). Only Csa4G286370 gene in cucumber did not form the syntenic relationship with neither *A. thaliana* nor rice, which means that Csa4G286370 was conservative in cucumber GATA gene family.

### 2.5. Cis-Acting Regulatory Elements in the Promoters of Cucumber GATA Genes

**Figure 5:**
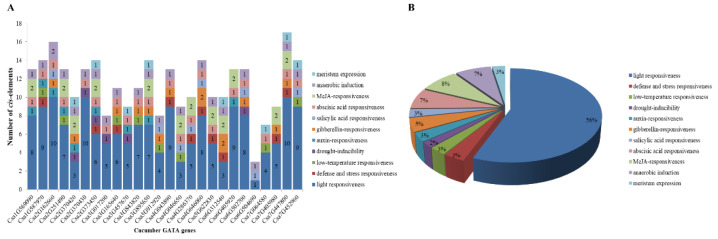
*Cis*-regulatory elements in the promoters of 26 cucumber GATA genes. (**A**) The number of various *cis*-regulatory elements in the promoters of each cucumber GATA gene. (**B**) The relative proportions of different *cis*-regulatory elements in the promoters of cucumber GATA genes are indicated by the pie chart. *Cis*-regulatory elements sharing identical or similar functions are represented by the same color.

*Cis*-acting regulatory elements analysis identified 11 main types of *cis*-regulatory elements in the promoter sequences of cucumber GATA genes. The light-responsiveness *cis*-regulatory elements accounts for the largest proportion (up to 56%) in the total across the promoters of 26 GATA genes, which contains different kinds of *cis*-regulatory elements such as ACE, G-box, and MRE. Additionally, the *cis*-regulatory elements associated with hormone response (including auxin, salicylic acid, gibberellins, abscisic acid, and MeJA), stress response (including drought, low temperature, defense and stress); meristem expression, anaerobic induction were also identified in promoter sequences of the cucumber GATA genes (Figure 5).

### 2.6. The Downstream Target Genes Analysis of Cucumber GATA Genes

**Figure 6:**
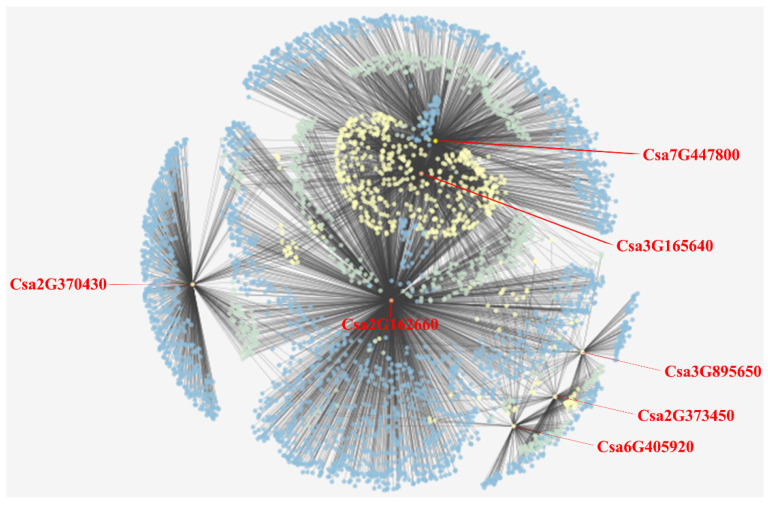
The regulatory interactions network between cucumber GATA genes and their target genes. The genes marked in red are the cucumber GATA genes. The detailed downstream target genes of cucumber GATA genes are shown in the Table S4.

Through the website of Plant Transcriptional Regulatory Map, the target genes analyses of cucumber GATA genes were conducted. The target genes of cucumber GATA family genes were shown in Supplementary Table S4. Only seven cucumber GATA genes including Csa2G162660, Csa2G370430, Csa2G373450, Csa3G165640, Csa3G895650, Csa6G405920 and Csa7G447800 were found to regulate the target genes; no target gene was found for other GATA genes (Figure 6 and Table S4). Among the seven cucumber GATA genes, the gene Csa2G162660 has the largest number of target genes (1910), and the gene Csa3G895650 has the lowest number of target genes (99). These results will be a benefit to the research of transcriptional regulatory network of GATA genes.

### 2.7. Tissue Expression Profiles Analysis of Cucumber GATA Genes

**Figure 7:**
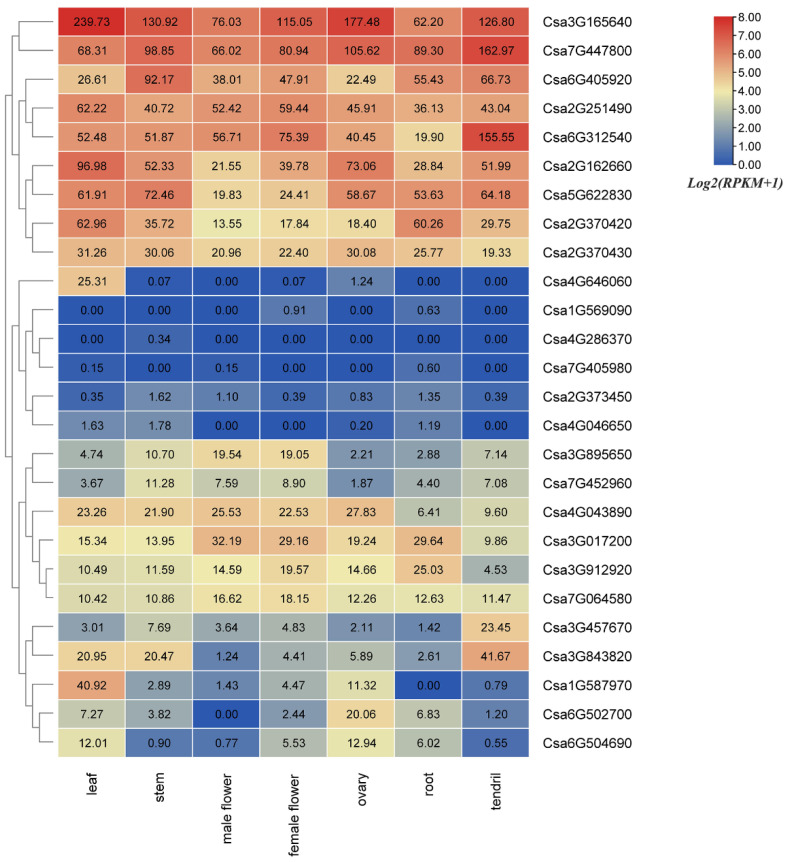
Tissue-specific expression of GATA genes in cucumber. The transcriptional levels of GATA genes in seven tissues (leaf, stem, male flower, female flower, ovary, root, and tendril) of cucumber 9930 were investigated based on a public transcriptome data. The heatmap was constructed using the TBtools software, and the RPKM (reads per kilobase per million mapped reads) values of GATA genes were transformed by *log2(RPKM+1)*. The data in the boxes indicate original RPKM values. The red and blue colors represent the higher and lower relative expression levels, respectively.

The expression patterns of all 26 cucumber GATA genes were investigated based on public transcriptomic data of different tissues of cucumber, including leaf, stem, male flower, female flower, ovary, root and tendril. Among the 26 GATA genes, 19 GATA genes were expressed in all detected samples (RPKM > 0), and 17 genes showed constitutive expression (RPKM > 1 in all samples). Overall, 34.6% (9/26) of GATA genes were highly expressed in different tissues of cucumber. Of GATA genes, 19.2% (5/26) were low or not expressed in any tissues. Additionally, 23.1% (6/26) of GATA genes were middle expressed in different tissues of cucumber. The other cucumber GATA genes were specially expressed in some tissues, such as Csa1G587970 and Csa4G646060 were highly expressed in leaf, Csa3G457670 and Csa3G843820 were highly expressed in tendril, Csa6G502700 was highly expressed in ovary, Csa6G504690 was highly expressed in leaf and ovary (Figure 7). These results revealed that the expression patterns of cucumber GATA genes were diverse in different tissues.

### 2.8. Expression Profiles Analysis of Cucumber GATA Genes during Chlorophyll Biosynthesis

**Figure 8:**
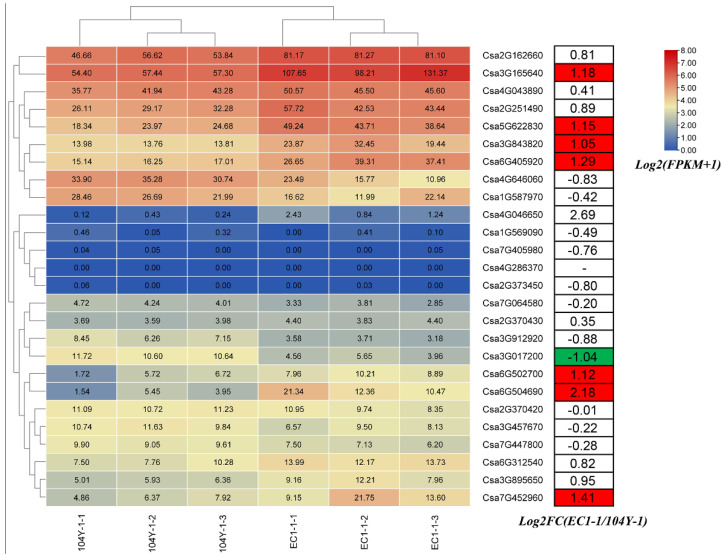
Expression profiles of cucumber GATA genes between green and virescent leaves. The fragments per kilobase of transcript per million fragments (FPKM) values of GATA genes were transformed by *log2(FPKM+1)*. The data in the boxes indicated original FPKM values. The red and blue colors represent the higher and lower relative expression levels, respectively. In the right table, differentially expressed genes (DEGs) are highlighted by red (up-regulation) and green (down-regulation). FC represent fold-change. 104Y-1 represent the first true leaf of virescent plant 104Y, 104Y-1-1, 104Y-1-2 and 104Y-1-3 were three biological replications of virescent leaves. EC1-1 represent the first true leaf of green plant EC1, EC1-1-1, EC1-1-2 and EC1-1-3 were three biological replications of green leaves.

To explore the potential functions of cucumber GATA genes in chloroplast development and chlorophyll biosynthesis, RNA-seq analysis of green and virescent true leaves were conducted. As compared with the virescent leaf, most GATA genes were up-regulated in the green leaf. As shown in Figure 8, eight cucumber GATA genes were differentially expressed between green and virescent leaves. Among them, seven cucumber GATA genes including Csa3G165640, Csa5G622830, Csa3G843820, Csa6G405920, Csa6G502700, Csa6G504690 and Csa7G452960 were significantly induced in the green leaf compared with virescent leaf, only one cucumber GATA gene Csa3G017200 was significantly down-regulated in the green leaf compared with virescent leaf. Notably, although the Log_2_FC(EC_1/104Y_1) value of Csa4G046650 was 2.69, the FPKM value in green and virescent leaves were all lower than five, which would be filtered. Thus, the above results revealed that seven cucumber GATA family genes might be involved in the chloroplast development and chlorophyll biosynthesis.

### 2.9. Expression Profiles Analysis of Cucumber GATA Genes under Abiotic Stresses

**Figure 9:**
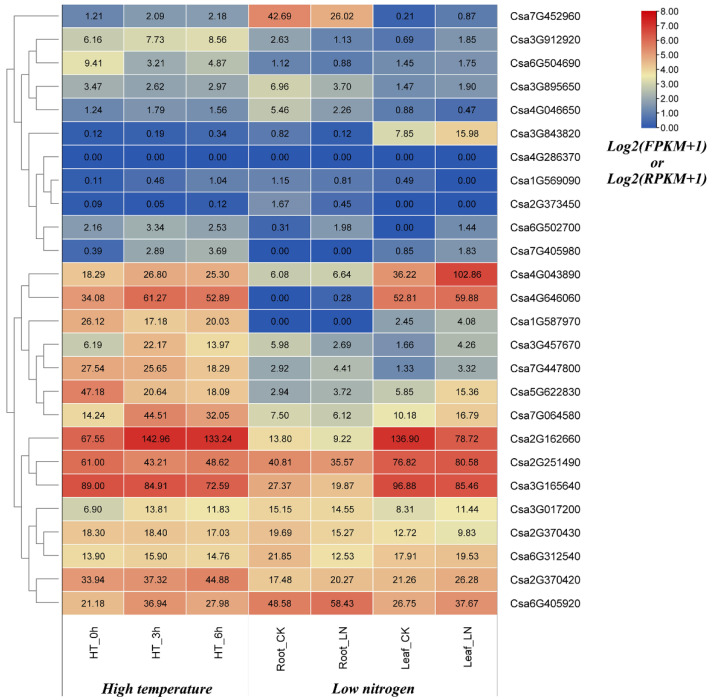
Expression profiles of cucumber GATA genes in response to various abiotic stress treatments including high temperature, low nitrogen and GA. HT = high temperature; HT_0h = heat treatment for 0 h (hours); HT_3h = heat treatment for 3 h; HT_6h = heat treatment for 6 h. CK means control plant; LN means low nitrogen. The FPKM or RFPKM values of GATA genes were transformed by *log2(FPKM+1)* and *log2(RPKM+1)*. The data in the boxes indicate original FPKM or RPKM values. The red and blue colors represent the higher and lower relative expression levels, respectively.

To understand the expression profiles of cucumber GATA genes under abiotic stresses, the available transcriptomic data were used to analysis the expression levels of cucumber GATA genes in response to the treatments of high temperature and low nitrogen. Under high temperature treatment, six GATA genes were significantly induced/repressed by high temperatures. Among them, the expression levels of Csa2G162660, Csa3G017200, Csa7G064580, Csa4G646060 and Csa3G457670 were significantly up-expressed. The expression levels of these five GATA genes in response to high temperature for 3 h (hours) was higher than that for 6 h, which revealed that these five GATA genes responded quickly to high temperatures. The expression level of Csa5G622830 was significantly down-expressed in resistance to high temperature, which suggested that this GATA gene was repressed by high temperatures. Under the low nitrogen stress, Csa6G312540 and Csa7G452960 were down-regulated in root, Csa2G162660 was down-regulated expressed in leaf, and Csa4G043890 was up-regulated in leaf. Thus, these four cucumber GATA genes were associated with the response to low nitrogen (Figure 9). 

### 2.10. Expression Profiles Analysis of Cucumber GATA Genes under Biotic Stresses

**Figure 10:**
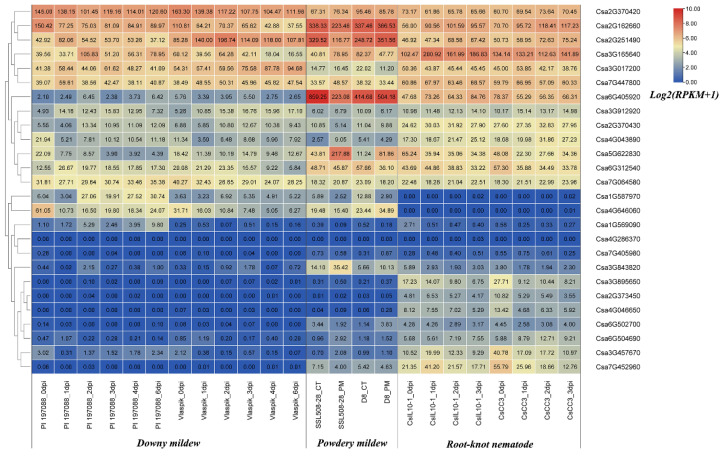
Expression profiles of cucumber GATA genes in response to various biotic stress treatments including downy mildew, powdery mildew and root-knot nematode. PI 197088 is the downy mildew-resistant cucumber plant; Vlaspik is the downy mildew-susceptible cucumber plant; dpi means the days post inoculation. SSL508-28 is the powdery mildew-resistant cucumber plant; D8 is the powdery mildew-susceptible cucumber plant; CT is the control plant; PM is the abbreviation of powdery mildew. CsIL10_1 is the root-knot nematode resistant cucumber plant, CsCC3 is the root-knot nematode susceptible cucumber plant. The RPKM values of GATA genes were transformed by *log2(RPKM+1)*. The data in the boxes indicated original RPKM values. The red and blue colors represented the higher and lower relative expression levels, respectively.

Previous studies only performed the functional analysis of plant GATA genes under abiotic stresses such as cold, salt and drought. In this study, the expression patterns of cucumber GATA genes under biotic stresses including downy mildew, powdery mildew and root-knot nematode were analyzed with the big data of cucumber transcriptome sequencing (Figure 10). After the treatment with downy mildew inoculation, the expression levels of Csa2G162660, Csa4G646060 and Csa5G622830 in both resistant and susceptible cucumber lines were all down-regulated. With the extension of inoculation time in the resistant cucumber line, the expression levels of Csa2G251490 and Csa3G017200 were initially increased and then decreased to the similar expression level as the control plant. While in the susceptible cucumber line, the expression levels of Csa2G251490 and Csa3G017200 were up-regulated after inoculation and then down-regulated to the expression levels that were higher than the expression levels of mock plant. Csa6G312540 was up-regulated after inoculation in the resistant cucumber line and then decreased to the expression level that was higher than the expression level of mock plant; however, Csa6G312540 was up-regulated after inoculation in the susceptible cucumber line and then down-regulated to the expression level that was lower than the expression level of mock plant. Thus, these six cucumber GATA genes were associated with downy mildew resistance in cucumber.

Under the stress of powdery mildew inoculation, the expression levels of Csa5G622830 were up-regulated in the resistant and susceptible cucumber lines. The expression levels of Csa2G162660, Csa2G251490 and Csa6G405920 were down-regulated in the resistance cucumber line and up-regulated in the susceptible cucumber line. The expression level of Csa3G165640 was up-regulated in the resistance cucumber line and down-regulated in the susceptible cucumber line. The expression levels of Csa3G017200 and Csa6G312540 did not change in the resistant cucumber line; however, they were down-regulated in the susceptible cucumber line. These results indicate that the above seven cucumber GATA genes were related to the powdery mildew resistance in cucumber.

After the treatment of root-knot nematode infection, the expression levels of Csa2G162660 and Csa3G165640 were up-regulated in the resistant and susceptible cucumber plants, and the expression levels in the resistant cucumber were higher than those in the susceptible cucumber. The expression levels of Csa5G622830 were down-regulated in the resistant and susceptible cucumber lines. The expression level of Csa6G405920 was up-regulated in the resistant cucumber and down-regulated in the susceptible cucumber. The results showed that these four cucumber GATA genes responded to the root-knot nematode infection.

## 3. Discussion

Plant transcription factors, such as WRKY [31], MYB [32], bHLH [33], and zinc-finger [34], play a key role in governing gene regulation that mediates diverse biological processes in plant developmental processes, stress responses, and hormone signaling pathways [35]. GATA proteins are defined as GATA transcription factors due to their specific binding to the consensus sequence (A/T) GATA (A/G), which play important roles in plant growth and development [3]. The GATA gene family has been identified in various plant species, such as *Oryza sativa* [3], *Arabidopsis thaliana* [3,7], *Glycine max* [8], *Malus domestica* [9], *Vitis vinifera* [10], *Solanum lycopersicum* [11], *Gossypium raimondii*, *Gossypium arboreum*, *Gossypium hirsutum* [12], *Brassica napus* [13], *Brachypodium distachyon* [14], *Capsicum annuum* [15] and so on. Cucumber is the first vegetable crop whose whole-genome sequencing has been finished; however, genome-wide identification of GATA gene family in cucumber has not been conducted yet. Therefore, genome-wide characterization and expression analysis of GATA gene family in cucumber will help us understand further GATA gene functions.

In this study, it is the first time to identify and characterize GATA gene family in cucumber using bioinformatics methods. A total of 26 GATA genes were identified and classified into four subfamilies (groups A to D) in cucumber (Figure 1; Figure 3). Consistent with *A. thaliana* and rice, group A harbored the largest number of GATA genes (Figure 1). The results of phylogenetic tree analysis were, to some extent, consistent with the results of synteny analysis, which means that these GATA homologous gene pairs were more closely related to each other ( Figure 1; Figure 4). The analysis of exon/intron structure and conserved motifs revealed that the GATA genes in each subfamily have special characteristics. A comparison of gene structures indicated that the number of exons/introns and motifs varies between subfamilies, but is similar within each subfamily (Figure 3).

Gene duplication events including tandem, segment and transposition duplications are crucial in genomic rearrangement, which often result in expansion of gene family [36]. The GATA genes in cucumber only contained one tandem duplication and six segmental duplications (Figure 4), indicating that the GATA genes did not undergo the large-scale gene expansion. Most GATA genes in cucumber may involve an early divergence time or be obtained from gene transposition, which is consistent with previous studies demonstrating the absence of recent whole-genome duplication resulting the presence of few tandem in cucumber [24].

Along with the rapid development of high-throughput sequencing technologies, numerous omics studies, especially genome and transcriptome analysis, have been widely conducted. The time of big data has been coming. The big data of cucumber transcriptome sequencing have been validated with the qRT-PCR analysis and peer-reviewed, which could be considered as reliable data. Therefore, the effective utilization of these big data regarding cucumber transcriptome sequencing can not only reduce the research cost, but also facilitate the deep mining of the data of each transcriptome sequence [37,38,39,40]. In this study, the expression profiles of GATA genes were performed based on eight types of public cucumber transcriptome data. The genes Csa4G286370 and Csa7G405980 were not expressed in any tissues (Figure 7), which reveals that the two genes might be non-functional genes or occur transcriptional gene silencing and post-transcriptional gene silencing [41,42]. Some genes were highly expressed in different tissues and some genes were expressed in specific tissues, which showed the functional difference in GATA genes in cucumber. The expression profiles analysis of cucumber GATA genes in green and virescent leaves shows that seven GATA genes might be involved in the chloroplast development and chlorophyll biosynthesis (Figure 8), which is consistent with the functions of *Arabidopsis* homologous GATA genes *GNC* and *CGA1* [17]. In the soybean and poplar, the GATA genes *GmGATA58* and *PdGATA19* were also demonstrated to be involved in regulating chlorophyll biosynthesis [18,43].

To comment on the role of cucumber GATA genes in abiotic stresses, we analyzed the expression patterns of these GATA genes in response to high temperature and low nitrogen treatments based on the published cucumber transcriptome sequencing data (Figure 9). In our study, four cucumber GATA genes (Csa6G312540, Csa7G452960, Csa2G162660 and Csa4G043890) were associated with the response to low nitrogen. In a previous study, four soybean GATA genes (*GmGATA10/16/24/62*) also exhibited different expression levels in both leaves and roots compared with the control under the low nitrogen treatment [8]. Interestingly, several cucumber GATA genes were identified to respond to both kinds of abiotic stresses. For example, under the treatments of high temperature and low nitrogen, the GATA gene Csa2G162660 was simultaneously differentially expressed between the control and treated materials. However, some other GATA genes were only differentially expressed under one type of abiotic stresses, for example, Csa5G622830 was only down-regulated under high temperature treatment, the expression level did not change under low nitrogen treatment. The results revealed that some GATA genes play the general roles under several kinds of abiotic stresses, while some other GATA genes only play roles under one specific abiotic stress.

In addition to the abiotic stresses, the expression patterns of cucumber GATA genes were also observed under the biotic stresses (Figure 10). Expression patterns analysis showed that six, seven, and four cucumber GATA genes responded to downy mildew, powdery mildew, and root-knot nematode infections, respectively. Among them, two GATA genes Csa2G162660 and Csa5G622830 were all differentially expressed between control and treated materials after the infections of downy mildew, powdery mildew and root-knot nematode. Earlier, it had been reported that Csa5G622830 was the candidate gene for the downy and powdery mildew resistance in cucumber from our lab [44]. After the infections of downy mildew and powdery mildew, the expression levels of three GATA genes including Csa2G251490, Csa3G017200 and Csa6G312540 were simultaneously changed between control and treated materials. After the infections of powdery mildew and root-knot nematode, Csa6G312540 and Csa6G405920 were differentially expressed between control and treated materials. The cucumber GATA gene Csa4G646060 was only functional to downy mildew, while not resistant to powdery mildew and root-knot nematode. These results show that some GATA genes such as Csa2G162660 and Csa5G622830 were broad-spectrum resistance, while some GATA genes such as Csa4G646060 were specific resistance. In the previous study, it had been reported that plant GATA transcription were related with some diseases. For instance, 10 *Brachypodium distachyon* GATA genes responded to invasion of the fungal pathogen *Magnaporthe oryzae* [14]. grape *VdGATA2* enhanced the resistance to powdery mildew [45]. *DvGATA* was involved in defense to wheat powdery mildew [46]. *Arabidopsis* GATA23 is the essential gene for gall formation [47]. Wheat *TaGATA1* positively modulates host immune response to *Rhizoctonia cerealis* [48].

Additionally, some GATA genes not only play the roles in response to the biotic stress, but also in response to the abiotic stress; for example, Csa5G622830 were not only functioned after the treatments of downy mildew, powdery mildew and root-knot nematode infections, but also responded to high temperature treatment. Csa2G162660 and Csa6G312540 were not only functioned after the treatments of downy mildew, powdery mildew and root-knot nematode infections, but also responded to low nitrogen stress. The results showed that some GATA genes play important roles in response to abiotic and biotic stresses. In this study, in total, six cucumber GATA genes including Csa2G162660, Csa3G017200, Csa3G165640, Csa4G646060, Csa5G622830 and Csa6G312540 were simultaneously functional in resistance to abiotic and biotic stresses.

## 4. Materials and Methods

### 4.1. Identification and Chromosomal Distribution of GATA Genes in Cucumber

To identify all the members of GATA transcription factors in cucumber, the Hidden Markov Model (HMM) file corresponding to GATA zinc finger domain (PF00320) was downloaded from protein family (Pfam) database and used as a query to search all the putative GATA genes in the cucumber genome based on an expected value (E-value) cutoff of 1 × 10^−5^ in HMMER 3.0 [49]. Subsequently, each of all putative cucumber GATA genes was confirmed in the SMART database (http://smart.embl-heidelberg.de/ (accessed on 12 April 2021)) [50] and the NCBI Conserved Domain database (http://www.ncbi.nlm.nih.gov/Structure/cdd/wrpsb.cgi (accessed on 12 April 2021)) [51]. The protein sequences of confirmed cucumber GATA transcription factor family members were analyzed with Prosite ExPASy server (http://web.expasy.org/protparam/ (accessed on 13 April 2021)) to predict their physicochemical characteristics. The chromosomal position of each confirmed GATA gene was retrieved from the GFF3 file of ChineseLong_V2 and then visualized on the cucumber chromosomes with TBtools [52].

### 4.2. Phylogenetic Analysis of GATA Family Genes in Cucumber, Arabidopsis and Rice

Based on the studies of GATA family genes in *Arabidopsis* and rice [3,7], the GATA zinc finger domain sequences of 30 *Arabidopsis thaliana* GATA proteins and 28 rice GATA proteins were downloaded, respectively. Multiple alignments of GATA protein sequences of cucumber, *Arabidopsis* and rice were performed by Muscle in MEGA 7.0.26 [53] with default parameters. Phylogenetic trees were then constructed based on the alignments using the maximum likelihood method with 1000 bootstrap replicates. The parameters were Jones-Taylor-Thornton (JTT), gamma distributed (G) rates, and partial deletion. The trees were visualized and optimized via Evolview (http://www.evolgenius.info/evolview (accessed on 15 April 2021)).

### 4.3. Gene Structure, Conserved Motif, Promoter Sequence Analyses of Cucumber GATA Genes 

The locations of exons, introns and untranslated regions of each cucumber GATA gene were retrieved from GFF3 file of ChineseLong_V2. The conserved motifs in cucumber GATA proteins were determined with MEME server (http://memesuite.org/ (accessed on 15 April 2021)) [54] using the following parameters: maximum number of motifs, 10; minimum motif width, 6; and maximum motif width, 100. Exon/intron structures of cucumber GATA genes and conserved motifs of cucumber GATA proteins were visualized using the software TBtools [52]. Conserved domains sequences of cucumber GATA proteins were analyzed using DNAMAN software (http://en.bio-soft.net/format/DNAMAN.html (accessed on 15 April 2021)). The 1500 bp sequences upstream of the start codon of each cucumber GATA gene was extracted from the cucumber genome sequences and then submitted to the PlantCARE database (http://bioinformatics.psb.ugent.be/webtools/plantcare/html/ (accessed on 15 April 2021)) [55] for *cis*-regulatory elements prediction. The predicted *cis*-regulatory elements were classified according to their regulatory functions.

### 4.4. Detection of Homologous Gene Pairs and Synteny Analysis

The homologous gene pairs and syntenic relations of GATA family genes in cucumber were identified using Multiple Collinearity Scan toolkit (MCScanX) software [56] with default parameters. To predict the gene functions of cucumber GATA genes, the syntenic relationships of the orthologous GATA genes between cucumber and the model plants (*Arabidopsis* and rice) were examined. The syntenic relationships of GATA genes among cucumber, *Arabidopsis* and rice were explored using MCScanX software with the default parameters. The homologous genetic relationships of GATA genes among cucumber, *Arabidopsis* and rice were illustrated with Circos software [57].

### 4.5. Regulatory Interactions Analysis between GATA Genes and Their Target Genes

The target genes of each GATA gene were retrieved and counted from the total transcription regulatory networks of cucumber downloaded from Plant Transcriptional Regulatory Map (http://plantregmap.cbi.pku.edu.cn/download.php#networks (accessed on 17 April 2021)). The regulatory interactions network between cucumber GATA genes and their target genes was visualized with Cytoscape version 3.7.0 software (http://cytoscape.org/ (accessed on 17 April 2021)) [58].

### 4.6. Expression Profiles Analysis of Cucumber GATA Genes with Cucumber Transcriptome Sequencing Big Data

The expression data of cucumber GATA genes between the first true leaves of green and virescent plant were obtained from our previous study (PRJNA612596) [59]. The expression data of cucumber GATA genes in different tissue (PRJNA80169) [60] and different biotic stresses including downy mildew resistance (PRJNA285071) [61], powdery mildew resistance (PRJNA321023) [62], root-knot nematode resistance (PRJNA419665) [63] were all obtained from Cucurbit Genomics Database (CuGenDB) (http://cucurbitgenomics.org/rnaseq/home (accessed on 20 April 2021)). The expression data of cucumber GATA genes under high temperature (GSM4565536) [40] and low nitrogen (GSE46678) [64] treatments were downloaded from the NCBI’s GEO (Gene Expression Omnibus) database (https://www.ncbi.nlm.nih.gov/geo/ (accessed on 20 April 2021)). The heatmap of each GATA gene in above experiments were visualized with the TBtools software [52].

## 5. Conclusions

In this study, it is the first time the GATA gene family in cucumber have been identified and characterized. A total of 26 cucumber GATA genes were obtained and classified into subfamilies A–D after systematic investigations. An overview of the cucumber GATA factor gene family was revealed through the comprehensive investigation of their physicochemical characteristics, chromosomal location, phylogenetic tree, gene structure, conserved motif, *cis*-regulatory elements in the promoters, homologous gene pairs, synteny, and target genes. Tandem and segmental duplications contributed to the expansion of the GATA gene family, and segmental duplication tended to play the predominant role. A comparative analysis of the GATA factor gene family across cucumber, *Arabidopsis*, and rice helped us facilitate further gene function analysis of cucumber GATA genes. The expression patterns of the cucumber GATA genes in different cucumber tissues, between green and virescent leaves, and in response to various stresses then showed that these genes may play important roles in cucumber growth and development. Our results also provide useful information by identifying candidate tissue-specific, chlorophyll biosynthesis, abiotic and biotic stresses responsive cucumber GATA genes. This study not only provided a scientific foundation for the comprehensive understanding of the cucumber GATA gene family, but was also helpful for screening more candidate genes and breeding new varieties of cucumber with a high yield and stress resistance.

## Figures and Tables

**Table 1:** Detailed information of 26 predicted GATA proteins in cucumber. CDS, the coding sequence of a gene; pI, protein isoelectric point.

Gene Name	CDS Size (bp)	Number of Amino Acids (aa)	Molecular Weight (kD)	pI	Instability Index	Aliphatic Index	Grand Average of Hydropathicity	Genomic Location
Csa1G569090	480	159	17.40	9.41	43.36	63.14	−0.597	Chr1:20743377-20744463
Csa1G587970	978	325	35.68	8.71	57.34	64.25	−0.664	Chr1:22118620-22120914
Csa2G162660	864	287	31.02	6.53	67.35	62.2	−0.570	Chr2:9330657-9332857
Csa2G251490	1005	334	36.47	5.86	63.14	60.42	−0.580	Chr2:12381097-12382974
Csa2G370420	1059	352	38.58	4.86	44.39	62.84	−0.706	Chr2:18236800-18244086
Csa2G370430	855	284	30.70	6.32	43.10	66.58	−0.630	Chr2:18245818-18251412
Csa2G373450	1125	374	40.47	5.25	60.87	62.17	−0.647	Chr2:18666831-18668584
Csa3G017200	1620	539	59.97	6.49	56.47	68.72	−0.632	Chr3:1732854-1739477
Csa3G165640	447	148	16.11	9.71	66.68	63.92	−0.785	Chr3:10937552-10939059
Csa3G457670	960	319	34.84	5.67	55.15	52.07	−0.579	Chr3:20795136-20796243
Csa3G843820	873	290	32.23	9.38	68.64	65.24	−0.662	Chr3:34100073-34101438
Csa3G895650	1056	351	39.08	5.56	42.17	53.39	−0.813	Chr3:38551528-38552834
Csa3G912920	1542	513	57.19	6.19	58.56	68.58	−0.659	Chr3:39577751-39583126
Csa4G043890	924	307	34.24	5.48	75.12	63.49	−0.678	Chr4:3394536-3395860
Csa4G046650	768	255	27.06	9.21	49.09	50.24	−0.656	Chr4:3624324-3626040
Csa4G286370	531	176	19.86	9.83	37.02	47.73	−1.150	Chr4:11065609-11066229
Csa4G646060	1254	417	46.03	7.67	48.48	53.12	−0.953	Chr4:21924862-21927071
Csa5G622830	1068	355	38.46	5.85	57.10	66.51	−0.543	Chr5:24735193-24738505
Csa6G312540	420	139	15.13	9.76	60.79	52.01	−0.950	Chr6:14872370-14873463
Csa6G405920	984	327	36.03	5.39	58.06	72.14	−0.570	Chr6:18352364-18354766
Csa6G502700	645	214	24.17	7.6	56.34	32.48	−1.028	Chr6:25337427-25338242
Csa6G504690	807	268	30.48	7.22	55.43	53.84	−0.962	Chr6:25624800-25625808
Csa7G064580	1335	444	48.13	6.15	46.66	75.97	−0.405	Chr7:3845510-3853491
Csa7G405980	510	169	18.21	9.08	38.63	77.4	−0.275	Chr7:15586933-15588082
Csa7G447800	912	303	33.95	6.39	44.74	64.65	−0.871	Chr7:18027186-18032948
Csa7G452960	1002	333	36.32	6.26	49.71	59.19	−0.608	Chr7:19135685-19136959

## Supplementary Materials

The following are available online at https://www.mdpi.com/article/10.3390/plants10081626/s1, Figure S1: The chromosomal distribution of cucumber GATA genes. Table S1: Detail information of the conserved motifs of cucumber GATA proteins. Table S2: The detail syntenic relationships between cucumber and *Arabidopsis* GATA genes. Table S3: The detail syntenic relationships between cucumber and rice GATA genes. Table S4: The downstream target genes of cucumber GATA family genes.
