# Supplementary material for: Genome-Wide Identification, Phylogenetic and Expression Pattern Analysis of GATA Family Genes in Cucumber (Cucumis sativus L.)
Source: Plants (Basel). 2021 Aug 7;10(8):1626. doi: 10.3390/plants10081626 (PMC8401448; doi:10.3390/plants10081626)
Supplement: Supplementary file 1 [file plants-10-01626-s001.zip › Additional File 2 Table S1 Detail information of the conserved motifs of cucumber GATA genes.pdf]

Additional File 2: Table S1 Detail information of the conserved motifs of cucumber GATA proteins.

Motif	E-value	Sequence	Wides	Pfam annotation
motif 1	9.5e-483	HCGTTKTPQWRTGPAGPKTLCNACGVRYK	29	GATA
motif 2	3.60E-215	SGRLVPEYRPAASPTFVSTLHSNSHRKVLEMRR QKE	36	-
motif 3	3.10E-65	NNRGAVDLPPRSSQPQRAASLIRFREKRKERCF DKKIRYTVRKEVALRMQRKKGQFT	57	CCT
motif 4	4.30E-62	SQFSSELCVPYDDLAELEWLSNFVEDSFS	29	-
motif 5	5.80E-38	QLTLSFRGZVYVFDSVSPEKVQAVLLLLGGREV PSGVPSI	40	TIFY
motif 6	2.50E-28	VPGKARSKRSRASPCSW	17	-
motif 7	2.40E-19	KRKQQQDDGLIGWMJPDQGQGFHKVVDEDTS NRSSSGSAISNPESCAQFGGADASDLTGPAQSIV WEAMVPSRKRTCVGRPKQSPVEKLTKDLYSIL REQ	100	-
motif 8	7.10E-19	YQRKFPGEEEQAAILLMALSYGSLY	25	-
motif 9	2.70E-17	DHFFIEDLLDFSNED	15	-
motif 10	2.20E-16	QIKRDDPQHECLQILGNRNSPLCDIDJNDIJNFEE FAKQLTNEEQQZLMKYLPQIDIEEFPESLKSMFE SPQFKENLNSFKQLLSEGVFDFSF	93	ASXH, RPN13_C
