# Supplementary material for: Genome-Wide Identification, Phylogenetic and Expression Pattern Analysis of GATA Family Genes in Cucumber (Cucumis sativus L.)
Source: Plants (Basel). 2021 Aug 7;10(8):1626. doi: 10.3390/plants10081626 (PMC8401448; doi:10.3390/plants10081626)
Supplement: Supplementary file 1 [file plants-10-01626-s001.zip › Additional File 3 Table S2 The Detail syntenic relationships between cucumber and Arabidopsis GATA genes.pdf]

Additional File 3 Table S2 The Detail syntenic relationships between cucumber and *Arabidopsis* GATA genes.

Syntenic relationship	Cucumber GATA gene	*Arabidopsis* GATA gene
1	Csa3G912920	AT4G17570
2	Csa7G447800	AT1G51600
3	Csa7G447800	AT3G21175
4	Csa2G370430	AT4G24470
5	Csa2G373450	AT5G25830
6	Csa3G895650	AT5G25830
7	Csa3G457670	AT4G32890
8	Csa6G504690	AT5G66320
9	Csa7G452960	AT5G25830
10	Csa5G622830	AT3G54810
11	Csa6G504690	AT3G51080
12	Csa3G457670	AT3G60530
13	Csa3G895650	AT4G32890
14	Csa3G895650	AT3G60530
15	Csa3G895650	AT2G45050
16	Csa2G162660	AT3G24050
17	Csa3G457670	AT5G25830
18	Csa3G457670	AT2G45050
19	Csa4G043890	AT5G66320
20	Csa7G452960	AT4G32890
21	Csa3G017200	AT5G47140
22	Csa6G504690	AT4G36240
23	Csa7G452960	AT3G24050
24	Csa7G452960	AT3G60530
25	Csa2G162660	AT4G32890
26	Csa4G043890	AT4G36240
27	Csa4G043890	AT3G51080
28	Csa7G452960	AT2G45050
29	Csa5G622830	AT1G08000
30	Csa2G162660	AT3G51080
31	Csa2G162660	AT4G34680
32	Csa2G162660	AT5G66320
33	Csa2G162660	AT3G60530
34	Csa2G251490	AT4G34680
35	Csa2G251490	AT3G51080
36	Csa4G046650	AT3G50870
37	Csa6G405920	AT3G54810
38	Csa2G251490	AT5G66320
39	Csa6G504690	AT4G34680
40	Csa2G251490	AT4G36240
41	Csa5G622830	AT3G24050
42	Csa5G622830	AT2G28340
43	Csa6G405920	AT3G24050
44	Csa2G251490	AT2G45050
45	Csa4G046650	AT4G36620

46	Csa4G646060	AT5G56860
47	Csa6G405920	AT1G08000
48	Csa1G587970	AT5G56860
49	Csa6G312540	AT3G16870
50	Csa6G312540	AT5G49300
51	Csa6G405920	AT2G28340
52	Csa4G046650	AT2G18380
53	Csa4G646060	AT4G26150
54	Csa6G312540	AT4G16141
55	Csa1G587970	AT4G26150
56	Csa3G165640	AT5G49300
57	Csa3G165640	AT3G06740
58	Csa6G502700	AT3G50870
59	Csa6G502700	AT4G36620
60	Csa6G312540	AT3G06740
61	Csa1G569090	AT5G49300
62	Csa1G587970	AT5G26930
63	Csa6G502700	AT2G18380
64	Csa1G569090	AT5G26930
65	Csa3G165640	AT3G16870
66	Csa3G165640	AT4G16141
67	Csa4G286370	AT3G20750
68	Csa1G569090	AT3G06740
69	Csa6G502700	AT3G06740
70	Csa4G286370	AT5G26930
71	Csa1G569090	AT3G20750
