# Supplementary material for: Genome-Wide Identification, Phylogenetic and Expression Pattern Analysis of GATA Family Genes in Cucumber (Cucumis sativus L.)
Source: Plants (Basel). 2021 Aug 7;10(8):1626. doi: 10.3390/plants10081626 (PMC8401448; doi:10.3390/plants10081626)
Supplement: Supplementary file 1 [file plants-10-01626-s001.zip › Additional File 4 Table S3 The Detail syntenic relationships between cucumber and rice GATA genes.pdf]

Additional File 4 Table S3 The Detail syntenic relationships between cucumber and rice GATA genes.

Syntenic relationship	Cucumber GATA gene	Rice GATA gene
1	Csa7G447800	Os02g05510
2	Csa3G017200	Os04g46020
3	Csa3G457670	Os05g44400
4	Csa3G895650	Os05g44400
5	Csa7G452960	Os05g44400
6	Csa2G373450	Os01g54210
7	Csa3G457670	Os01g54210
8	Csa7G452960	Os01g54210
9	Csa3G912920	Os04g46020
10	Csa3G895650	Os01g54210
11	Csa2G373450	Os05g44400
12	Csa3G457670	Os10g40810
13	Csa7G452960	Os10g40810
14	Csa3G895650	Os10g40810
15	Csa2G370420	Os03g47970
16	Csa2G373450	Os10g40810
17	Csa4G043890	Os02g43150
18	Csa6G504690	Os02g43150
19	Csa7G064580	Os03g47970
20	Csa7G452960	Os02g56250
21	Csa5G622830	Os02g56250
22	Csa5G622830	Os01g54210
23	Csa6G405920	Os02g56250
24	Csa2G251490	Os02g43150
25	Csa2G162660	Os10g40810
26	Csa2G162660	Os12g42970
27	Csa2G251490	Os02g56250
28	Csa2G370420	Os06g48534
29	Csa2G251490	Os12g42970
30	Csa6G405920	Os12g42970
31	Csa4G046650	Os05g49280
32	Csa4G646060	Os02g12790
33	Csa7G064580	Os06g48534
34	Csa3G843820	Os12g42970
35	Csa4G046650	Os05g50270
36	Csa4G046650	Os01g47360
37	Csa6G405920	Os02g43150
38	Csa2G370430	Os03g52450
39	Csa5G622830	Os12g42970
40	Csa2G162660	Os03g05160
41	Csa6G504690	Os03g05160
42	Csa2G251490	Os03g05160
43	Csa1G587970	Os02g12790
44	Csa6G502700	Os01g47360
45	Csa1G587970	Os06g37450

46	Csa2G162660	Os10g32070
47	Csa6G502700	Os05g49280
48	Csa6G502700	Os05g50270
49	Csa4G646060	Os06g37450
50	Csa6G312540	Os05g06340
51	Csa7G405980	Os05g06340
52	Csa3G165640	Os01g74540
53	Csa3G165640	Os05g06340
54	Csa6G312540	Os01g74540
55	Csa7G405980	Os01g24070
56	Csa1G569090	Os01g74540
57	Csa7G405980	Os01g74540
58	Csa6G312540	Os01g24070
59	Csa3G165640	Os01g24070
