# Supplementary material for: Genome-Wide Identification, Phylogenetic and Expression Pattern Analysis of GATA Family Genes in Cucumber (Cucumis sativus L.)
Source: Plants (Basel). 2021 Aug 7;10(8):1626. doi: 10.3390/plants10081626 (PMC8401448; doi:10.3390/plants10081626)
Supplement: Supplementary file 1 [file plants-10-01626-s001.zip › Additional File 5 Table S4 The downstream target genes of cucumber GATA family genes.pdf]

Additional File 5 Table S4 The downstream target genes of cucumber GATA family genes.

Cucumber GATA gene	Downstream target gene	Cucumber GATA gene	Downstream target gene
Csa1G569090	-	Csa2G373450	Csa2G431130
Csa1G587970	-	Csa2G373450	Csa5G013780
Csa2G251490	-	Csa2G373450	Csa5G627080
Csa2G370420	-	Csa2G373450	Csa1G231030
Csa3G017200	-	Csa2G373450	Csa6G505840
Csa3G457670	-	Csa2G373450	Csa6G505850
Csa3G843820	-	Csa2G373450	Csa6G510340
Csa3G912920	-	Csa2G373450	Csa6G515460
Csa4G043890	-	Csa2G373450	Csa6G519510
Csa4G046650	-	Csa2G373450	Csa6G526290
Csa4G286370	-	Csa2G373450	Csa7G428810
Csa4G646060	-	Csa2G373450	Csa3G391900
Csa5G622830	-	Csa2G373450	Csa6G361340
Csa6G312540	-	Csa2G373450	Csa4G607040
Csa6G502700	-	Csa2G373450	Csa4G310190
Csa6G504690	-	Csa2G373450	Csa2G419960
Csa7G064580	-	Csa2G373450	Csa6G358710
Csa7G405980	-	Csa2G373450	Csa7G290450
Csa7G452960	-	Csa2G373450	Csa6G450960
Csa2G162660	Csa5G428440	Csa2G373450	Csa6G452760
Csa2G162660	Csa1G187170	Csa2G373450	Csa1G059750
Csa2G162660	Csa1G186620	Csa2G373450	Csa1G062340
Csa2G162660	Csa5G505790	Csa2G373450	Csa1G062880
Csa2G162660	Csa4G196810	Csa2G373450	Csa1G064740
Csa2G162660	Csa4G193760	Csa2G373450	Csa2G030060
Csa2G162660	Csa4G193240	Csa2G373450	Csa1G422430
Csa2G162660	Csa7G433400	Csa2G373450	Csa4G010960
Csa2G162660	Csa7G433350	Csa2G373450	Csa7G023930
Csa2G162660	Csa6G399760	Csa2G373450	Csa7G023960
Csa2G162660	Csa6G401340	Csa2G373450	Csa7G032270
Csa2G162660	Csa6G401470	Csa2G373450	Csa6G486820
Csa2G162660	Csa6G403570	Csa2G373450	Csa6G490960
Csa2G162660	Csa6G403580	Csa2G373450	Csa6G499140
Csa2G162660	Csa6G404190	Csa2G373450	Csa7G432240
Csa2G162660	Csa6G404230	Csa2G373450	Csa7G431360
Csa2G162660	Csa7G212670	Csa2G373450	Csa4G046810
Csa2G162660	Csa7G213180	Csa2G373450	Csa1G025090
Csa2G162660	Csa6G212860	Csa2G373450	Csa1G031200
Csa2G162660	Csa5G034000	Csa2G373450	Csa4G052640
Csa2G162660	Csa5G517180	Csa2G373450	Csa5G171670
Csa2G162660	Csa5G517760	Csa2G373450	Csa6G105670
Csa2G162660	Csa5G517780	Csa2G373450	Csa4G637790
Csa2G162660	Csa5G517800	Csa2G373450	Csa6G448130
Csa2G162660	Csa5G523690	Csa2G373450	Csa1G071930
Csa2G162660	Csa5G524700	Csa2G373450	Csa1G075000

Csa2G162660	Csa5G524780	Csa2G373450	Csa7G420830
Csa2G162660	Csa5G524840	Csa2G373450	Csa6G008730
Csa2G162660	Csa5G527900	Csa2G373450	Csa6G011660
Csa2G162660	Csa5G529950	Csa2G373450	Csa6G405940
Csa2G162660	Csa5G533470	Csa2G373450	Csa6G190250
Csa2G162660	Csa5G534990	Csa2G373450	Csa3G624060
Csa2G162660	Csa5G548120	Csa2G373450	Csa1G600190
Csa2G162660	Csa5G550250	Csa2G373450	Csa1G604040
Csa2G162660	Csa5G547610	Csa2G373450	Csa7G041300
Csa2G162660	Csa3G331340	Csa2G373450	Csa1G533420
Csa2G162660	Csa3G346900	Csa2G373450	Csa1G000550
Csa2G162660	Csa3G346910	Csa2G373450	Csa1G001400
Csa2G162660	Csa3G363190	Csa2G373450	Csa1G002680
Csa2G162660	Csa3G357110	Csa2G373450	Csa5G636600
Csa2G162660	Csa3G357080	Csa2G373450	Csa5G633830
Csa2G162660	Csa3G354540	Csa2G373450	Csa5G633190
Csa2G162660	Csa5G353650	Csa2G373450	Csa5G175810
Csa2G162660	Csa5G350590	Csa2G373450	Csa1G538820
Csa2G162660	Csa5G349570	Csa2G373450	Csa4G457190
Csa2G162660	Csa2G228390	Csa2G373450	Csa5G315360
Csa2G162660	Csa3G426920	Csa2G373450	Csa3G732490
Csa2G162660	Csa2G431190	Csa2G373450	Csa3G701570
Csa2G162660	Csa4G338940	Csa2G373450	Csa2G075400
Csa2G162660	Csa4G358630	Csa2G373450	Csa2G074150
Csa2G162660	Csa5G002570	Csa2G373450	Csa4G064100
Csa2G162660	Csa5G002050	Csa2G373450	Csa4G056630
Csa2G162660	Csa4G146250	Csa2G373450	Csa1G569180
Csa2G162660	Csa4G141210	Csa2G373450	Csa1G569450
Csa2G162660	Csa6G330470	Csa2G373450	Csa6G421780
Csa2G162660	Csa6G330980	Csa2G373450	Csa6G423350
Csa2G162660	Csa3G435020	Csa2G373450	Csa6G425750
Csa2G162660	Csa5G011740	Csa2G373450	Csa2G326470
Csa2G162660	Csa5G011660	Csa2G373450	Csa3G651770
Csa2G162660	Csa4G192070	Csa2G373450	Csa5G141100
Csa2G162660	Csa7G394000	Csa2G373450	Csa2G151040
Csa2G162660	Csa7G394660	Csa2G373450	Csa1G005600
Csa2G162660	Csa7G395770	Csa2G373450	Csa1G014500
Csa2G162660	Csa5G099490	Csa2G373450	Csa1G084290
Csa2G162660	Csa5G091890	Csa2G373450	Csa1G084280
Csa2G162660	Csa1G666960	Csa2G373450	Csa7G278730
Csa2G162660	Csa4G031030	Csa2G373450	Csa3G809970
Csa2G162660	Csa4G031020	Csa2G373450	Csa3G172990
Csa2G162660	Csa4G028990	Csa2G373450	Csa3G149950
Csa2G162660	Csa5G628140	Csa2G373450	Csa3G129720
Csa2G162660	Csa3G270290	Csa2G373450	Csa3G127820
Csa2G162660	Csa3G264750	Csa2G373450	Csa3G117430
Csa2G162660	Csa3G258160	Csa2G373450	Csa5G579560
Csa2G162660	Csa3G257090	Csa2G373450	Csa5G614670

Csa2G162660	Csa3G252450	Csa2G373450	Csa5G608250
Csa2G162660	Csa3G247880	Csa2G373450	Csa5G606630
Csa2G162660	Csa3G239880	Csa2G373450	Csa5G606570
Csa2G162660	Csa3G238740	Csa2G373450	Csa5G606530
Csa2G162660	Csa3G238730	Csa2G373450	Csa5G606320
Csa2G162660	Csa3G238210	Csa2G373450	Csa5G606270
Csa2G162660	Csa3G236040	Csa2G373450	Csa1G690180
Csa2G162660	Csa3G236030	Csa2G373450	Csa4G646370
Csa2G162660	Csa3G219730	Csa2G373450	Csa3G535620
Csa2G162660	Csa3G219200	Csa2G373450	Csa3G516510
Csa2G162660	Csa5G466370	Csa2G373450	Csa5G160150
Csa2G162660	Csa5G468980	Csa2G373450	Csa5G153120
Csa2G162660	Csa5G471600	Csa2G373450	Csa5G148520
Csa2G162660	Csa5G396020	Csa2G373450	Csa3G740080
Csa2G162660	Csa5G387990	Csa2G373450	Csa3G910700
Csa2G162660	Csa5G416260	Csa2G373450	Csa2G435430
Csa2G162660	Csa5G416270	Csa2G373450	Csa1G166250
Csa2G162660	Csa5G418780	Csa2G373450	Csa3G002830
Csa2G162660	Csa1G231010	Csa2G373450	Csa4G179150
Csa2G162660	Csa4G153800	Csa2G373450	Csa3G822260
Csa2G162660	Csa4G154320	Csa2G373450	Csa3G829130
Csa2G162660	Csa3G039300	Csa2G373450	Csa3G837640
Csa2G162660	Csa3G046220	Csa2G373450	Csa6G087830
Csa2G162660	Csa3G047860	Csa2G373450	Csa6G092550
Csa2G162660	Csa3G061550	Csa2G373450	Csa1G629200
Csa2G162660	Csa3G063640	Csa2G373450	Csa1G657480
Csa2G162660	Csa3G064190	Csa2G373450	Csa4G425740
Csa2G162660	Csa3G064230	Csa2G373450	Csa7G448730
Csa2G162660	Csa3G073790	Csa2G373450	Csa7G448820
Csa2G162660	Csa3G074470	Csa2G373450	Csa7G450720
Csa2G162660	Csa6G504440	Csa2G373450	Csa6G074600
Csa2G162660	Csa6G504460	Csa2G373450	Csa6G067380
Csa2G162660	Csa6G504470	Csa2G373450	Csa6G062280
Csa2G162660	Csa6G504670	Csa2G373450	Csa6G024410
Csa2G162660	Csa6G505300	Csa2G373450	Csa2G292850
Csa2G162660	Csa6G505860	Csa2G373450	Csa5G128230
Csa2G162660	Csa6G505890	Csa2G373450	Csa6G303790
Csa2G162660	Csa6G505990	Csa2G373450	Csa2G169710
Csa2G162660	Csa6G506010	Csa2G373450	Csa2G174120
Csa2G162660	Csa6G507450	Csa2G373450	Csa2G190750
Csa2G162660	Csa6G507470	Csa2G373450	Csa1G050160
Csa2G162660	Csa6G509550	Csa2G373450	Csa2G345920
Csa2G162660	Csa6G509570	Csa2G373450	Csa2G347140
Csa2G162660	Csa6G510350	Csa2G373450	Csa2G348170
Csa2G162660	Csa6G510890	Csa2G373450	Csa2G350260
Csa2G162660	Csa6G510960	Csa2G373450	Csa2G351760
Csa2G162660	Csa6G511110	Csa2G373450	Csa2G354100
Csa2G162660	Csa6G511780	Csa2G373450	Csa4G097650

Csa2G162660	Csa6G512910	Csa2G373450	Csa4G111590
Csa2G162660	Csa6G513430	Csa3G165640	Csa4G193760
Csa2G162660	Csa6G513530	Csa3G165640	Csa1G701990
Csa2G162660	Csa6G514290	Csa3G165640	Csa6G399730
Csa2G162660	Csa6G514810	Csa3G165640	Csa6G400800
Csa2G162660	Csa6G514820	Csa3G165640	Csa6G401340
Csa2G162660	Csa6G515460	Csa3G165640	Csa6G401370
Csa2G162660	Csa6G515480	Csa3G165640	Csa6G401410
Csa2G162660	Csa6G516660	Csa3G165640	Csa6G403580
Csa2G162660	Csa6G516730	Csa3G165640	Csa6G404230
Csa2G162660	Csa6G516780	Csa3G165640	Csa5G420280
Csa2G162660	Csa6G516790	Csa3G165640	Csa7G212110
Csa2G162660	Csa6G517200	Csa3G165640	Csa7G213180
Csa2G162660	Csa6G517400	Csa3G165640	Csa6G212860
Csa2G162660	Csa6G517950	Csa3G165640	Csa5G517200
Csa2G162660	Csa6G517960	Csa3G165640	Csa5G517760
Csa2G162660	Csa6G518040	Csa3G165640	Csa5G523060
Csa2G162660	Csa6G518310	Csa3G165640	Csa5G524700
Csa2G162660	Csa6G518320	Csa3G165640	Csa5G524840
Csa2G162660	Csa6G518350	Csa3G165640	Csa5G533470
Csa2G162660	Csa6G519620	Csa3G165640	Csa3G342350
Csa2G162660	Csa6G520310	Csa3G165640	Csa3G357110
Csa2G162660	Csa6G520350	Csa3G165640	Csa5G353650
Csa2G162660	Csa6G520390	Csa3G165640	Csa2G225320
Csa2G162660	Csa6G520410	Csa3G165640	Csa2G228390
Csa2G162660	Csa6G521010	Csa3G165640	Csa2G432230
Csa2G162660	Csa6G522790	Csa3G165640	Csa2G433360
Csa2G162660	Csa6G522800	Csa3G165640	Csa4G337890
Csa2G162660	Csa6G523330	Csa3G165640	Csa4G338980
Csa2G162660	Csa6G523460	Csa3G165640	Csa4G358630
Csa2G162660	Csa6G524610	Csa3G165640	Csa4G141230
Csa2G162660	Csa6G525420	Csa3G165640	Csa6G330990
Csa2G162660	Csa6G525680	Csa3G165640	Csa6G331010
Csa2G162660	Csa6G526250	Csa3G165640	Csa4G416450
Csa2G162660	Csa6G526450	Csa3G165640	Csa4G192180
Csa2G162660	Csa6G526520	Csa3G165640	Csa7G392940
Csa2G162660	Csa6G526550	Csa3G165640	Csa7G394000
Csa2G162660	Csa6G538090	Csa3G165640	Csa7G394660
Csa2G162660	Csa6G538600	Csa3G165640	Csa5G091890
Csa2G162660	Csa6G538610	Csa3G165640	Csa1G666960
Csa2G162660	Csa6G538700	Csa3G165640	Csa4G031030
Csa2G162660	Csa6G538770	Csa3G165640	Csa4G031020
Csa2G162660	Csa6G538790	Csa3G165640	Csa3G265250
Csa2G162660	Csa3G212490	Csa3G165640	Csa3G264750
Csa2G162660	Csa7G429620	Csa3G165640	Csa3G257090
Csa2G162660	Csa7G429570	Csa3G165640	Csa3G252450
Csa2G162660	Csa7G428280	Csa3G165640	Csa3G247880
Csa2G162660	Csa7G428260	Csa3G165640	Csa3G238740

Csa2G162660	Csa1G560780	Csa3G165640	Csa3G238210
Csa2G162660	Csa1G560810	Csa3G165640	Csa3G238090
Csa2G162660	Csa1G561920	Csa3G165640	Csa3G236030
Csa2G162660	Csa1G568490	Csa3G165640	Csa3G229410
Csa2G162660	Csa1G569110	Csa3G165640	Csa5G471600
Csa2G162660	Csa6G294430	Csa3G165640	Csa5G409610
Csa2G162660	Csa6G290790	Csa3G165640	Csa5G416270
Csa2G162660	Csa6G290830	Csa3G165640	Csa1G231010
Csa2G162660	Csa3G405510	Csa3G165640	Csa3G608170
Csa2G162660	Csa3G402470	Csa3G165640	Csa3G523070
Csa2G162660	Csa3G398920	Csa3G165640	Csa3G041370
Csa2G162660	Csa3G384790	Csa3G165640	Csa3G047810
Csa2G162660	Csa3G383780	Csa3G165640	Csa3G061000
Csa2G162660	Csa3G383760	Csa3G165640	Csa3G063640
Csa2G162660	Csa3G563310	Csa3G165640	Csa6G504460
Csa2G162660	Csa3G585900	Csa3G165640	Csa6G504470
Csa2G162660	Csa3G588470	Csa3G165640	Csa6G504630
Csa2G162660	Csa3G588520	Csa3G165640	Csa6G504660
Csa2G162660	Csa1G435820	Csa3G165640	Csa6G504670
Csa2G162660	Csa1G435790	Csa3G165640	Csa6G505300
Csa2G162660	Csa1G435750	Csa3G165640	Csa6G505810
Csa2G162660	Csa1G616850	Csa3G165640	Csa6G505860
Csa2G162660	Csa7G145970	Csa3G165640	Csa6G507350
Csa2G162660	Csa4G626100	Csa3G165640	Csa6G509570
Csa2G162660	Csa4G625020	Csa3G165640	Csa6G510960
Csa2G162660	Csa4G622820	Csa3G165640	Csa6G512880
Csa2G162660	Csa4G620640	Csa3G165640	Csa6G513530
Csa2G162660	Csa4G620620	Csa3G165640	Csa6G514290
Csa2G162660	Csa4G620600	Csa3G165640	Csa6G514940
Csa2G162660	Csa4G618510	Csa3G165640	Csa6G516520
Csa2G162660	Csa4G618460	Csa3G165640	Csa6G516600
Csa2G162660	Csa4G618430	Csa3G165640	Csa6G516730
Csa2G162660	Csa4G618410	Csa3G165640	Csa6G516790
Csa2G162660	Csa4G595970	Csa3G165640	Csa6G517200
Csa2G162660	Csa3G081930	Csa3G165640	Csa6G517380
Csa2G162660	Csa3G080340	Csa3G165640	Csa6G517410
Csa2G162660	Csa3G078790	Csa3G165640	Csa6G517950
Csa2G162660	Csa3G077680	Csa3G165640	Csa6G517960
Csa2G162660	Csa3G077610	Csa3G165640	Csa6G518350
Csa2G162660	Csa3G076560	Csa3G165640	Csa6G519620
Csa2G162660	Csa3G076550	Csa3G165640	Csa6G519670
Csa2G162660	Csa3G076530	Csa3G165640	Csa6G520390
Csa2G162660	Csa3G076520	Csa3G165640	Csa6G520410
Csa2G162660	Csa3G076010	Csa3G165640	Csa6G525680
Csa2G162660	Csa6G382890	Csa3G165640	Csa6G525700
Csa2G162660	Csa5G652280	Csa3G165640	Csa6G526230
Csa2G162660	Csa5G652230	Csa3G165640	Csa6G526450
Csa2G162660	Csa5G652220	Csa3G165640	Csa6G538600

Csa2G162660	Csa5G652210	Csa3G165640	Csa6G538610
Csa2G162660	Csa5G650630	Csa3G165640	Csa6G538700
Csa2G162660	Csa3G483750	Csa3G165640	Csa3G214020
Csa2G162660	Csa1G305770	Csa3G165640	Csa3G212490
Csa2G162660	Csa1G305780	Csa3G165640	Csa7G430150
Csa2G162660	Csa1G294600	Csa3G165640	Csa7G429570
Csa2G162660	Csa1G295150	Csa3G165640	Csa1G560780
Csa2G162660	Csa4G288080	Csa3G165640	Csa1G568490
Csa2G162660	Csa4G290800	Csa3G165640	Csa6G290830
Csa2G162660	Csa4G292460	Csa3G165640	Csa3G384790
Csa2G162660	Csa4G293000	Csa3G165640	Csa3G383780
Csa2G162660	Csa4G293160	Csa3G165640	Csa3G383760
Csa2G162660	Csa4G293290	Csa3G165640	Csa3G585890
Csa2G162660	Csa4G294400	Csa3G165640	Csa3G588470
Csa2G162660	Csa4G295470	Csa3G165640	Csa1G435750
Csa2G162660	Csa4G295520	Csa3G165640	Csa1G616850
Csa2G162660	Csa4G296210	Csa3G165640	Csa6G362410
Csa2G162660	Csa4G296300	Csa3G165640	Csa6G365160
Csa2G162660	Csa4G303070	Csa3G165640	Csa7G145970
Csa2G162660	Csa4G307960	Csa3G165640	Csa4G627100
Csa2G162660	Csa4G308490	Csa3G165640	Csa4G622750
Csa2G162660	Csa4G310210	Csa3G165640	Csa4G621210
Csa2G162660	Csa4G311220	Csa3G165640	Csa4G608170
Csa2G162660	Csa5G310310	Csa3G165640	Csa3G081930
Csa2G162660	Csa5G310810	Csa3G165640	Csa3G080340
Csa2G162660	Csa4G432970	Csa3G165640	Csa3G077610
Csa2G162660	Csa4G430880	Csa3G165640	Csa3G076550
Csa2G162660	Csa4G429320	Csa3G165640	Csa3G076010
Csa2G162660	Csa6G318710	Csa3G165640	Csa5G652280
Csa2G162660	Csa6G318660	Csa3G165640	Csa5G652210
Csa2G162660	Csa6G318140	Csa3G165640	Csa3G483830
Csa2G162660	Csa6G314100	Csa3G165640	Csa1G305760
Csa2G162660	Csa6G314090	Csa3G165640	Csa4G290200
Csa2G162660	Csa6G312560	Csa3G165640	Csa4G290220
Csa2G162660	Csa2G416770	Csa3G165640	Csa4G290830
Csa2G162660	Csa2G416790	Csa3G165640	Csa4G293060
Csa2G162660	Csa2G422040	Csa3G165640	Csa4G295470
Csa2G162660	Csa2G423550	Csa3G165640	Csa4G295520
Csa2G162660	Csa2G423620	Csa3G165640	Csa4G296210
Csa2G162660	Csa2G423650	Csa3G165640	Csa4G303070
Csa2G162660	Csa2G426800	Csa3G165640	Csa5G308760
Csa2G162660	Csa3G088990	Csa3G165640	Csa5G310310
Csa2G162660	Csa3G094500	Csa3G165640	Csa5G310810
Csa2G162660	Csa3G098040	Csa3G165640	Csa6G319750
Csa2G162660	Csa3G099660	Csa3G165640	Csa6G318660
Csa2G162660	Csa3G099670	Csa3G165640	Csa2G416820
Csa2G162660	Csa3G104900	Csa3G165640	Csa2G419960
Csa2G162660	Csa3G104920	Csa3G165640	Csa3G100800

Csa2G162660	Csa3G106000	Csa3G165640	Csa3G104900
Csa2G162660	Csa3G110020	Csa3G165640	Csa5G593340
Csa2G162660	Csa5G593340	Csa3G165640	Csa5G593330
Csa2G162660	Csa5G593330	Csa3G165640	Csa5G591760
Csa2G162660	Csa5G591780	Csa3G165640	Csa5G590000
Csa2G162660	Csa5G591760	Csa3G165640	Csa5G589890
Csa2G162660	Csa5G591740	Csa3G165640	Csa5G587190
Csa2G162660	Csa5G590080	Csa3G165640	Csa5G586030
Csa2G162660	Csa5G590010	Csa3G165640	Csa5G585990
Csa2G162660	Csa5G590000	Csa3G165640	Csa5G585450
Csa2G162660	Csa5G589890	Csa3G165640	Csa6G338050
Csa2G162660	Csa5G589380	Csa3G165640	Csa6G338660
Csa2G162660	Csa5G589340	Csa3G165640	Csa6G344210
Csa2G162660	Csa5G589290	Csa3G165640	Csa6G349830
Csa2G162660	Csa5G589270	Csa3G165640	Csa6G349850
Csa2G162660	Csa5G586030	Csa3G165640	Csa6G355440
Csa2G162660	Csa5G585450	Csa3G165640	Csa6G356490
Csa2G162660	Csa6G338090	Csa3G165640	Csa6G358100
Csa2G162660	Csa6G338120	Csa3G165640	Csa6G358130
Csa2G162660	Csa6G338660	Csa3G165640	Csa7G354520
Csa2G162660	Csa6G344240	Csa3G165640	Csa7G352410
Csa2G162660	Csa6G349830	Csa3G165640	Csa7G337600
Csa2G162660	Csa6G349850	Csa3G165640	Csa7G336510
Csa2G162660	Csa6G355380	Csa3G165640	Csa7G328310
Csa2G162660	Csa6G355410	Csa3G165640	Csa7G307400
Csa2G162660	Csa6G355430	Csa3G165640	Csa1G423270
Csa2G162660	Csa6G355440	Csa3G165640	Csa6G450420
Csa2G162660	Csa6G355960	Csa3G165640	Csa6G451500
Csa2G162660	Csa6G356490	Csa3G165640	Csa6G452640
Csa2G162660	Csa6G358090	Csa3G165640	Csa6G452740
Csa2G162660	Csa6G358100	Csa3G165640	Csa6G453770
Csa2G162660	Csa6G358130	Csa3G165640	Csa6G453800
Csa2G162660	Csa6G358660	Csa3G165640	Csa7G387730
Csa2G162660	Csa6G358710	Csa3G165640	Csa7G388310
Csa2G162660	Csa7G351890	Csa3G165640	Csa2G234580
Csa2G162660	Csa7G342800	Csa3G165640	Csa2G234600
Csa2G162660	Csa7G341240	Csa3G165640	Csa1G062310
Csa2G162660	Csa7G340200	Csa3G165640	Csa1G062360
Csa2G162660	Csa7G337070	Csa3G165640	Csa1G064710
Csa2G162660	Csa7G336570	Csa3G165640	Csa1G064840
Csa2G162660	Csa7G336510	Csa3G165640	Csa1G065380
Csa2G162660	Csa7G336450	Csa3G165640	Csa1G065390
Csa2G162660	Csa7G332900	Csa3G165640	Csa1G065960
Csa2G162660	Csa7G329350	Csa3G165640	Csa6G427970
Csa2G162660	Csa7G324150	Csa3G165640	Csa6G430650
Csa2G162660	Csa7G320000	Csa3G165640	Csa6G430710
Csa2G162660	Csa7G307400	Csa3G165640	Csa2G406720
Csa2G162660	Csa7G302360	Csa3G165640	Csa2G406700

Csa2G162660	Csa1G423270	Csa3G165640	Csa2G406060
Csa2G162660	Csa6G450420	Csa3G165640	Csa2G404800
Csa2G162660	Csa6G451500	Csa3G165640	Csa2G403140
Csa2G162660	Csa6G452020	Csa3G165640	Csa2G402130
Csa2G162660	Csa6G452090	Csa3G165640	Csa2G401380
Csa2G162660	Csa6G452640	Csa3G165640	Csa2G061530
Csa2G162660	Csa6G452660	Csa3G165640	Csa2G060530
Csa2G162660	Csa6G453770	Csa3G165640	Csa2G059740
Csa2G162660	Csa6G453800	Csa3G165640	Csa2G049330
Csa2G162660	Csa6G454320	Csa3G165640	Csa2G036620
Csa2G162660	Csa6G454420	Csa3G165640	Csa2G035380
Csa2G162660	Csa6G454480	Csa3G165640	Csa2G022830
Csa2G162660	Csa6G476120	Csa3G165640	Csa2G021740
Csa2G162660	Csa6G476070	Csa3G165640	Csa2G020990
Csa2G162660	Csa7G387730	Csa3G165640	Csa2G020970
Csa2G162660	Csa7G388340	Csa3G165640	Csa1G423030
Csa2G162660	Csa7G389480	Csa3G165640	Csa1G256710
Csa2G162660	Csa7G390000	Csa3G165640	Csa4G026870
Csa2G162660	Csa7G390130	Csa3G165640	Csa4G025190
Csa2G162660	Csa7G390220	Csa3G165640	Csa4G022910
Csa2G162660	Csa7G391240	Csa3G165640	Csa4G022870
Csa2G162660	Csa7G392380	Csa3G165640	Csa4G022350
Csa2G162660	Csa2G234550	Csa3G165640	Csa4G016490
Csa2G162660	Csa2G234570	Csa3G165640	Csa4G016450
Csa2G162660	Csa2G234580	Csa3G165640	Csa4G012480
Csa2G162660	Csa1G059750	Csa3G165640	Csa4G011820
Csa2G162660	Csa1G060260	Csa3G165640	Csa4G011800
Csa2G162660	Csa1G062350	Csa3G165640	Csa4G009890
Csa2G162660	Csa1G062360	Csa3G165640	Csa4G008800
Csa2G162660	Csa1G062930	Csa3G165640	Csa4G007720
Csa2G162660	Csa1G063590	Csa3G165640	Csa4G006340
Csa2G162660	Csa1G064700	Csa3G165640	Csa4G006310
Csa2G162660	Csa1G064710	Csa3G165640	Csa4G006190
Csa2G162660	Csa1G064740	Csa3G165640	Csa4G006180
Csa2G162660	Csa1G064850	Csa3G165640	Csa4G004970
Csa2G162660	Csa1G065380	Csa3G165640	Csa4G004810
Csa2G162660	Csa1G065390	Csa3G165640	Csa4G003730
Csa2G162660	Csa1G065950	Csa3G165640	Csa4G001940
Csa2G162660	Csa1G066510	Csa3G165640	Csa4G001810
Csa2G162660	Csa1G066530	Csa3G165640	Csa4G001590
Csa2G162660	Csa6G430650	Csa3G165640	Csa4G001540
Csa2G162660	Csa6G430710	Csa3G165640	Csa1G044900
Csa2G162660	Csa6G432270	Csa3G165640	Csa1G045890
Csa2G162660	Csa2G406780	Csa3G165640	Csa1G045940
Csa2G162660	Csa2G406720	Csa3G165640	Csa1G046030
Csa2G162660	Csa2G406630	Csa3G165640	Csa1G046820
Csa2G162660	Csa2G406060	Csa3G165640	Csa1G046880
Csa2G162660	Csa2G405030	Csa3G165640	Csa7G019910

Csa2G162660	Csa2G404940	Csa3G165640	Csa7G021920
Csa2G162660	Csa2G404900	Csa3G165640	Csa7G025190
Csa2G162660	Csa2G403690	Csa3G165640	Csa7G030500
Csa2G162660	Csa2G402630	Csa3G165640	Csa7G032280
Csa2G162660	Csa2G402130	Csa3G165640	Csa7G033370
Csa2G162660	Csa2G402070	Csa3G165640	Csa7G033390
Csa2G162660	Csa2G402020	Csa3G165640	Csa6G483370
Csa2G162660	Csa2G401380	Csa3G165640	Csa6G483450
Csa2G162660	Csa2G401330	Csa3G165640	Csa6G484020
Csa2G162660	Csa2G401250	Csa3G165640	Csa6G486990
Csa2G162660	Csa2G060390	Csa3G165640	Csa6G487680
Csa2G162660	Csa2G060370	Csa3G165640	Csa6G487740
Csa2G162660	Csa2G048330	Csa3G165640	Csa6G487810
Csa2G162660	Csa2G047830	Csa3G165640	Csa6G491620
Csa2G162660	Csa2G036620	Csa3G165640	Csa6G491710
Csa2G162660	Csa2G036070	Csa3G165640	Csa6G493900
Csa2G162660	Csa2G035540	Csa3G165640	Csa6G494950
Csa2G162660	Csa2G035480	Csa3G165640	Csa6G496410
Csa2G162660	Csa2G035380	Csa3G165640	Csa6G497210
Csa2G162660	Csa2G034490	Csa3G165640	Csa6G497350
Csa2G162660	Csa2G033940	Csa3G165640	Csa6G497370
Csa2G162660	Csa2G031710	Csa3G165640	Csa6G499110
Csa2G162660	Csa2G023860	Csa3G165640	Csa6G499170
Csa2G162660	Csa2G022250	Csa3G165640	Csa6G502000
Csa2G162660	Csa2G021740	Csa3G165640	Csa7G432470
Csa2G162660	Csa2G021700	Csa3G165640	Csa7G432460
Csa2G162660	Csa2G021690	Csa3G165640	Csa7G431970
Csa2G162660	Csa2G021560	Csa3G165640	Csa4G046680
Csa2G162660	Csa2G020990	Csa3G165640	Csa4G047370
Csa2G162660	Csa2G020920	Csa3G165640	Csa4G048550
Csa2G162660	Csa2G020850	Csa3G165640	Csa4G049050
Csa2G162660	Csa2G014830	Csa3G165640	Csa4G050130
Csa2G162660	Csa1G418240	Csa3G165640	Csa4G050830
Csa2G162660	Csa1G418250	Csa3G165640	Csa4G050840
Csa2G162660	Csa1G423090	Csa3G165640	Csa4G051370
Csa2G162660	Csa1G423150	Csa3G165640	Csa4G051390
Csa2G162660	Csa1G256780	Csa3G165640	Csa3G182070
Csa2G162660	Csa4G025740	Csa3G165640	Csa3G183980
Csa2G162660	Csa4G025120	Csa3G165640	Csa3G186680
Csa2G162660	Csa4G023020	Csa3G165640	Csa3G198470
Csa2G162660	Csa4G022870	Csa3G165640	Csa2G252070
Csa2G162660	Csa4G017170	Csa3G165640	Csa2G251480
Csa2G162660	Csa4G017090	Csa3G165640	Csa2G250940
Csa2G162660	Csa4G016450	Csa3G165640	Csa2G250430
Csa2G162660	Csa4G015820	Csa3G165640	Csa2G249910
Csa2G162660	Csa4G015130	Csa3G165640	Csa2G249260
Csa2G162660	Csa4G013540	Csa3G165640	Csa2G248760
Csa2G162660	Csa4G012480	Csa3G165640	Csa2G248740

Csa2G162660	Csa4G012410	Csa3G165640	Csa2G248710
Csa2G162660	Csa4G011830	Csa3G165640	Csa2G248700
Csa2G162660	Csa4G011020	Csa3G165640	Csa2G245480
Csa2G162660	Csa4G009900	Csa3G165640	Csa2G245430
Csa2G162660	Csa4G009890	Csa3G165640	Csa2G238780
Csa2G162660	Csa4G009870	Csa3G165640	Csa2G237710
Csa2G162660	Csa4G008800	Csa3G165640	Csa2G237180
Csa2G162660	Csa4G008240	Csa3G165640	Csa1G024240
Csa2G162660	Csa4G007620	Csa3G165640	Csa1G024920
Csa2G162660	Csa4G007080	Csa3G165640	Csa1G024930
Csa2G162660	Csa4G006440	Csa3G165640	Csa1G025040
Csa2G162660	Csa4G006350	Csa3G165640	Csa1G028050
Csa2G162660	Csa4G006310	Csa3G165640	Csa1G031830
Csa2G162660	Csa4G006190	Csa3G165640	Csa1G032450
Csa2G162660	Csa4G006180	Csa3G165640	Csa1G032470
Csa2G162660	Csa4G004980	Csa3G165640	Csa1G033040
Csa2G162660	Csa4G004960	Csa3G165640	Csa1G033200
Csa2G162660	Csa4G003730	Csa3G165640	Csa1G039270
Csa2G162660	Csa4G003090	Csa3G165640	Csa3G110640
Csa2G162660	Csa4G001940	Csa3G165640	Csa4G055320
Csa2G162660	Csa4G001870	Csa3G165640	Csa4G052640
Csa2G162660	Csa4G001830	Csa3G165640	Csa5G172860
Csa2G162660	Csa4G001820	Csa3G165640	Csa5G171700
Csa2G162660	Csa4G001810	Csa3G165640	Csa2G005920
Csa2G162660	Csa4G001740	Csa3G165640	Csa2G005360
Csa2G162660	Csa4G001590	Csa3G165640	Csa2G000660
Csa2G162660	Csa4G001540	Csa3G165640	Csa2G000440
Csa2G162660	Csa4G000870	Csa3G165640	Csa2G000260
Csa2G162660	Csa1G044880	Csa3G165640	Csa1G524680
Csa2G162660	Csa1G044900	Csa3G165640	Csa1G524640
Csa2G162660	Csa1G044930	Csa3G165640	Csa1G523060
Csa2G162660	Csa1G045460	Csa3G165640	Csa6G139770
Csa2G162660	Csa1G045500	Csa3G165640	Csa6G137590
Csa2G162660	Csa1G045520	Csa3G165640	Csa6G134390
Csa2G162660	Csa1G045660	Csa3G165640	Csa6G133760
Csa2G162660	Csa1G045710	Csa3G165640	Csa6G133660
Csa2G162660	Csa1G045760	Csa3G165640	Csa6G128590
Csa2G162660	Csa1G045800	Csa3G165640	Csa6G126260
Csa2G162660	Csa1G045940	Csa3G165640	Csa6G123970
Csa2G162660	Csa1G045990	Csa3G165640	Csa6G123470
Csa2G162660	Csa1G046030	Csa3G165640	Csa6G118340
Csa2G162660	Csa1G046100	Csa3G165640	Csa6G115610
Csa2G162660	Csa1G046130	Csa3G165640	Csa6G109800
Csa2G162660	Csa7G017150	Csa3G165640	Csa6G109660
Csa2G162660	Csa7G023940	Csa3G165640	Csa6G108560
Csa2G162660	Csa7G023950	Csa3G165640	Csa1G042430
Csa2G162660	Csa7G024030	Csa3G165640	Csa1G042520
Csa2G162660	Csa7G024090	Csa3G165640	Csa1G042850

Csa2G162660	Csa7G024150	Csa3G165640	Csa1G042890
Csa2G162660	Csa7G031660	Csa3G165640	Csa1G042960
Csa2G162660	Csa7G033370	Csa3G165640	Csa1G042970
Csa2G162660	Csa7G033390	Csa3G165640	Csa4G627820
Csa2G162660	Csa6G483370	Csa3G165640	Csa4G628340
Csa2G162660	Csa6G483450	Csa3G165640	Csa4G637130
Csa2G162660	Csa6G484010	Csa3G165640	Csa4G637730
Csa2G162660	Csa6G484020	Csa3G165640	Csa4G639130
Csa2G162660	Csa6G486720	Csa3G165640	Csa6G445040
Csa2G162660	Csa6G486880	Csa3G165640	Csa6G445180
Csa2G162660	Csa6G486990	Csa3G165640	Csa6G446440
Csa2G162660	Csa6G487560	Csa3G165640	Csa6G446460
Csa2G162660	Csa6G487670	Csa3G165640	Csa6G448680
Csa2G162660	Csa6G487700	Csa3G165640	Csa6G448690
Csa2G162660	Csa6G487740	Csa3G165640	Csa4G361880
Csa2G162660	Csa6G488330	Csa3G165640	Csa1G071260
Csa2G162660	Csa6G490240	Csa3G165640	Csa1G071270
Csa2G162660	Csa6G490880	Csa3G165640	Csa1G074980
Csa2G162660	Csa6G491060	Csa3G165640	Csa1G074990
Csa2G162660	Csa6G493850	Csa3G165640	Csa1G077220
Csa2G162660	Csa6G493890	Csa3G165640	Csa1G077760
Csa2G162660	Csa6G495010	Csa3G165640	Csa7G428210
Csa2G162660	Csa6G495850	Csa3G165640	Csa7G428180
Csa2G162660	Csa6G497030	Csa3G165640	Csa7G426560
Csa2G162660	Csa6G497120	Csa3G165640	Csa7G426450
Csa2G162660	Csa6G497210	Csa3G165640	Csa7G420820
Csa2G162660	Csa6G497350	Csa3G165640	Csa7G419570
Csa2G162660	Csa6G497900	Csa3G165640	Csa5G169080
Csa2G162660	Csa6G499200	Csa3G165640	Csa5G169040
Csa2G162660	Csa6G499220	Csa3G165640	Csa5G168980
Csa2G162660	Csa6G499870	Csa3G165640	Csa5G168790
Csa2G162660	Csa6G500460	Csa3G165640	Csa5G167210
Csa2G162660	Csa6G500490	Csa3G165640	Csa4G312800
Csa2G162660	Csa6G500500	Csa3G165640	Csa4G334690
Csa2G162660	Csa6G500540	Csa3G165640	Csa4G336250
Csa2G162660	Csa6G500590	Csa3G165640	Csa4G337340
Csa2G162660	Csa6G500620	Csa3G165640	Csa2G006060
Csa2G162660	Csa6G501230	Csa3G165640	Csa2G007960
Csa2G162660	Csa7G433280	Csa3G165640	Csa2G007970
Csa2G162660	Csa7G432650	Csa3G165640	Csa2G008080
Csa2G162660	Csa7G432470	Csa3G165640	Csa2G009340
Csa2G162660	Csa7G432460	Csa3G165640	Csa2G011560
Csa2G162660	Csa7G432350	Csa3G165640	Csa2G011600
Csa2G162660	Csa7G432270	Csa3G165640	Csa2G012130
Csa2G162660	Csa7G432130	Csa3G165640	Csa1G058140
Csa2G162660	Csa7G432120	Csa3G165640	Csa1G058080
Csa2G162660	Csa7G431970	Csa3G165640	Csa1G051760
Csa2G162660	Csa4G046650	Csa3G165640	Csa1G051730

Csa2G162660	Csa4G046660	Csa3G165640	Csa1G051660
Csa2G162660	Csa4G046670	Csa3G165640	Csa6G001720
Csa2G162660	Csa4G046680	Csa3G165640	Csa6G004520
Csa2G162660	Csa4G047370	Csa3G165640	Csa6G004600
Csa2G162660	Csa4G047890	Csa3G165640	Csa6G008060
Csa2G162660	Csa4G047930	Csa3G165640	Csa6G011720
Csa2G162660	Csa4G049050	Csa3G165640	Csa6G407120
Csa2G162660	Csa4G050140	Csa3G165640	Csa6G406540
Csa2G162660	Csa4G050230	Csa3G165640	Csa6G188040
Csa2G162660	Csa4G050780	Csa3G165640	Csa6G182130
Csa2G162660	Csa4G050800	Csa3G165640	Csa6G182110
Csa2G162660	Csa4G050830	Csa3G165640	Csa6G181580
Csa2G162660	Csa4G050840	Csa3G165640	Csa3G623980
Csa2G162660	Csa4G051370	Csa3G165640	Csa3G610280
Csa2G162660	Csa4G051390	Csa3G165640	Csa1G597110
Csa2G162660	Csa3G181950	Csa3G165640	Csa1G597810
Csa2G162660	Csa3G182110	Csa3G165640	Csa1G600100
Csa2G162660	Csa3G182780	Csa3G165640	Csa1G600140
Csa2G162660	Csa3G183900	Csa3G165640	Csa1G600190
Csa2G162660	Csa3G183910	Csa3G165640	Csa1G601010
Csa2G162660	Csa3G186680	Csa3G165640	Csa1G604600
Csa2G162660	Csa3G187250	Csa3G165640	Csa1G605660
Csa2G162660	Csa3G188330	Csa3G165640	Csa1G613460
Csa2G162660	Csa3G188340	Csa3G165640	Csa1G613620
Csa2G162660	Csa2G252070	Csa3G165640	Csa1G614650
Csa2G162660	Csa2G251510	Csa3G165640	Csa5G223110
Csa2G162660	Csa2G251480	Csa3G165640	Csa5G223020
Csa2G162660	Csa2G250940	Csa3G165640	Csa5G220900
Csa2G162660	Csa2G250430	Csa3G165640	Csa5G217670
Csa2G162660	Csa2G249910	Csa3G165640	Csa5G215130
Csa2G162660	Csa2G249900	Csa3G165640	CsaUNG017140
Csa2G162660	Csa2G249260	Csa3G165640	Csa1G530130
Csa2G162660	Csa2G248740	Csa3G165640	Csa1G530140
Csa2G162660	Csa2G248710	Csa3G165640	Csa7G048000
Csa2G162660	Csa2G248700	Csa3G165640	Csa7G044210
Csa2G162660	Csa2G247600	Csa3G165640	Csa7G043610
Csa2G162660	Csa2G246520	Csa3G165640	Csa7G043020
Csa2G162660	Csa2G239400	Csa3G165640	Csa7G041880
Csa2G162660	Csa2G239380	Csa3G165640	Csa3G646510
Csa2G162660	Csa2G238780	Csa3G165640	Csa3G644860
Csa2G162660	Csa1G024210	Csa3G165640	Csa3G638540
Csa2G162660	Csa1G024920	Csa3G165640	Csa3G634360
Csa2G162660	Csa1G024930	Csa3G165640	Csa3G629740
Csa2G162660	Csa1G024940	Csa3G165640	Csa3G627690
Csa2G162660	Csa1G025040	Csa3G165640	Csa1G533530
Csa2G162660	Csa1G025090	Csa3G165640	Csa1G533660
Csa2G162660	Csa1G025240	Csa3G165640	Csa1G533690
Csa2G162660	Csa1G025780	Csa3G165640	Csa4G268070

Csa2G162660	Csa1G031210	Csa3G165640	Csa4G269200
Csa2G162660	Csa1G031830	Csa3G165640	Csa4G279840
Csa2G162660	Csa1G032450	Csa3G165640	Csa2G307850
Csa2G162660	Csa1G033000	Csa3G165640	Csa1G588520
Csa2G162660	Csa1G033040	Csa3G165640	Csa1G589090
Csa2G162660	Csa1G033200	Csa3G165640	Csa1G589650
Csa2G162660	Csa1G038930	Csa3G165640	Csa1G589720
Csa2G162660	Csa3G110050	Csa3G165640	Csa3G595200
Csa2G162660	Csa3G110660	Csa3G165640	Csa1G000600
Csa2G162660	Csa3G110700	Csa3G165640	Csa1G000610
Csa2G162660	Csa3G111250	Csa3G165640	Csa1G000700
Csa2G162660	Csa3G112780	Csa3G165640	Csa1G000730
Csa2G162660	Csa4G055370	Csa3G165640	Csa1G002090
Csa2G162660	Csa4G055350	Csa3G165640	Csa1G002120
Csa2G162660	Csa4G055320	Csa3G165640	Csa5G640510
Csa2G162660	Csa4G052740	Csa3G165640	Csa5G640000
Csa2G162660	Csa4G052690	Csa3G165640	Csa5G638340
Csa2G162660	Csa2G005970	Csa3G165640	Csa5G637790
Csa2G162660	Csa2G004680	Csa3G165640	Csa5G637670
Csa2G162660	Csa2G000920	Csa3G165640	Csa5G637160
Csa2G162660	Csa2G000800	Csa3G165640	Csa5G636570
Csa2G162660	Csa2G000730	Csa3G165640	Csa5G633190
Csa2G162660	Csa2G000660	Csa3G165640	Csa5G631520
Csa2G162660	Csa2G000560	Csa3G165640	Csa5G631510
Csa2G162660	Csa2G000440	Csa3G165640	Csa5G630810
Csa2G162660	Csa2G000350	Csa3G165640	Csa2G364570
Csa2G162660	Csa2G000330	Csa3G165640	Csa2G369840
Csa2G162660	Csa2G000260	Csa3G165640	Csa2G370350
Csa2G162660	Csa2G000100	Csa3G165640	Csa2G372160
Csa2G162660	Csa1G523610	Csa3G165640	Csa2G372740
Csa2G162660	Csa1G515990	Csa3G165640	Csa2G372750
Csa2G162660	Csa6G139250	Csa3G165640	Csa2G372820
Csa2G162660	Csa6G139240	Csa3G165640	Csa2G375760
Csa2G162660	Csa6G137590	Csa3G165640	Csa2G376790
Csa2G162660	Csa6G136000	Csa3G165640	Csa2G379300
Csa2G162660	Csa6G134900	Csa3G165640	Csa2G379350
Csa2G162660	Csa6G134390	Csa3G165640	Csa2G382590
Csa2G162660	Csa6G133700	Csa3G165640	Csa2G382710
Csa2G162660	Csa6G133680	Csa3G165640	Csa2G385070
Csa2G162660	Csa6G128640	Csa3G165640	Csa1G570240
Csa2G162660	Csa6G128590	Csa3G165640	Csa1G572440
Csa2G162660	Csa6G128040	Csa3G165640	Csa5G182130
Csa2G162660	Csa6G127430	Csa3G165640	Csa5G180840
Csa2G162660	Csa6G126260	Csa3G165640	Csa5G179750
Csa2G162660	Csa6G120430	Csa3G165640	Csa5G179740
Csa2G162660	Csa6G120420	Csa3G165640	Csa5G177650
Csa2G162660	Csa6G115610	Csa3G165640	Csa5G175780
Csa2G162660	Csa6G111360	Csa3G165640	Csa5G173520

Csa2G162660	Csa6G110310	Csa3G165640	Csa5G173440
Csa2G162660	Csa6G109660	Csa3G165640	Csa1G542510
Csa2G162660	Csa6G108590	Csa3G165640	Csa2G223720
Csa2G162660	Csa6G108570	Csa3G165640	Csa2G224260
Csa2G162660	Csa6G106760	Csa3G165640	Csa2G264610
Csa2G162660	Csa1G042440	Csa3G165640	Csa4G454660
Csa2G162660	Csa1G042520	Csa3G165640	Csa1G257340
Csa2G162660	Csa1G042590	Csa3G165640	Csa1G257870
Csa2G162660	Csa1G042610	Csa3G165640	Csa1G276450
Csa2G162660	Csa1G042630	Csa3G165640	Csa6G191550
Csa2G162660	Csa1G042830	Csa3G165640	Csa6G190450
Csa2G162660	Csa1G042850	Csa3G165640	Csa5G317890
Csa2G162660	Csa1G042890	Csa3G165640	Csa5G023910
Csa2G162660	Csa1G042960	Csa3G165640	Csa4G129620
Csa2G162660	Csa1G042970	Csa3G165640	Csa3G733980
Csa2G162660	Csa1G042980	Csa3G165640	Csa3G733320
Csa2G162660	Csa1G043010	Csa3G165640	Csa3G732510
Csa2G162660	Csa4G630520	Csa3G165640	Csa3G710850
Csa2G162660	Csa4G630530	Csa3G165640	Csa3G707170
Csa2G162660	Csa4G630550	Csa3G165640	Csa2G076000
Csa2G162660	Csa4G631610	Csa3G165640	Csa2G070330
Csa2G162660	Csa4G632110	Csa3G165640	Csa4G064070
Csa2G162660	Csa4G637730	Csa3G165640	Csa4G063460
Csa2G162660	Csa4G637740	Csa3G165640	Csa4G061850
Csa2G162660	Csa4G638390	Csa3G165640	Csa4G056710
Csa2G162660	Csa4G639130	Csa3G165640	Csa1G181420
Csa2G162660	Csa4G639740	Csa3G165640	Csa1G181410
Csa2G162660	Csa6G433800	Csa3G165640	Csa1G181340
Csa2G162660	Csa6G434340	Csa3G165640	Csa1G180760
Csa2G162660	Csa6G446440	Csa3G165640	Csa1G179740
Csa2G162660	Csa6G446460	Csa3G165640	Csa7G038680
Csa2G162660	Csa6G446530	Csa3G165640	Csa1G183570
Csa2G162660	Csa6G447060	Csa3G165640	Csa1G570110
Csa2G162660	Csa6G448690	Csa3G165640	Csa1G570140
Csa2G162660	Csa6G449240	Csa3G165640	Csa5G505150
Csa2G162660	Csa4G361880	Csa3G165640	Csa5G070180
Csa2G162660	Csa4G361900	Csa3G165640	Csa6G410060
Csa2G162660	Csa1G070620	Csa3G165640	Csa6G411220
Csa2G162660	Csa1G071260	Csa3G165640	Csa6G419450
Csa2G162660	Csa1G071270	Csa3G165640	Csa6G421780
Csa2G162660	Csa1G071290	Csa3G165640	Csa6G423450
Csa2G162660	Csa1G073620	Csa3G165640	Csa6G425040
Csa2G162660	Csa1G073750	Csa3G165640	Csa6G425750
Csa2G162660	Csa1G074920	Csa3G165640	Csa6G426370
Csa2G162660	Csa1G075060	Csa3G165640	Csa5G284510
Csa2G162660	Csa1G077130	Csa3G165640	Csa6G385070
Csa2G162660	Csa1G077150	Csa3G165640	Csa2G099470
Csa2G162660	Csa1G077760	Csa3G165640	Csa5G140530

Csa2G162660	Csa6G502080	Csa3G165640	Csa2G153590
Csa2G162660	Csa6G502590	Csa3G165640	Csa2G160620
Csa2G162660	Csa6G502870	Csa3G165640	Csa3G696880
Csa2G162660	Csa7G428180	Csa3G165640	Csa1G003510
Csa2G162660	Csa7G426560	Csa3G165640	Csa1G004040
Csa2G162660	Csa7G420850	Csa3G165640	Csa1G004890
Csa2G162660	Csa7G420820	Csa3G165640	Csa1G008410
Csa2G162660	Csa7G419590	Csa3G165640	Csa1G009680
Csa2G162660	Csa7G419550	Csa3G165640	Csa1G009790
Csa2G162660	Csa7G414510	Csa3G165640	Csa1G009900
Csa2G162660	Csa7G414410	Csa3G165640	Csa1G011450
Csa2G162660	Csa5G169080	Csa3G165640	Csa1G014360
Csa2G162660	Csa5G168790	Csa3G165640	Csa1G015040
Csa2G162660	Csa5G167210	Csa3G165640	Csa1G015610
Csa2G162660	Csa5G167200	Csa3G165640	Csa1G015690
Csa2G162660	Csa5G167150	Csa3G165640	Csa1G015760
Csa2G162660	Csa5G167090	Csa3G165640	Csa1G015770
Csa2G162660	Csa5G167080	Csa3G165640	Csa1G015880
Csa2G162660	Csa5G166400	Csa3G165640	Csa1G021950
Csa2G162660	Csa5G165850	Csa3G165640	Csa1G022500
Csa2G162660	Csa5G165320	Csa3G165640	Csa1G023060
Csa2G162660	Csa5G165270	Csa3G165640	Csa1G023070
Csa2G162660	Csa5G165230	Csa3G165640	Csa1G023080
Csa2G162660	Csa5G165220	Csa3G165640	Csa4G038740
Csa2G162660	Csa4G312280	Csa3G165640	Csa4G038730
Csa2G162660	Csa4G312850	Csa3G165640	Csa4G036590
Csa2G162660	Csa4G314440	Csa3G165640	Csa4G036580
Csa2G162660	Csa4G314480	Csa3G165640	Csa5G139760
Csa2G162660	Csa4G337260	Csa3G165640	Csa5G139350
Csa2G162660	Csa4G337320	Csa3G165640	Csa5G139210
Csa2G162660	Csa4G337360	Csa3G165640	Csa5G139180
Csa2G162660	Csa2G006050	Csa3G165640	Csa5G139170
Csa2G162660	Csa2G006060	Csa3G165640	Csa5G139100
Csa2G162660	Csa2G006130	Csa3G165640	Csa7G413390
Csa2G162660	Csa2G007960	Csa3G165640	Csa7G407700
Csa2G162660	Csa2G008080	Csa3G165640	Csa7G407680
Csa2G162660	Csa2G009290	Csa3G165640	Csa7G407610
Csa2G162660	Csa2G009340	Csa3G165640	Csa7G407510
Csa2G162660	Csa2G009370	Csa3G165640	Csa7G406990
Csa2G162660	Csa2G009480	Csa3G165640	Csa7G405820
Csa2G162660	Csa2G009530	Csa3G165640	Csa7G396430
Csa2G162660	Csa2G009550	Csa3G165640	Csa1G120410
Csa2G162660	Csa2G010290	Csa3G165640	Csa1G088470
Csa2G162660	Csa2G010300	Csa3G165640	Csa1G096100
Csa2G162660	Csa2G011480	Csa3G165640	Csa1G446900
Csa2G162660	Csa2G011490	Csa3G165640	Csa1G462040
Csa2G162660	Csa2G012140	Csa3G165640	Csa1G478080
Csa2G162660	Csa2G013280	Csa3G165640	Csa1G479630

Csa2G162660	Csa2G013320	Csa3G165640	Csa1G479640
Csa2G162660	Csa1G058060	Csa3G165640	Csa1G480180
Csa2G162660	Csa1G051760	Csa3G165640	Csa1G495290
Csa2G162660	Csa1G051730	Csa3G165640	Csa1G505930
Csa2G162660	Csa1G051690	Csa3G165640	Csa1G505960
Csa2G162660	Csa1G051650	Csa3G165640	Csa7G062880
Csa2G162660	Csa6G000030	Csa3G165640	Csa7G065130
Csa2G162660	Csa6G001220	Csa3G165640	Csa7G067530
Csa2G162660	Csa6G001780	Csa3G165640	Csa7G067540
Csa2G162660	Csa6G003410	Csa3G165640	Csa7G069720
Csa2G162660	Csa6G004520	Csa3G165640	Csa7G071440
Csa2G162660	Csa6G004600	Csa3G165640	Csa7G071470
Csa2G162660	Csa6G006690	Csa3G165640	Csa7G071670
Csa2G162660	Csa6G006710	Csa3G165640	Csa7G072780
Csa2G162660	Csa6G006830	Csa3G165640	Csa7G072870
Csa2G162660	Csa6G006840	Csa3G165640	Csa7G072880
Csa2G162660	Csa6G007450	Csa3G165640	Csa7G073450
Csa2G162660	Csa6G008060	Csa3G165640	Csa7G073460
Csa2G162660	Csa6G008630	Csa3G165640	Csa7G073610
Csa2G162660	Csa6G008720	Csa3G165640	Csa7G074880
Csa2G162660	Csa6G009460	Csa3G165640	Csa2G340400
Csa2G162660	Csa6G010010	Csa3G165640	Csa2G336140
Csa2G162660	Csa6G011070	Csa3G165640	Csa2G336080
Csa2G162660	Csa6G011720	Csa3G165640	Csa2G334540
Csa2G162660	Csa6G013350	Csa3G165640	Csa2G361860
Csa2G162660	Csa6G406540	Csa3G165640	Csa2G361850
Csa2G162660	Csa6G405890	Csa3G165640	Csa2G361640
Csa2G162660	Csa6G405310	Csa3G165640	Csa2G360810
Csa2G162660	Csa6G190350	Csa3G165640	Csa2G360740
Csa2G162660	Csa6G187970	Csa3G165640	Csa2G360700
Csa2G162660	Csa6G185240	Csa3G165640	Csa2G358880
Csa2G162660	Csa6G182110	Csa3G165640	Csa2G271430
Csa2G162660	Csa6G181580	Csa3G165640	Csa2G271380
Csa2G162660	Csa6G181520	Csa3G165640	Csa2G270870
Csa2G162660	Csa3G625090	Csa3G165640	Csa2G270810
Csa2G162660	Csa3G624060	Csa3G165640	Csa2G270770
Csa2G162660	Csa3G624050	Csa3G165640	Csa2G270160
Csa2G162660	Csa3G623980	Csa3G165640	Csa2G270150
Csa2G162660	Csa3G623960	Csa3G165640	Csa7G201830
Csa2G162660	Csa3G621430	Csa3G165640	Csa7G201920
Csa2G162660	Csa3G611380	Csa3G165640	Csa7G207020
Csa2G162660	Csa3G611350	Csa3G165640	Csa1G715230
Csa2G162660	Csa3G611340	Csa3G165640	Csa5G650570
Csa2G162660	Csa3G610820	Csa3G165640	Csa5G650520
Csa2G162660	Csa3G610800	Csa3G165640	Csa5G650430
Csa2G162660	Csa3G610280	Csa3G165640	Csa5G649910
Csa2G162660	Csa1G596440	Csa3G165640	Csa5G647360
Csa2G162660	Csa1G597020	Csa3G165640	Csa5G643250

Csa2G162660	Csa1G597090	Csa3G165640	Csa7G234110
Csa2G162660	Csa1G599380	Csa3G165640	Csa7G234670
Csa2G162660	Csa1G599420	Csa3G165640	Csa7G267920
Csa2G162660	Csa1G600190	Csa3G165640	Csa7G278200
Csa2G162660	Csa1G600770	Csa3G165640	Csa7G281380
Csa2G162660	Csa1G601000	Csa3G165640	Csa3G775240
Csa2G162660	Csa1G601010	Csa3G165640	Csa3G775270
Csa2G162660	Csa1G601520	Csa3G165640	Csa3G776860
Csa2G162660	Csa1G605110	Csa3G165640	Csa3G778360
Csa2G162660	Csa1G605720	Csa3G165640	Csa3G778370
Csa2G162660	Csa1G612900	Csa3G165640	Csa3G778990
Csa2G162660	Csa1G613460	Csa3G165640	Csa3G779010
Csa2G162660	Csa1G613510	Csa3G165640	Csa3G782760
Csa2G162660	Csa1G614140	Csa3G165640	Csa3G783840
Csa2G162660	Csa1G614650	Csa3G165640	Csa3G790960
Csa2G162660	Csa5G224130	Csa3G165640	Csa3G792040
Csa2G162660	Csa5G218860	Csa3G165640	Csa3G808380
Csa2G162660	Csa5G218180	Csa3G165640	Csa3G809400
Csa2G162660	Csa5G217680	Csa3G165640	Csa3G207390
Csa2G162660	Csa5G217670	Csa3G165640	Csa6G327940
Csa2G162660	Csa5G217170	Csa3G165640	Csa5G375760
Csa2G162660	Csa5G215130	Csa3G165640	Csa5G381780
Csa2G162660	CsaUNG017140	Csa3G165640	Csa3G685690
Csa2G162660	Csa7G047980	Csa3G165640	Csa3G687750
Csa2G162660	Csa7G047370	Csa3G165640	Csa1G226410
Csa2G162660	Csa7G047360	Csa3G165640	Csa2G302120
Csa2G162660	Csa7G047310	Csa3G165640	Csa2G295420
Csa2G162660	Csa7G047290	Csa3G165640	Csa3G176320
Csa2G162660	Csa7G046680	Csa3G165640	Csa3G174570
Csa2G162660	Csa7G045560	Csa3G165640	Csa3G172930
Csa2G162660	Csa7G045490	Csa3G165640	Csa3G171230
Csa2G162660	Csa7G044910	Csa3G165640	Csa3G168390
Csa2G162660	Csa7G044870	Csa3G165640	Csa3G166340
Csa2G162660	Csa7G044270	Csa3G165640	Csa3G166300
Csa2G162660	Csa7G043630	Csa3G165640	Csa3G166280
Csa2G162660	Csa7G043610	Csa3G165640	Csa3G153150
Csa2G162660	Csa7G043580	Csa3G165640	Csa3G150120
Csa2G162660	Csa7G043560	Csa3G165640	Csa3G144740
Csa2G162660	Csa7G043040	Csa3G165640	Csa3G135110
Csa2G162660	Csa7G043020	Csa3G165640	Csa3G134880
Csa2G162660	Csa7G041370	Csa3G165640	Csa3G133970
Csa2G162660	Csa3G646640	Csa3G165640	Csa3G133360
Csa2G162660	Csa3G646510	Csa3G165640	Csa3G133290
Csa2G162660	Csa3G640580	Csa3G165640	Csa3G133260
Csa2G162660	Csa3G638010	Csa3G165640	Csa3G133180
Csa2G162660	Csa3G636440	Csa3G165640	Csa3G133110
Csa2G162660	Csa3G636400	Csa3G165640	Csa3G129750
Csa2G162660	Csa3G635360	Csa3G165640	Csa3G129630

Csa2G162660	Csa3G634360	Csa3G165640	Csa3G127170
Csa2G162660	Csa3G633280	Csa3G165640	Csa3G126860
Csa2G162660	Csa7G219200	Csa3G165640	Csa3G126810
Csa2G162660	Csa1G532380	Csa3G165640	Csa3G126210
Csa2G162660	Csa1G533480	Csa3G165640	Csa3G126130
Csa2G162660	Csa1G533500	Csa3G165640	Csa3G123190
Csa2G162660	Csa1G533510	Csa3G165640	Csa3G119820
Csa2G162660	Csa1G533660	Csa3G165640	Csa3G119580
Csa2G162660	Csa1G534740	Csa3G165640	Csa3G119560
Csa2G162660	Csa4G268070	Csa3G165640	Csa3G118730
Csa2G162660	Csa4G268080	Csa3G165640	Csa3G118150
Csa2G162660	Csa4G269150	Csa3G165640	Csa2G108690
Csa2G162660	Csa4G269760	Csa3G165640	Csa2G115750
Csa2G162660	Csa4G279830	Csa3G165640	Csa2G116260
Csa2G162660	Csa4G280560	Csa3G165640	Csa2G122000
Csa2G162660	Csa4G285730	Csa3G165640	Csa3G416660
Csa2G162660	Csa2G309370	Csa3G165640	Csa4G414410
Csa2G162660	Csa1G588520	Csa3G165640	Csa4G649660
Csa2G162660	Csa1G589090	Csa3G165640	Csa4G651780
Csa2G162660	Csa1G589140	Csa3G165640	Csa4G651840
Csa2G162660	Csa1G589650	Csa3G165640	Csa4G652670
Csa2G162660	Csa1G590270	Csa3G165640	Csa4G652740
Csa2G162660	Csa3G600010	Csa3G165640	Csa4G653410
Csa2G162660	Csa3G598900	Csa3G165640	Csa4G653480
Csa2G162660	Csa3G595200	Csa3G165640	Csa4G658500
Csa2G162660	Csa1G000050	Csa3G165640	Csa4G664250
Csa2G162660	Csa1G000600	Csa3G165640	Csa4G664260
Csa2G162660	Csa1G000610	Csa3G165640	Csa4G664300
Csa2G162660	Csa1G000700	Csa3G165640	Csa4G664500
Csa2G162660	Csa1G001330	Csa3G165640	Csa4G088750
Csa2G162660	Csa1G002060	Csa3G165640	Csa4G088720
Csa2G162660	Csa1G002120	Csa3G165640	Csa4G377170
Csa2G162660	Csa1G002130	Csa3G165640	Csa5G494390
Csa2G162660	Csa1G002680	Csa3G165640	Csa5G497020
Csa2G162660	Csa1G002730	Csa3G165640	Csa5G272920
Csa2G162660	Csa5G640510	Csa3G165640	Csa5G269890
Csa2G162660	Csa5G638420	Csa3G165640	Csa5G571440
Csa2G162660	Csa5G638340	Csa3G165640	Csa5G576610
Csa2G162660	Csa5G637790	Csa3G165640	Csa5G576650
Csa2G162660	Csa5G637160	Csa3G165640	Csa5G576660
Csa2G162660	Csa5G636570	Csa3G165640	Csa5G576670
Csa2G162660	Csa5G636480	Csa3G165640	Csa5G577370
Csa2G162660	Csa5G632060	Csa3G165640	Csa5G577450
Csa2G162660	Csa5G631520	Csa3G165640	Csa5G577960
Csa2G162660	Csa5G631510	Csa3G165640	Csa3G764550
Csa2G162660	Csa5G630930	Csa3G165640	Csa1G433060
Csa2G162660	Csa5G630810	Csa3G165640	Csa1G433050
Csa2G162660	Csa5G630770	Csa3G165640	Csa1G427530

Csa2G162660	Csa5G630730	Csa3G165640	Csa5G613450
Csa2G162660	Csa2G364570	Csa3G165640	Csa5G612900
Csa2G162660	Csa2G367210	Csa3G165640	Csa5G610520
Csa2G162660	Csa2G367240	Csa3G165640	Csa5G610420
Csa2G162660	Csa2G368950	Csa3G165640	Csa5G610360
Csa2G162660	Csa2G368980	Csa3G165640	Csa5G609810
Csa2G162660	Csa2G369750	Csa3G165640	Csa5G609740
Csa2G162660	Csa2G369840	Csa3G165640	Csa5G608570
Csa2G162660	Csa2G370350	Csa3G165640	Csa5G608320
Csa2G162660	Csa2G370390	Csa3G165640	Csa5G608250
Csa2G162660	Csa2G372160	Csa3G165640	Csa5G608190
Csa2G162660	Csa2G372740	Csa3G165640	Csa5G608140
Csa2G162660	Csa2G372750	Csa3G165640	Csa5G608020
Csa2G162660	Csa2G372770	Csa3G165640	Csa5G607450
Csa2G162660	Csa2G372820	Csa3G165640	Csa5G606780
Csa2G162660	Csa2G372870	Csa3G165640	Csa5G606750
Csa2G162660	Csa2G373450	Csa3G165640	Csa5G606590
Csa2G162660	Csa2G373470	Csa3G165640	Csa5G606580
Csa2G162660	Csa2G373570	Csa3G165640	Csa5G605730
Csa2G162660	Csa2G375750	Csa3G165640	Csa5G605140
Csa2G162660	Csa2G375760	Csa3G165640	Csa5G605080
Csa2G162660	Csa2G376790	Csa3G165640	Csa5G604230
Csa2G162660	Csa2G379090	Csa3G165640	Csa5G601610
Csa2G162660	Csa2G379300	Csa3G165640	Csa5G601500
Csa2G162660	Csa2G379360	Csa3G165640	Csa5G600940
Csa2G162660	Csa2G379370	Csa3G165640	Csa5G598740
Csa2G162660	Csa2G382520	Csa3G165640	Csa5G598670
Csa2G162660	Csa2G382560	Csa3G165640	Csa5G598060
Csa2G162660	Csa2G382580	Csa3G165640	Csa1G575110
Csa2G162660	Csa2G382590	Csa3G165640	Csa1G575100
Csa2G162660	Csa2G382610	Csa3G165640	Csa1G575040
Csa2G162660	Csa2G382710	Csa3G165640	Csa1G575000
Csa2G162660	Csa2G382790	Csa3G165640	Csa1G574980
Csa2G162660	Csa2G383370	Csa3G165640	Csa1G573610
Csa2G162660	Csa2G383390	Csa3G165640	Csa1G690230
Csa2G162660	Csa2G383920	Csa3G165640	Csa1G690280
Csa2G162660	Csa2G384430	Csa3G165640	Csa1G103270
Csa2G162660	Csa2G385060	Csa3G165640	Csa1G320310
Csa2G162660	Csa2G385070	Csa3G165640	Csa3G490860
Csa2G162660	Csa1G570240	Csa3G165640	Csa4G000550
Csa2G162660	Csa1G571860	Csa3G165640	Csa4G000530
Csa2G162660	Csa1G572390	Csa3G165640	Csa4G646070
Csa2G162660	Csa1G572440	Csa3G165640	Csa4G646130
Csa2G162660	Csa1G572450	Csa3G165640	Csa3G509940
Csa2G162660	Csa5G190480	Csa3G165640	Csa5G162040
Csa2G162660	Csa5G184810	Csa3G165640	Csa5G158560
Csa2G162660	Csa5G182130	Csa3G165640	Csa5G157310
Csa2G162660	Csa5G180840	Csa3G165640	Csa5G157240

Csa2G162660	Csa5G179740	Csa3G165640	Csa5G156110
Csa2G162660	Csa5G179230	Csa3G165640	Csa5G155580
Csa2G162660	Csa5G177040	Csa3G165640	Csa5G154200
Csa2G162660	Csa5G175910	Csa3G165640	Csa5G152890
Csa2G162660	Csa5G175780	Csa3G165640	Csa5G152860
Csa2G162660	Csa5G175690	Csa3G165640	Csa5G150970
Csa2G162660	Csa5G174590	Csa3G165640	Csa5G150420
Csa2G162660	Csa5G173520	Csa3G165640	Csa5G150390
Csa2G162660	Csa5G173460	Csa3G165640	Csa5G148680
Csa2G162660	Csa5G173440	Csa3G165640	Csa5G148610
Csa2G162660	Csa5G172930	Csa3G165640	Csa3G740820
Csa2G162660	Csa1G537360	Csa3G165640	Csa3G740220
Csa2G162660	Csa1G537580	Csa3G165640	Csa3G739560
Csa2G162660	Csa1G538210	Csa3G165640	Csa3G739040
Csa2G162660	Csa1G538790	Csa3G165640	Csa3G738990
Csa2G162660	Csa1G538800	Csa3G165640	Csa3G738980
Csa2G162660	Csa1G538820	Csa3G165640	Csa3G736960
Csa2G162660	Csa1G555080	Csa3G165640	Csa3G736900
Csa2G162660	Csa2G223720	Csa3G165640	Csa3G736820
Csa2G162660	Csa2G224260	Csa3G165640	Csa3G736730
Csa2G162660	Csa2G264610	Csa3G165640	Csa5G623840
Csa2G162660	Csa2G264590	Csa3G165640	Csa5G623520
Csa2G162660	Csa2G264060	Csa3G165640	Csa5G622500
Csa2G162660	Csa2G264000	Csa3G165640	Csa5G616890
Csa2G162660	Csa2G263870	Csa3G165640	Csa3G878750
Csa2G162660	Csa4G443120	Csa3G165640	Csa3G878780
Csa2G162660	Csa4G454660	Csa3G165640	Csa3G878940
Csa2G162660	Csa1G277450	Csa3G165640	Csa3G880550
Csa2G162660	Csa1G257870	Csa3G165640	Csa3G881790
Csa2G162660	Csa1G257910	Csa3G165640	Csa3G881820
Csa2G162660	Csa1G276450	Csa3G165640	Csa3G881910
Csa2G162660	Csa6G191580	Csa3G165640	Csa3G882970
Csa2G162660	Csa6G190480	Csa3G165640	Csa3G883020
Csa2G162660	Csa5G317890	Csa3G165640	Csa3G889750
Csa2G162660	Csa5G023880	Csa3G165640	Csa3G889760
Csa2G162660	Csa5G023910	Csa3G165640	Csa3G889780
Csa2G162660	Csa4G129620	Csa3G165640	Csa3G889830
Csa2G162660	Csa4G129600	Csa3G165640	Csa3G889910
Csa2G162660	Csa4G129560	Csa3G165640	Csa3G891660
Csa2G162660	Csa4G122810	Csa3G165640	Csa3G893440
Csa2G162660	Csa4G119770	Csa3G165640	Csa3G894460
Csa2G162660	Csa3G734880	Csa3G165640	Csa3G895680
Csa2G162660	Csa3G734330	Csa3G165640	Csa3G895690
Csa2G162660	Csa3G734270	Csa3G165640	Csa3G895870
Csa2G162660	Csa3G734230	Csa3G165640	Csa3G901000
Csa2G162660	Csa3G734180	Csa3G165640	Csa3G901040
Csa2G162660	Csa3G734060	Csa3G165640	Csa3G902320
Csa2G162660	Csa3G733360	Csa3G165640	Csa3G902390

Csa2G162660	Csa3G733280	Csa3G165640	Csa3G902400
Csa2G162660	Csa3G733240	Csa3G165640	Csa3G903520
Csa2G162660	Csa3G732650	Csa3G165640	Csa3G910720
Csa2G162660	Csa3G732440	Csa3G165640	Csa3G912370
Csa2G162660	Csa3G731190	Csa3G165640	Csa3G914030
Csa2G162660	Csa3G731150	Csa3G165640	Csa2G324420
Csa2G162660	Csa3G730770	Csa3G165640	Csa2G437040
Csa2G162660	Csa3G730750	Csa3G165640	Csa2G435510
Csa2G162660	Csa3G728130	Csa3G165640	Csa1G173210
Csa2G162660	Csa3G728060	Csa3G165640	Csa1G165730
Csa2G162660	Csa3G727960	Csa3G165640	Csa3G038170
Csa2G162660	Csa3G722880	Csa3G165640	Csa3G019360
Csa2G162660	Csa3G710850	Csa3G165640	Csa3G017260
Csa2G162660	Csa3G710220	Csa3G165640	Csa3G011850
Csa2G162660	Csa3G710210	Csa3G165640	Csa3G011750
Csa2G162660	Csa3G708170	Csa3G165640	Csa3G011730
Csa2G162660	Csa3G707170	Csa3G165640	Csa3G009520
Csa2G162660	Csa2G079650	Csa3G165640	Csa3G009460
Csa2G162660	Csa2G076000	Csa3G165640	Csa3G008910
Csa2G162660	Csa2G074260	Csa3G165640	Csa3G008330
Csa2G162660	Csa2G074100	Csa3G165640	Csa3G006660
Csa2G162660	Csa4G064100	Csa3G165640	Csa3G005540
Csa2G162660	Csa4G063460	Csa3G165640	Csa3G002900
Csa2G162660	Csa4G061860	Csa3G165640	Csa3G002830
Csa2G162660	Csa4G056750	Csa3G165640	Csa3G002590
Csa2G162660	Csa4G056730	Csa3G165640	Csa3G002500
Csa2G162660	Csa4G056710	Csa3G165640	Csa3G002490
Csa2G162660	Csa4G056680	Csa3G165640	Csa6G309960
Csa2G162660	Csa4G056620	Csa3G165640	Csa6G309980
Csa2G162660	Csa1G181480	Csa3G165640	Csa6G014890
Csa2G162660	Csa1G181420	Csa3G165640	Csa6G014820
Csa2G162660	Csa1G181340	Csa3G165640	Csa6G014730
Csa2G162660	Csa1G180760	Csa3G165640	Csa6G014480
Csa2G162660	Csa1G179740	Csa3G165640	Csa6G376230
Csa2G162660	Csa7G038680	Csa3G165640	Csa6G381850
Csa2G162660	Csa1G569220	Csa3G165640	Csa4G182220
Csa2G162660	Csa1G569460	Csa3G165640	Csa1G700690
Csa2G162660	Csa1G570090	Csa3G165640	Csa1G701380
Csa2G162660	Csa1G570110	Csa3G165640	Csa1G701390
Csa2G162660	Csa5G077190	Csa3G165640	Csa1G615710
Csa2G162660	Csa6G408800	Csa3G165640	Csa1G616250
Csa2G162660	Csa6G409360	Csa3G165640	Csa5G441650
Csa2G162660	Csa6G409910	Csa3G165640	Csa3G812170
Csa2G162660	Csa6G410060	Csa3G165640	Csa3G812230
Csa2G162660	Csa6G410630	Csa3G165640	Csa3G812750
Csa2G162660	Csa6G411210	Csa3G165640	Csa3G816030
Csa2G162660	Csa6G411220	Csa3G165640	Csa3G816080
Csa2G162660	Csa6G411230	Csa3G165640	Csa3G816170

Csa2G162660	Csa6G421600	Csa3G165640	Csa3G819910
Csa2G162660	Csa6G423360	Csa3G165640	Csa3G820500
Csa2G162660	Csa6G423460	Csa3G165640	Csa3G822190
Csa2G162660	Csa6G423470	Csa3G165640	Csa3G822250
Csa2G162660	Csa6G425040	Csa3G165640	Csa3G822390
Csa2G162660	Csa6G425140	Csa3G165640	Csa3G823050
Csa2G162660	Csa6G425790	Csa3G165640	Csa3G824200
Csa2G162660	Csa6G425800	Csa3G165640	Csa3G824890
Csa2G162660	Csa6G425840	Csa3G165640	Csa3G825010
Csa2G162660	Csa6G426880	Csa3G165640	Csa3G827250
Csa2G162660	Csa6G385070	Csa3G165640	Csa3G827290
Csa2G162660	Csa2G099470	Csa3G165640	Csa3G827300
Csa2G162660	Csa2G100580	Csa3G165640	Csa3G829120
Csa2G162660	Csa4G669220	Csa3G165640	Csa3G829150
Csa2G162660	Csa3G651820	Csa3G165640	Csa3G829170
Csa2G162660	Csa3G652380	Csa3G165640	Csa3G836440
Csa2G162660	Csa3G664570	Csa3G165640	Csa3G836500
Csa2G162660	Csa3G681150	Csa3G165640	Csa3G838700
Csa2G162660	Csa5G140530	Csa3G165640	Csa3G838750
Csa2G162660	Csa5G141060	Csa3G165640	Csa3G840410
Csa2G162660	Csa5G146260	Csa3G165640	Csa3G840960
Csa2G162660	Csa5G146320	Csa3G165640	Csa3G841480
Csa2G162660	Csa5G146820	Csa3G165640	Csa3G842000
Csa2G162660	Csa5G146890	Csa3G165640	Csa3G842740
Csa2G162660	Csa5G146960	Csa3G165640	Csa3G847610
Csa2G162660	Csa2G139850	Csa3G165640	Csa3G847620
Csa2G162660	Csa2G149450	Csa3G165640	Csa3G848820
Csa2G162660	Csa2G151030	Csa3G165640	Csa3G850530
Csa2G162660	Csa2G153580	Csa3G165640	Csa3G855310
Csa2G162660	Csa2G160620	Csa3G165640	Csa3G855410
Csa2G162660	Csa3G697900	Csa3G165640	Csa3G856010
Csa2G162660	Csa3G696880	Csa3G165640	Csa3G859720
Csa2G162660	Csa7G049270	Csa3G165640	Csa3G866450
Csa2G162660	Csa7G048620	Csa3G165640	Csa3G872080
Csa2G162660	Csa1G002770	Csa3G165640	Csa3G872160
Csa2G162660	Csa1G002780	Csa3G165640	Csa7G058560
Csa2G162660	Csa1G003500	Csa3G165640	Csa7G051410
Csa2G162660	Csa1G004230	Csa3G165640	Csa6G087720
Csa2G162660	Csa1G004910	Csa3G165640	Csa6G087940
Csa2G162660	Csa1G004930	Csa3G165640	Csa6G087970
Csa2G162660	Csa1G005600	Csa3G165640	Csa6G087980
Csa2G162660	Csa1G005650	Csa3G165640	Csa6G088000
Csa2G162660	Csa1G005690	Csa3G165640	Csa6G088010
Csa2G162660	Csa1G006290	Csa3G165640	Csa6G088150
Csa2G162660	Csa1G008410	Csa3G165640	Csa6G089270
Csa2G162660	Csa1G008470	Csa3G165640	Csa6G091270
Csa2G162660	Csa1G009590	Csa3G165640	Csa6G096440
Csa2G162660	Csa1G009660	Csa3G165640	Csa6G103540

Csa2G162660	Csa1G009680	Csa3G165640	Csa1G267270
Csa2G162660	Csa1G009710	Csa3G165640	Csa4G639770
Csa2G162660	Csa1G011440	Csa3G165640	Csa4G639870
Csa2G162660	Csa1G011590	Csa3G165640	Csa4G639900
Csa2G162660	Csa1G014360	Csa3G165640	Csa4G641650
Csa2G162660	Csa1G014490	Csa3G165640	Csa4G642540
Csa2G162660	Csa1G015040	Csa3G165640	Csa4G645830
Csa2G162660	Csa1G015580	Csa3G165640	Csa4G646020
Csa2G162660	Csa1G015610	Csa3G165640	Csa4G646060
Csa2G162660	Csa1G015660	Csa3G165640	Csa1G629050
Csa2G162660	Csa1G015690	Csa3G165640	Csa1G630300
Csa2G162660	Csa1G015780	Csa3G165640	Csa1G637960
Csa2G162660	Csa1G015800	Csa3G165640	Csa1G638460
Csa2G162660	Csa1G015860	Csa3G165640	Csa1G638510
Csa2G162660	Csa1G015880	Csa3G165640	Csa1G650630
Csa2G162660	Csa1G021950	Csa3G165640	Csa1G652290
Csa2G162660	Csa1G021960	Csa3G165640	Csa1G654900
Csa2G162660	Csa1G023070	Csa3G165640	Csa1G655950
Csa2G162660	Csa1G023080	Csa3G165640	Csa1G660180
Csa2G162660	Csa1G154060	Csa3G165640	Csa4G563700
Csa2G162660	Csa1G145860	Csa3G165640	Csa4G551130
Csa2G162660	Csa1G134270	Csa3G165640	Csa4G427280
Csa2G162660	Csa1G132060	Csa3G165640	Csa4G420210
Csa2G162660	Csa4G044460	Csa3G165640	Csa7G446900
Csa2G162660	Csa4G043950	Csa3G165640	Csa7G446920
Csa2G162660	Csa4G043840	Csa3G165640	Csa7G446970
Csa2G162660	Csa4G038770	Csa3G165640	Csa7G446990
Csa2G162660	Csa4G038740	Csa3G165640	Csa7G447760
Csa2G162660	Csa4G038730	Csa3G165640	Csa7G447970
Csa2G162660	Csa4G037610	Csa3G165640	Csa7G451350
Csa2G162660	Csa4G036580	Csa3G165640	Csa7G452070
Csa2G162660	Csa5G139450	Csa3G165640	Csa6G081510
Csa2G162660	Csa5G139370	Csa3G165640	Csa6G081440
Csa2G162660	Csa5G139210	Csa3G165640	Csa6G080340
Csa2G162660	Csa5G139170	Csa3G165640	Csa6G080320
Csa2G162660	Csa5G139160	Csa3G165640	Csa6G078630
Csa2G162660	Csa5G139100	Csa3G165640	Csa6G078530
Csa2G162660	Csa5G139030	Csa3G165640	Csa6G078510
Csa2G162660	Csa5G139010	Csa3G165640	Csa6G076800
Csa2G162660	Csa7G407820	Csa3G165640	Csa6G076720
Csa2G162660	Csa7G407730	Csa3G165640	Csa6G074590
Csa2G162660	Csa7G407680	Csa3G165640	Csa6G067420
Csa2G162660	Csa7G407610	Csa3G165640	Csa6G057180
Csa2G162660	Csa7G406990	Csa3G165640	Csa6G057130
Csa2G162660	Csa7G405970	Csa3G165640	Csa6G056510
Csa2G162660	Csa7G405820	Csa3G165640	Csa6G056490
Csa2G162660	Csa7G397030	Csa3G165640	Csa6G053320
Csa2G162660	Csa7G397010	Csa3G165640	Csa6G052770

Csa2G162660	Csa1G086390	Csa3G165640	Csa6G046410
Csa2G162660	Csa1G086920	Csa3G165640	Csa6G046330
Csa2G162660	Csa1G088440	Csa3G165640	Csa6G046300
Csa2G162660	Csa1G088450	Csa3G165640	Csa6G042440
Csa2G162660	Csa1G088470	Csa3G165640	Csa6G041170
Csa2G162660	Csa1G096100	Csa3G165640	Csa6G030440
Csa2G162660	Csa1G096640	Csa3G165640	Csa6G022310
Csa2G162660	Csa1G103260	Csa3G165640	Csa6G017090
Csa2G162660	Csa1G464560	Csa3G165640	Csa2G292810
Csa2G162660	Csa1G470430	Csa3G165640	Csa2G292780
Csa2G162660	Csa1G475980	Csa3G165640	Csa2G287110
Csa2G162660	Csa1G479630	Csa3G165640	Csa2G277000
Csa2G162660	Csa1G479640	Csa3G165640	Csa2G277600
Csa2G162660	Csa1G480180	Csa3G165640	Csa5G201320
Csa2G162660	Csa1G481210	Csa3G165640	Csa7G014450
Csa2G162660	Csa1G481730	Csa3G165640	Csa7G012400
Csa2G162660	Csa1G497300	Csa3G165640	Csa5G118170
Csa2G162660	Csa1G502880	Csa3G165640	Csa4G164350
Csa2G162660	Csa1G505930	Csa3G165640	Csa5G114620
Csa2G162660	Csa1G505960	Csa3G165640	Csa7G004050
Csa2G162660	Csa1G507460	Csa3G165640	Csa3G748780
Csa2G162660	Csa7G062860	Csa3G165640	Csa3G747630
Csa2G162660	Csa7G064580	Csa3G165640	Csa3G746620
Csa2G162660	Csa7G065130	Csa3G165640	Csa3G745020
Csa2G162660	Csa7G066300	Csa3G165640	Csa6G301070
Csa2G162660	Csa7G066310	Csa3G165640	Csa6G301600
Csa2G162660	Csa7G066320	Csa3G165640	Csa2G315900
Csa2G162660	Csa7G067530	Csa3G165640	Csa7G010300
Csa2G162660	Csa7G069700	Csa3G165640	Csa7G009170
Csa2G162660	Csa7G069720	Csa3G165640	Csa7G009150
Csa2G162660	Csa7G071440	Csa3G165640	Csa2G165670
Csa2G162660	Csa7G071480	Csa3G165640	Csa2G169770
Csa2G162660	Csa7G071560	Csa3G165640	Csa2G171830
Csa2G162660	Csa7G071630	Csa3G165640	Csa2G178720
Csa2G162660	Csa7G071670	Csa3G165640	Csa2G190780
Csa2G162660	Csa7G072760	Csa3G165640	Csa2G193330
Csa2G162660	Csa7G072770	Csa3G165640	Csa2G193360
Csa2G162660	Csa7G072780	Csa3G165640	Csa2G193380
Csa2G162660	Csa7G072870	Csa3G165640	Csa6G160680
Csa2G162660	Csa7G073420	Csa3G165640	Csa6G157050
Csa2G162660	Csa7G073450	Csa3G165640	Csa6G147500
Csa2G162660	Csa7G073520	Csa3G165640	Csa2G211450
Csa2G162660	Csa7G073650	Csa3G165640	Csa2G213970
Csa2G162660	Csa7G073710	Csa3G165640	Csa2G215490
Csa2G162660	Csa7G074880	Csa3G165640	Csa7G363030
Csa2G162660	Csa7G074890	Csa3G165640	Csa7G372270
Csa2G162660	Csa7G108300	Csa3G165640	Csa7G372350
Csa2G162660	Csa1G084290	Csa3G165640	Csa7G374590

Csa2G162660	Csa2G340400	Csa3G165640	Csa7G378470
Csa2G162660	Csa2G338880	Csa3G165640	Csa7G378500
Csa2G162660	Csa2G338830	Csa3G165640	Csa1G050000
Csa2G162660	Csa2G337760	Csa3G165640	Csa1G050450
Csa2G162660	Csa2G336150	Csa3G165640	Csa1G050470
Csa2G162660	Csa2G336080	Csa3G165640	Csa1G050500
Csa2G162660	Csa5G264270	Csa3G165640	Csa5G021310
Csa2G162660	Csa2G363030	Csa3G165640	Csa2G346000
Csa2G162660	Csa2G362450	Csa3G165640	Csa2G348840
Csa2G162660	Csa2G361880	Csa3G165640	Csa2G348900
Csa2G162660	Csa2G361870	Csa3G165640	Csa2G349090
Csa2G162660	Csa2G361850	Csa3G165640	Csa2G351810
Csa2G162660	Csa2G361840	Csa3G165640	Csa2G354050
Csa2G162660	Csa2G361760	Csa3G165640	Csa2G354780
Csa2G162660	Csa2G361580	Csa3G165640	Csa2G354920
Csa2G162660	Csa2G360750	Csa3G165640	Csa4G107420
Csa2G162660	Csa2G360740	Csa3G895650	Csa6G401320
Csa2G162660	Csa2G359970	Csa3G895650	Csa2G431130
Csa2G162660	Csa2G359950	Csa3G895650	Csa1G678040
Csa2G162660	Csa2G358880	Csa3G895650	Csa1G229510
Csa2G162660	Csa2G271380	Csa3G895650	Csa1G231030
Csa2G162660	Csa2G270870	Csa3G895650	Csa6G526290
Csa2G162660	Csa2G270150	Csa3G895650	Csa6G538790
Csa2G162660	Csa1G391590	Csa3G895650	Csa4G291360
Csa2G162660	Csa7G201830	Csa3G895650	Csa4G292440
Csa2G162660	Csa7G201870	Csa3G895650	Csa4G293160
Csa2G162660	Csa7G201920	Csa3G895650	Csa4G310190
Csa2G162660	Csa1G704590	Csa3G895650	Csa2G427860
Csa2G162660	Csa1G714680	Csa3G895650	Csa7G336510
Csa2G162660	Csa1G715230	Csa3G895650	Csa6G476050
Csa2G162660	Csa1G716240	Csa3G895650	Csa1G062350
Csa2G162660	Csa5G650570	Csa3G895650	Csa1G062880
Csa2G162660	Csa5G650480	Csa3G895650	Csa2G036080
Csa2G162660	Csa5G649910	Csa3G895650	Csa2G035430
Csa2G162660	Csa5G647360	Csa3G895650	Csa4G022330
Csa2G162660	Csa5G646750	Csa3G895650	Csa4G011750
Csa2G162660	Csa5G646670	Csa3G895650	Csa4G011730
Csa2G162660	Csa5G645090	Csa3G895650	Csa1G045820
Csa2G162660	Csa5G644520	Csa3G895650	Csa6G490960
Csa2G162660	Csa5G644020	Csa3G895650	Csa7G433210
Csa2G162660	Csa5G643380	Csa3G895650	Csa2G249910
Csa2G162660	Csa5G642710	Csa3G895650	Csa1G024860
Csa2G162660	Csa5G642170	Csa3G895650	Csa1G025090
Csa2G162660	Csa5G642140	Csa3G895650	Csa1G031200
Csa2G162660	Csa7G234110	Csa3G895650	Csa6G133760
Csa2G162660	Csa7G234150	Csa3G895650	Csa6G105670
Csa2G162660	Csa7G234160	Csa3G895650	Csa6G104070
Csa2G162660	Csa7G234710	Csa3G895650	Csa6G446340

Csa2G162660	Csa7G235250	Csa3G895650	Csa1G072990
Csa2G162660	Csa7G237300	Csa3G895650	Csa7G420740
Csa2G162660	Csa7G239610	Csa3G895650	Csa6G006840
Csa2G162660	Csa7G257840	Csa3G895650	Csa3G623980
Csa2G162660	Csa7G268530	Csa3G895650	Csa1G000550
Csa2G162660	Csa7G278200	Csa3G895650	Csa1G000610
Csa2G162660	Csa7G279240	Csa3G895650	Csa2G379090
Csa2G162660	Csa7G281360	Csa3G895650	Csa2G382740
Csa2G162660	Csa3G775270	Csa3G895650	Csa5G175810
Csa2G162660	Csa3G775290	Csa3G895650	Csa3G732570
Csa2G162660	Csa3G778360	Csa3G895650	Csa3G730900
Csa2G162660	Csa3G778370	Csa3G895650	Csa3G728110
Csa2G162660	Csa3G778990	Csa3G895650	Csa1G569180
Csa2G162660	Csa3G782760	Csa3G895650	Csa5G072680
Csa2G162660	Csa3G783840	Csa3G895650	Csa6G421780
Csa2G162660	Csa3G791540	Csa3G895650	Csa3G651770
Csa2G162660	Csa3G798110	Csa3G895650	Csa1G015710
Csa2G162660	Csa3G799120	Csa3G895650	Csa7G407780
Csa2G162660	Csa3G800710	Csa3G895650	Csa1G448950
Csa2G162660	Csa3G806230	Csa3G895650	Csa1G084290
Csa2G162660	Csa3G808370	Csa3G895650	Csa1G084280
Csa2G162660	Csa3G808390	Csa3G895650	Csa2G338890
Csa2G162660	Csa3G809400	Csa3G895650	Csa2G271460
Csa2G162660	Csa3G810540	Csa3G895650	Csa1G226430
Csa2G162660	Csa3G209460	Csa3G895650	Csa2G296060
Csa2G162660	Csa3G207390	Csa3G895650	Csa3G172950
Csa2G162660	Csa3G205300	Csa3G895650	Csa3G154390
Csa2G162660	Csa3G202730	Csa3G895650	Csa3G146600
Csa2G162660	Csa3G200710	Csa3G895650	Csa3G146580
Csa2G162660	Csa3G200700	Csa3G895650	Csa3G146320
Csa2G162660	Csa3G199550	Csa3G895650	Csa3G131940
Csa2G162660	Csa7G133920	Csa3G895650	Csa3G129720
Csa2G162660	Csa5G381780	Csa3G895650	Csa3G117430
Csa2G162660	Csa3G686210	Csa3G895650	Csa4G664580
Csa2G162660	Csa3G686710	Csa3G895650	Csa5G495970
Csa2G162660	Csa3G687750	Csa3G895650	Csa5G579050
Csa2G162660	Csa3G689780	Csa3G895650	Csa5G606610
Csa2G162660	Csa1G226430	Csa3G895650	Csa5G606450
Csa2G162660	Csa2G302300	Csa3G895650	Csa5G606270
Csa2G162660	Csa2G302170	Csa3G895650	Csa5G604410
Csa2G162660	Csa2G301490	Csa3G895650	Csa4G000560
Csa2G162660	Csa2G299880	Csa3G895650	Csa5G162040
Csa2G162660	Csa2G297760	Csa3G895650	Csa5G115160
Csa2G162660	Csa2G297240	Csa3G895650	Csa5G623740
Csa2G162660	Csa2G296010	Csa3G895650	Csa5G615290
Csa2G162660	Csa2G296000	Csa3G895650	Csa1G175730
Csa2G162660	Csa2G295420	Csa3G895650	Csa1G166250
Csa2G162660	Csa3G180240	Csa3G895650	Csa3G002450

Csa2G162660	Csa3G180220	Csa3G895650	Csa5G440130
Csa2G162660	Csa3G178550	Csa3G895650	Csa3G822260
Csa2G162660	Csa3G177990	Csa3G895650	Csa3G822470
Csa2G162660	Csa3G177950	Csa3G895650	Csa3G842030
Csa2G162660	Csa3G177390	Csa3G895650	Csa6G092550
Csa2G162660	Csa3G176320	Csa3G895650	Csa1G265120
Csa2G162660	Csa3G174570	Csa3G895650	Csa4G642520
Csa2G162660	Csa3G171220	Csa3G895650	Csa1G629200
Csa2G162660	Csa3G171210	Csa3G895650	Csa7G448820
Csa2G162660	Csa3G171150	Csa3G895650	Csa6G074600
Csa2G162660	Csa3G167360	Csa3G895650	Csa6G024410
Csa2G162660	Csa3G166300	Csa3G895650	Csa5G199310
Csa2G162660	Csa3G166280	Csa3G895650	Csa6G303790
Csa2G162660	Csa3G164510	Csa3G895650	Csa2G190750
Csa2G162660	Csa3G159450	Csa3G895650	Csa2G199910
Csa2G162660	Csa3G154400	Csa3G895650	Csa7G373470
Csa2G162660	Csa3G154290	Csa3G895650	Csa2G350260
Csa2G162660	Csa3G153700	Csa3G895650	Csa2G352400
Csa2G162660	Csa3G151400	Csa3G895650	Csa2G354100
Csa2G162660	Csa3G150110	Csa6G405920	Csa2G431130
Csa2G162660	Csa3G150080	Csa6G405920	Csa7G393970
Csa2G162660	Csa3G150050	Csa6G405920	Csa5G627080
Csa2G162660	Csa3G149960	Csa6G405920	Csa1G229510
Csa2G162660	Csa3G146670	Csa6G405920	Csa3G043910
Csa2G162660	Csa3G146610	Csa6G405920	Csa6G505840
Csa2G162660	Csa3G146560	Csa6G405920	Csa6G505850
Csa2G162660	Csa3G146510	Csa6G405920	Csa6G514850
Csa2G162660	Csa3G146320	Csa6G405920	Csa6G526290
Csa2G162660	Csa3G146310	Csa6G405920	Csa3G391900
Csa2G162660	Csa3G145780	Csa6G405920	Csa4G618420
Csa2G162660	Csa3G144740	Csa6G405920	Csa1G294600
Csa2G162660	Csa3G144220	Csa6G405920	Csa4G291360
Csa2G162660	Csa3G143610	Csa6G405920	Csa2G419960
Csa2G162660	Csa3G143540	Csa6G405920	Csa6G358710
Csa2G162660	Csa3G142980	Csa6G405920	Csa7G336510
Csa2G162660	Csa3G142400	Csa6G405920	Csa7G291110
Csa2G162660	Csa3G141850	Csa6G405920	Csa7G290450
Csa2G162660	Csa3G141820	Csa6G405920	Csa6G450960
Csa2G162660	Csa3G135660	Csa6G405920	Csa7G388430
Csa2G162660	Csa3G135630	Csa6G405920	Csa1G062880
Csa2G162660	Csa3G135110	Csa6G405920	Csa2G403730
Csa2G162660	Csa3G135040	Csa6G405920	Csa2G401410
Csa2G162660	Csa3G135030	Csa6G405920	Csa2G036080
Csa2G162660	Csa3G134910	Csa6G405920	Csa2G035510
Csa2G162660	Csa3G134880	Csa6G405920	Csa2G030060
Csa2G162660	Csa3G134840	Csa6G405920	Csa1G422430
Csa2G162660	Csa3G134820	Csa6G405920	Csa4G010960
Csa2G162660	Csa3G134630	Csa6G405920	Csa7G021910

Csa2G162660	Csa3G134590	Csa6G405920	Csa7G023930
Csa2G162660	Csa3G134510	Csa6G405920	Csa7G023960
Csa2G162660	Csa3G133960	Csa6G405920	Csa6G490960
Csa2G162660	Csa3G133290	Csa6G405920	Csa6G499140
Csa2G162660	Csa3G133260	Csa6G405920	Csa7G432640
Csa2G162660	Csa3G133180	Csa6G405920	Csa7G431360
Csa2G162660	Csa3G133110	Csa6G405920	Csa4G046810
Csa2G162660	Csa3G133100	Csa6G405920	Csa2G249850
Csa2G162660	Csa3G129750	Csa6G405920	Csa1G024860
Csa2G162660	Csa3G129690	Csa6G405920	Csa1G031200
Csa2G162660	Csa3G129630	Csa6G405920	Csa1G038940
Csa2G162660	Csa3G129620	Csa6G405920	Csa4G052640
Csa2G162660	Csa3G129490	Csa6G405920	Csa2G000210
Csa2G162660	Csa3G128900	Csa6G405920	Csa6G105670
Csa2G162660	Csa3G128830	Csa6G405920	Csa6G446340
Csa2G162660	Csa3G127170	Csa6G405920	Csa6G448130
Csa2G162660	Csa3G127080	Csa6G405920	Csa1G072450
Csa2G162660	Csa3G127040	Csa6G405920	Csa7G420740
Csa2G162660	Csa3G127020	Csa6G405920	Csa6G006840
Csa2G162660	Csa3G126860	Csa6G405920	Csa6G008730
Csa2G162660	Csa3G126220	Csa6G405920	Csa6G406540
Csa2G162660	Csa3G126210	Csa6G405920	Csa6G405940
Csa2G162660	Csa3G126180	Csa6G405920	Csa6G190250
Csa2G162660	Csa3G126100	Csa6G405920	Csa3G623980
Csa2G162660	Csa3G125000	Csa6G405920	Csa3G611350
Csa2G162660	Csa3G124810	Csa6G405920	Csa5G218750
Csa2G162660	Csa3G123190	Csa6G405920	Csa7G041300
Csa2G162660	Csa3G123170	Csa6G405920	Csa3G646580
Csa2G162660	Csa3G122450	Csa6G405920	Csa3G598390
Csa2G162660	Csa3G120500	Csa6G405920	Csa1G000610
Csa2G162660	Csa3G119850	Csa6G405920	Csa1G002680
Csa2G162660	Csa3G119830	Csa6G405920	Csa2G379240
Csa2G162660	Csa3G119580	Csa6G405920	Csa2G382740
Csa2G162660	Csa3G119560	Csa6G405920	Csa5G175810
Csa2G162660	Csa3G119420	Csa6G405920	Csa1G538820
Csa2G162660	Csa3G118730	Csa6G405920	Csa4G457190
Csa2G162660	Csa3G118200	Csa6G405920	Csa5G315360
Csa2G162660	Csa3G118150	Csa6G405920	Csa3G701570
Csa2G162660	Csa3G116800	Csa6G405920	Csa2G075400
Csa2G162660	Csa3G116760	Csa6G405920	Csa4G064100
Csa2G162660	Csa3G116740	Csa6G405920	Csa4G056630
Csa2G162660	Csa3G113280	Csa6G405920	Csa1G569180
Csa2G162660	Csa6G238650	Csa6G405920	Csa6G411180
Csa2G162660	Csa6G239150	Csa6G405920	Csa6G425750
Csa2G162660	Csa5G056620	Csa6G405920	Csa2G326470
Csa2G162660	Csa1G250160	Csa6G405920	Csa3G651770
Csa2G162660	Csa2G108600	Csa6G405920	Csa1G132060
Csa2G162660	Csa2G108610	Csa6G405920	Csa7G407520

Csa2G162660	Csa2G108690	Csa6G405920	Csa1G097710
Csa2G162660	Csa2G115750	Csa6G405920	Csa1G084290
Csa2G162660	Csa2G116270	Csa6G405920	Csa1G084280
Csa2G162660	Csa2G118280	Csa6G405920	Csa2G362410
Csa2G162660	Csa2G119350	Csa6G405920	Csa2G361490
Csa2G162660	Csa2G122000	Csa6G405920	Csa2G359920
Csa2G162660	Csa2G122020	Csa6G405920	Csa7G232500
Csa2G162660	Csa2G123620	Csa6G405920	Csa7G239020
Csa2G162660	Csa2G138190	Csa6G405920	Csa7G278730
Csa2G162660	Csa4G649590	Csa6G405920	Csa3G799650
Csa2G162660	Csa4G649610	Csa6G405920	Csa3G809970
Csa2G162660	Csa4G651780	Csa6G405920	Csa2G295970
Csa2G162660	Csa4G651800	Csa6G405920	Csa3G172990
Csa2G162660	Csa4G651840	Csa6G405920	Csa3G154300
Csa2G162660	Csa4G651870	Csa6G405920	Csa3G149950
Csa2G162660	Csa4G652140	Csa6G405920	Csa3G127820
Csa2G162660	Csa4G652740	Csa6G405920	Csa3G124860
Csa2G162660	Csa4G652820	Csa6G405920	Csa3G121710
Csa2G162660	Csa4G653410	Csa6G405920	Csa3G117430
Csa2G162660	Csa4G653480	Csa6G405920	Csa5G563290
Csa2G162660	Csa4G658500	Csa6G405920	Csa5G614670
Csa2G162660	Csa4G663730	Csa6G405920	Csa5G609640
Csa2G162660	Csa4G664370	Csa6G405920	Csa5G608250
Csa2G162660	Csa4G664380	Csa6G405920	Csa5G606450
Csa2G162660	Csa4G664480	Csa6G405920	Csa5G606320
Csa2G162660	Csa4G664500	Csa6G405920	Csa5G606270
Csa2G162660	Csa4G665130	Csa6G405920	Csa5G604100
Csa2G162660	Csa4G096610	Csa6G405920	Csa5G602190
Csa2G162660	Csa4G090360	Csa6G405920	Csa4G000580
Csa2G162660	Csa4G088740	Csa6G405920	Csa4G646130
Csa2G162660	Csa4G088720	Csa6G405920	Csa4G646370
Csa2G162660	Csa4G083660	Csa6G405920	Csa3G535620
Csa2G162660	Csa4G082310	Csa6G405920	Csa3G516510
Csa2G162660	Csa4G081300	Csa6G405920	Csa5G160150
Csa2G162660	Csa4G374120	Csa6G405920	Csa5G153120
Csa2G162660	Csa4G372620	Csa6G405920	Csa3G743450
Csa2G162660	Csa5G491820	Csa6G405920	Csa3G740080
Csa2G162660	Csa5G494390	Csa6G405920	Csa5G623500
Csa2G162660	Csa5G494420	Csa6G405920	Csa3G912350
Csa2G162660	Csa5G495960	Csa6G405920	Csa2G435430
Csa2G162660	Csa5G496470	Csa6G405920	Csa1G166250
Csa2G162660	Csa5G497020	Csa6G405920	Csa3G038100
Csa2G162660	Csa5G276460	Csa6G405920	Csa3G002830
Csa2G162660	Csa5G272920	Csa6G405920	Csa6G366250
Csa2G162660	Csa5G269890	Csa6G405920	Csa1G701380
Csa2G162660	Csa5G266870	Csa6G405920	Csa1G701390
Csa2G162660	Csa2G083750	Csa6G405920	Csa3G822260
Csa2G162660	Csa2G083760	Csa6G405920	Csa3G829130

Csa2G162660	Csa2G093840	Csa6G405920	Csa3G837640
Csa2G162660	Csa7G101780	Csa6G405920	Csa3G855420
Csa2G162660	Csa7G098760	Csa6G405920	Csa3G872070
Csa2G162660	Csa5G568810	Csa6G405920	Csa1G629200
Csa2G162660	Csa5G569320	Csa6G405920	Csa1G657480
Csa2G162660	Csa5G571440	Csa6G405920	Csa7G446940
Csa2G162660	Csa5G571480	Csa6G405920	Csa7G448730
Csa2G162660	Csa5G576650	Csa6G405920	Csa7G448820
Csa2G162660	Csa5G576660	Csa6G405920	Csa7G448840
Csa2G162660	Csa5G576670	Csa6G405920	Csa7G450720
Csa2G162660	Csa5G576750	Csa6G405920	Csa6G074600
Csa2G162660	Csa5G577360	Csa6G405920	Csa6G062280
Csa2G162660	Csa5G577450	Csa6G405920	Csa6G056470
Csa2G162660	Csa5G577960	Csa6G405920	Csa6G052630
Csa2G162660	Csa5G579060	Csa6G405920	Csa6G042320
Csa2G162660	Csa1G433100	Csa6G405920	Csa6G022370
Csa2G162660	Csa1G433060	Csa6G405920	Csa6G303790
Csa2G162660	Csa1G427530	Csa6G405920	Csa2G169710
Csa2G162660	Csa1G426960	Csa6G405920	Csa2G174120
Csa2G162660	Csa1G425910	Csa6G405920	Csa2G190750
Csa2G162660	Csa5G614680	Csa6G405920	Csa2G191300
Csa2G162660	Csa5G613570	Csa6G405920	Csa2G348170
Csa2G162660	Csa5G613560	Csa6G405920	Csa2G350260
Csa2G162660	Csa5G612900	Csa6G405920	Csa2G354100
Csa2G162660	Csa5G612310	Csa6G405920	Csa1G586820
Csa2G162660	Csa5G611700	Csa6G405920	Csa4G097650
Csa2G162660	Csa5G611040	Csa6G405920	Csa4G111590
Csa2G162660	Csa5G610420	Csa7G447800	Csa1G186660
Csa2G162660	Csa5G610360	Csa7G447800	Csa1G701960
Csa2G162660	Csa5G609810	Csa7G447800	Csa1G701990
Csa2G162660	Csa5G609720	Csa7G447800	Csa6G399730
Csa2G162660	Csa5G609110	Csa7G447800	Csa6G399760
Csa2G162660	Csa5G608590	Csa7G447800	Csa6G401340
Csa2G162660	Csa5G608570	Csa7G447800	Csa6G401370
Csa2G162660	Csa5G608530	Csa7G447800	Csa6G401530
Csa2G162660	Csa5G608470	Csa7G447800	Csa6G403580
Csa2G162660	Csa5G608460	Csa7G447800	Csa6G404140
Csa2G162660	Csa5G608350	Csa7G447800	Csa6G404230
Csa2G162660	Csa5G608250	Csa7G447800	Csa6G404260
Csa2G162660	Csa5G608190	Csa7G447800	Csa5G420310
Csa2G162660	Csa5G608010	Csa7G447800	Csa7G213180
Csa2G162660	Csa5G606780	Csa7G447800	Csa5G517760
Csa2G162660	Csa5G606740	Csa7G447800	Csa5G517800
Csa2G162660	Csa5G606640	Csa7G447800	Csa5G523120
Csa2G162660	Csa5G606600	Csa7G447800	Csa5G523170
Csa2G162660	Csa5G606380	Csa7G447800	Csa5G524700
Csa2G162660	Csa5G606330	Csa7G447800	Csa5G524840
Csa2G162660	Csa5G605730	Csa7G447800	Csa5G533470

Csa2G162660	Csa5G605080	Csa7G447800	Csa3G333840
Csa2G162660	Csa5G604990	Csa7G447800	Csa3G337350
Csa2G162660	Csa5G604970	Csa7G447800	Csa3G342350
Csa2G162660	Csa5G604910	Csa7G447800	Csa3G348940
Csa2G162660	Csa5G604230	Csa7G447800	Csa3G357110
Csa2G162660	Csa5G604080	Csa7G447800	Csa5G353650
Csa2G162660	Csa5G603970	Csa7G447800	Csa5G352640
Csa2G162660	Csa5G603280	Csa7G447800	Csa2G228390
Csa2G162660	Csa5G603260	Csa7G447800	Csa2G231960
Csa2G162660	Csa5G602760	Csa7G447800	Csa2G433360
Csa2G162660	Csa5G602750	Csa7G447800	Csa4G358630
Csa2G162660	Csa5G601550	Csa7G447800	Csa5G011740
Csa2G162660	Csa5G601540	Csa7G447800	Csa4G192180
Csa2G162660	Csa5G600940	Csa7G447800	Csa7G394660
Csa2G162660	Csa5G600920	Csa7G447800	Csa5G091890
Csa2G162660	Csa5G598740	Csa7G447800	Csa1G666960
Csa2G162660	Csa5G598670	Csa7G447800	Csa3G292550
Csa2G162660	Csa5G598060	Csa7G447800	Csa3G285040
Csa2G162660	Csa5G597500	Csa7G447800	Csa3G264750
Csa2G162660	Csa5G593400	Csa7G447800	Csa3G257090
Csa2G162660	Csa1G580210	Csa7G447800	Csa3G253490
Csa2G162660	Csa1G575190	Csa7G447800	Csa3G252450
Csa2G162660	Csa1G575040	Csa7G447800	Csa3G247880
Csa2G162660	Csa1G573590	Csa7G447800	Csa3G238740
Csa2G162660	Csa4G075740	Csa7G447800	Csa3G238210
Csa2G162660	Csa1G690160	Csa7G447800	Csa3G238090
Csa2G162660	Csa1G690230	Csa7G447800	Csa3G236030
Csa2G162660	Csa1G690240	Csa7G447800	Csa3G232440
Csa2G162660	Csa1G690280	Csa7G447800	Csa3G221730
Csa2G162660	Csa1G690300	Csa7G447800	Csa5G466330
Csa2G162660	Csa1G690370	Csa7G447800	Csa5G468980
Csa2G162660	Csa1G681130	Csa7G447800	Csa5G471600
Csa2G162660	Csa1G103270	Csa7G447800	Csa5G407090
Csa2G162660	Csa3G497390	Csa7G447800	Csa1G231010
Csa2G162660	Csa4G000670	Csa7G447800	Csa3G608720
Csa2G162660	Csa4G000620	Csa7G447800	Csa3G457650
Csa2G162660	Csa4G000530	Csa7G447800	Csa3G524590
Csa2G162660	Csa4G646070	Csa7G447800	Csa3G043910
Csa2G162660	Csa4G646130	Csa7G447800	Csa3G060980
Csa2G162660	CsaUNG005730	Csa7G447800	Csa3G063640
Csa2G162660	CsaUNG003730	Csa7G447800	Csa3G073840
Csa2G162660	Csa3G539670	Csa7G447800	Csa3G073910
Csa2G162660	Csa3G509940	Csa7G447800	Csa6G504630
Csa2G162660	Csa3G516540	Csa7G447800	Csa6G504670
Csa2G162660	Csa5G162040	Csa7G447800	Csa6G504690
Csa2G162660	Csa5G162030	Csa7G447800	Csa6G505300
Csa2G162660	Csa5G161970	Csa7G447800	Csa6G505990
Csa2G162660	Csa5G160750	Csa7G447800	Csa6G506010

Csa2G162660	Csa5G160170	Csa7G447800	Csa6G507440
Csa2G162660	Csa5G157240	Csa7G447800	Csa6G509570
Csa2G162660	Csa5G155580	Csa7G447800	Csa6G509600
Csa2G162660	Csa5G154790	Csa7G447800	Csa6G509630
Csa2G162660	Csa5G154780	Csa7G447800	Csa6G510300
Csa2G162660	Csa5G152890	Csa7G447800	Csa6G510960
Csa2G162660	Csa5G152860	Csa7G447800	Csa6G511670
Csa2G162660	Csa5G152800	Csa7G447800	Csa6G511700
Csa2G162660	Csa5G151000	Csa7G447800	Csa6G513680
Csa2G162660	Csa5G150990	Csa7G447800	Csa6G514290
Csa2G162660	Csa5G150970	Csa7G447800	Csa6G514820
Csa2G162660	Csa5G149870	Csa7G447800	Csa6G516660
Csa2G162660	Csa5G148530	Csa7G447800	Csa6G517200
Csa2G162660	Csa3G742880	Csa7G447800	Csa6G517220
Csa2G162660	Csa3G740220	Csa7G447800	Csa6G517410
Csa2G162660	Csa3G740120	Csa7G447800	Csa6G517950
Csa2G162660	Csa3G739560	Csa7G447800	Csa6G517960
Csa2G162660	Csa3G738990	Csa7G447800	Csa6G518110
Csa2G162660	Csa3G738980	Csa7G447800	Csa6G518350
Csa2G162660	Csa3G736900	Csa7G447800	Csa6G518930
Csa2G162660	Csa3G736820	Csa7G447800	Csa6G519620
Csa2G162660	Csa3G736800	Csa7G447800	Csa6G520350
Csa2G162660	Csa3G736770	Csa7G447800	Csa6G520410
Csa2G162660	Csa3G736630	Csa7G447800	Csa6G521610
Csa2G162660	Csa3G735050	Csa7G447800	Csa6G523350
Csa2G162660	Csa5G624460	Csa7G447800	Csa6G525680
Csa2G162660	Csa5G623870	Csa7G447800	Csa6G525700
Csa2G162660	Csa5G623840	Csa7G447800	Csa6G538600
Csa2G162660	Csa5G623830	Csa7G447800	Csa6G538610
Csa2G162660	Csa5G623790	Csa7G447800	Csa6G538700
Csa2G162660	Csa5G623720	Csa7G447800	Csa3G214070
Csa2G162660	Csa5G623540	Csa7G447800	Csa3G214020
Csa2G162660	Csa5G623520	Csa7G447800	Csa3G212490
Csa2G162660	Csa5G623460	Csa7G447800	Csa7G429570
Csa2G162660	Csa5G622830	Csa7G447800	Csa1G561370
Csa2G162660	Csa5G622770	Csa7G447800	Csa1G561420
Csa2G162660	Csa5G622750	Csa7G447800	Csa1G568480
Csa2G162660	Csa5G622610	Csa7G447800	Csa1G569100
Csa2G162660	Csa5G622560	Csa7G447800	Csa1G569140
Csa2G162660	Csa5G622550	Csa7G447800	Csa6G289720
Csa2G162660	Csa5G622500	Csa7G447800	Csa6G290830
Csa2G162660	Csa5G622440	Csa7G447800	Csa6G290870
Csa2G162660	Csa5G616350	Csa7G447800	Csa3G384790
Csa2G162660	Csa3G875960	Csa7G447800	Csa3G383780
Csa2G162660	Csa3G878730	Csa7G447800	Csa3G383760
Csa2G162660	Csa3G878780	Csa7G447800	Csa3G585900
Csa2G162660	Csa3G878800	Csa7G447800	Csa3G588470
Csa2G162660	Csa3G878860	Csa7G447800	Csa1G435790

Csa2G162660	Csa3G878880	Csa7G447800	Csa1G435750
Csa2G162660	Csa3G878940	Csa7G447800	Csa1G616850
Csa2G162660	Csa3G880030	Csa7G447800	Csa6G365140
Csa2G162660	Csa3G880550	Csa7G447800	Csa6G365160
Csa2G162660	Csa3G881640	Csa7G447800	Csa7G145970
Csa2G162660	Csa3G881650	Csa7G447800	Csa4G627100
Csa2G162660	Csa3G881790	Csa7G447800	Csa4G626100
Csa2G162660	Csa3G881870	Csa7G447800	Csa4G622840
Csa2G162660	Csa3G881910	Csa7G447800	Csa4G621210
Csa2G162660	Csa3G883020	Csa7G447800	Csa4G618500
Csa2G162660	Csa3G889780	Csa7G447800	Csa4G608170
Csa2G162660	Csa3G889870	Csa7G447800	Csa3G081930
Csa2G162660	Csa3G889920	Csa7G447800	Csa3G077620
Csa2G162660	Csa3G889960	Csa7G447800	Csa3G076560
Csa2G162660	Csa3G890120	Csa7G447800	Csa3G076550
Csa2G162660	Csa3G891660	Csa7G447800	Csa3G076010
Csa2G162660	Csa3G893370	Csa7G447800	Csa5G652280
Csa2G162660	Csa3G893440	Csa7G447800	Csa5G652210
Csa2G162660	Csa3G894460	Csa7G447800	Csa3G483750
Csa2G162660	Csa3G895100	Csa7G447800	Csa1G305770
Csa2G162660	Csa3G895680	Csa7G447800	Csa1G295150
Csa2G162660	Csa3G895690	Csa7G447800	Csa4G290220
Csa2G162660	Csa3G895870	Csa7G447800	Csa4G290750
Csa2G162660	Csa3G901000	Csa7G447800	Csa4G290830
Csa2G162660	Csa3G901170	Csa7G447800	Csa4G293110
Csa2G162660	Csa3G901190	Csa7G447800	Csa4G294380
Csa2G162660	Csa3G902270	Csa7G447800	Csa4G295470
Csa2G162660	Csa3G902310	Csa7G447800	Csa4G296180
Csa2G162660	Csa3G902390	Csa7G447800	Csa4G297510
Csa2G162660	Csa3G903520	Csa7G447800	Csa4G303070
Csa2G162660	Csa3G904080	Csa7G447800	Csa4G303120
Csa2G162660	Csa3G910720	Csa7G447800	Csa4G309150
Csa2G162660	Csa3G911260	Csa7G447800	Csa5G308800
Csa2G162660	Csa3G912300	Csa7G447800	Csa5G310810
Csa2G162660	Csa3G912960	Csa7G447800	Csa4G430820
Csa2G162660	Csa3G912970	Csa7G447800	Csa4G429320
Csa2G162660	Csa3G914060	Csa7G447800	Csa6G318660
Csa2G162660	Csa3G915100	Csa7G447800	Csa6G318140
Csa2G162660	Csa3G915180	Csa7G447800	Csa3G423790
Csa2G162660	Csa6G308410	Csa7G447800	Csa2G416820
Csa2G162660	Csa2G324430	Csa7G447800	Csa2G419960
Csa2G162660	Csa2G439190	Csa7G447800	Csa2G421010
Csa2G162660	Csa2G437120	Csa7G447800	Csa2G423600
Csa2G162660	Csa2G435540	Csa7G447800	Csa2G427880
Csa2G162660	Csa1G173210	Csa7G447800	Csa3G104900
Csa2G162660	Csa1G171050	Csa7G447800	Csa3G110020
Csa2G162660	Csa1G169420	Csa7G447800	Csa5G593340
Csa2G162660	Csa1G166820	Csa7G447800	Csa5G593330

Csa2G162660	Csa1G166250	Csa7G447800	Csa5G591760
Csa2G162660	Csa3G038170	Csa7G447800	Csa5G590000
Csa2G162660	Csa3G036560	Csa7G447800	Csa5G589920
Csa2G162660	Csa3G036430	Csa7G447800	Csa5G589890
Csa2G162660	Csa3G035840	Csa7G447800	Csa5G589390
Csa2G162660	Csa3G021150	Csa7G447800	Csa5G587190
Csa2G162660	Csa3G017190	Csa7G447800	Csa5G586030
Csa2G162660	Csa3G017120	Csa7G447800	Csa5G585990
Csa2G162660	Csa3G017010	Csa7G447800	Csa5G585450
Csa2G162660	Csa3G011800	Csa7G447800	Csa6G338050
Csa2G162660	Csa3G011750	Csa7G447800	Csa6G349830
Csa2G162660	Csa3G011080	Csa7G447800	Csa6G349850
Csa2G162660	Csa3G009520	Csa7G447800	Csa6G355440
Csa2G162660	Csa3G009510	Csa7G447800	Csa6G356490
Csa2G162660	Csa3G009460	Csa7G447800	Csa6G358130
Csa2G162660	Csa3G008910	Csa7G447800	Csa7G336510
Csa2G162660	Csa3G008330	Csa7G447800	Csa7G328310
Csa2G162660	Csa3G006630	Csa7G447800	Csa7G307400
Csa2G162660	Csa3G006600	Csa7G447800	Csa1G423270
Csa2G162660	Csa3G003480	Csa7G447800	Csa1G423280
Csa2G162660	Csa3G002590	Csa7G447800	Csa1G423290
Csa2G162660	Csa3G002500	Csa7G447800	Csa1G423340
Csa2G162660	Csa3G002490	Csa7G447800	Csa6G450420
Csa2G162660	Csa3G002370	Csa7G447800	Csa6G451500
Csa2G162660	Csa3G001750	Csa7G447800	Csa6G452060
Csa2G162660	Csa3G001720	Csa7G447800	Csa6G452640
Csa2G162660	Csa3G000120	Csa7G447800	Csa6G453770
Csa2G162660	Csa6G309980	Csa7G447800	Csa6G453800
Csa2G162660	Csa6G014890	Csa7G447800	Csa6G454480
Csa2G162660	Csa6G014830	Csa7G447800	Csa7G387730
Csa2G162660	Csa6G014820	Csa7G447800	Csa7G388310
Csa2G162660	Csa6G014800	Csa7G447800	Csa7G388370
Csa2G162660	Csa6G014770	Csa7G447800	Csa7G390130
Csa2G162660	Csa6G014570	Csa7G447800	Csa7G391260
Csa2G162660	Csa6G014480	Csa7G447800	Csa2G234550
Csa2G162660	CsaUNG008810	Csa7G447800	Csa2G234580
Csa2G162660	CsaUNG008850	Csa7G447800	Csa1G059760
Csa2G162660	Csa6G365720	Csa7G447800	Csa1G064710
Csa2G162660	Csa6G366280	Csa7G447800	Csa1G064750
Csa2G162660	Csa6G366290	Csa7G447800	Csa1G065380
Csa2G162660	Csa6G366360	Csa7G447800	Csa1G065390
Csa2G162660	Csa6G366400	Csa7G447800	Csa1G065960
Csa2G162660	Csa6G366430	Csa7G447800	Csa6G427970
Csa2G162660	Csa6G367080	Csa7G447800	Csa6G430650
Csa2G162660	Csa6G381850	Csa7G447800	Csa6G430710
Csa2G162660	Csa4G182250	Csa7G447800	Csa2G408410
Csa2G162660	Csa4G182220	Csa7G447800	Csa2G406770
Csa2G162660	Csa4G179700	Csa7G447800	Csa2G406720

Csa2G162660	Csa4G179170	Csa7G447800	Csa2G406700
Csa2G162660	Csa4G179150	Csa7G447800	Csa2G406060
Csa2G162660	Csa1G699630	Csa7G447800	Csa2G405030
Csa2G162660	Csa1G700690	Csa7G447800	Csa2G404790
Csa2G162660	Csa1G701220	Csa7G447800	Csa2G404760
Csa2G162660	Csa1G616250	Csa7G447800	Csa2G403140
Csa2G162660	Csa7G195270	Csa7G447800	Csa2G401340
Csa2G162660	Csa7G188690	Csa7G447800	Csa2G061530
Csa2G162660	Csa4G188400	Csa7G447800	Csa2G060370
Csa2G162660	Csa4G188940	Csa7G447800	Csa2G049330
Csa2G162660	Csa5G440110	Csa7G447800	Csa2G036620
Csa2G162660	Csa5G441650	Csa7G447800	Csa2G036040
Csa2G162660	Csa3G812230	Csa7G447800	Csa2G035510
Csa2G162660	Csa3G816030	Csa7G447800	Csa2G035380
Csa2G162660	Csa3G816080	Csa7G447800	Csa2G031190
Csa2G162660	Csa3G819910	Csa7G447800	Csa2G022830
Csa2G162660	Csa3G821570	Csa7G447800	Csa2G022790
Csa2G162660	Csa3G821590	Csa7G447800	Csa2G022250
Csa2G162660	Csa3G822250	Csa7G447800	Csa2G021740
Csa2G162660	Csa3G822300	Csa7G447800	Csa2G020990
Csa2G162660	Csa3G822380	Csa7G447800	Csa2G020920
Csa2G162660	Csa3G822390	Csa7G447800	Csa2G020850
Csa2G162660	Csa3G822470	Csa7G447800	Csa1G422990
Csa2G162660	Csa3G824200	Csa7G447800	Csa1G423000
Csa2G162660	Csa3G824910	Csa7G447800	Csa1G256190
Csa2G162660	Csa3G825010	Csa7G447800	Csa4G026890
Csa2G162660	Csa3G826680	Csa7G447800	Csa4G026870
Csa2G162660	Csa3G827200	Csa7G447800	Csa4G022910
Csa2G162660	Csa3G827290	Csa7G447800	Csa4G022870
Csa2G162660	Csa3G827300	Csa7G447800	Csa4G022860
Csa2G162660	Csa3G829040	Csa7G447800	Csa4G022350
Csa2G162660	Csa3G829120	Csa7G447800	Csa4G016460
Csa2G162660	Csa3G829150	Csa7G447800	Csa4G015820
Csa2G162660	Csa3G836500	Csa7G447800	Csa4G012480
Csa2G162660	Csa3G838700	Csa7G447800	Csa4G011600
Csa2G162660	Csa3G838750	Csa7G447800	Csa4G009890
Csa2G162660	Csa3G839820	Csa7G447800	Csa4G008800
Csa2G162660	Csa3G840960	Csa7G447800	Csa4G006400
Csa2G162660	Csa3G842050	Csa7G447800	Csa4G006310
Csa2G162660	Csa3G842110	Csa7G447800	Csa4G006280
Csa2G162660	Csa3G842660	Csa7G447800	Csa4G006260
Csa2G162660	Csa3G842740	Csa7G447800	Csa4G006250
Csa2G162660	Csa3G843820	Csa7G447800	Csa4G006190
Csa2G162660	Csa3G844880	Csa7G447800	Csa4G006180
Csa2G162660	Csa3G845460	Csa7G447800	Csa4G004970
Csa2G162660	Csa3G846010	Csa7G447800	Csa4G004940
Csa2G162660	Csa3G847610	Csa7G447800	Csa4G001940
Csa2G162660	Csa3G848160	Csa7G447800	Csa4G001810

Csa2G162660	Csa3G848220	Csa7G447800	Csa4G001590
Csa2G162660	Csa3G848290	Csa7G447800	Csa4G001540
Csa2G162660	Csa3G848830	Csa7G447800	Csa1G044830
Csa2G162660	Csa3G848850	Csa7G447800	Csa1G045500
Csa2G162660	Csa3G850530	Csa7G447800	Csa1G045570
Csa2G162660	Csa3G851790	Csa7G447800	Csa1G045890
Csa2G162660	Csa3G852580	Csa7G447800	Csa1G045940
Csa2G162660	Csa3G852630	Csa7G447800	Csa1G046030
Csa2G162660	Csa3G854180	Csa7G447800	Csa7G018750
Csa2G162660	Csa3G854260	Csa7G447800	Csa7G021920
Csa2G162660	Csa3G855310	Csa7G447800	Csa7G023940
Csa2G162660	Csa3G855350	Csa7G447800	Csa7G024670
Csa2G162660	Csa3G855380	Csa7G447800	Csa7G029930
Csa2G162660	Csa3G855410	Csa7G447800	Csa7G030500
Csa2G162660	Csa3G859650	Csa7G447800	Csa7G032280
Csa2G162660	Csa3G866530	Csa7G447800	Csa6G483280
Csa2G162660	Csa3G872030	Csa7G447800	Csa6G483290
Csa2G162660	Csa3G872080	Csa7G447800	Csa6G483370
Csa2G162660	Csa3G873270	Csa7G447800	Csa6G483450
Csa2G162660	Csa3G874390	Csa7G447800	Csa6G484020
Csa2G162660	Csa7G058560	Csa7G447800	Csa6G485170
Csa2G162660	Csa7G058530	Csa7G447800	Csa6G486990
Csa2G162660	Csa7G056460	Csa7G447800	Csa6G487690
Csa2G162660	Csa7G051410	Csa7G447800	Csa6G487740
Csa2G162660	Csa7G051370	Csa7G447800	Csa6G490240
Csa2G162660	Csa7G050800	Csa7G447800	Csa6G492280
Csa2G162660	Csa6G086150	Csa7G447800	Csa6G493830
Csa2G162660	Csa6G086160	Csa7G447800	Csa6G496410
Csa2G162660	Csa6G088010	Csa7G447800	Csa6G497210
Csa2G162660	Csa6G088130	Csa7G447800	Csa6G497350
Csa2G162660	Csa6G089270	Csa7G447800	Csa6G498930
Csa2G162660	Csa6G091290	Csa7G447800	Csa6G499110
Csa2G162660	Csa6G095330	Csa7G447800	Csa6G499210
Csa2G162660	Csa6G095890	Csa7G447800	Csa6G500500
Csa2G162660	Csa1G264000	Csa7G447800	Csa7G433290
Csa2G162660	Csa1G267270	Csa7G447800	Csa7G432640
Csa2G162660	Csa1G268310	Csa7G447800	Csa7G432470
Csa2G162660	Csa1G269350	Csa7G447800	Csa7G432460
Csa2G162660	Csa1G269850	Csa7G447800	Csa7G432000
Csa2G162660	Csa4G639770	Csa7G447800	Csa7G431970
Csa2G162660	Csa4G639850	Csa7G447800	Csa7G431440
Csa2G162660	Csa4G639870	Csa7G447800	Csa7G431340
Csa2G162660	Csa4G641650	Csa7G447800	Csa4G046670
Csa2G162660	Csa4G641680	Csa7G447800	Csa4G047370
Csa2G162660	Csa4G641710	Csa7G447800	Csa4G047930
Csa2G162660	Csa4G641750	Csa7G447800	Csa4G050840
Csa2G162660	Csa4G642480	Csa7G447800	Csa4G051370
Csa2G162660	Csa4G642540	Csa7G447800	Csa4G051390

Csa2G162660	Csa4G643080	Csa7G447800	Csa3G183900
Csa2G162660	Csa4G645890	Csa7G447800	Csa3G185130
Csa2G162660	Csa4G645920	Csa7G447800	Csa3G186680
Csa2G162660	Csa4G646020	Csa7G447800	Csa3G199020
Csa2G162660	Csa4G646060	Csa7G447800	Csa2G252070
Csa2G162660	Csa4G495220	Csa7G447800	Csa2G251480
Csa2G162660	Csa4G496760	Csa7G447800	Csa2G250940
Csa2G162660	Csa4G499830	Csa7G447800	Csa2G250430
Csa2G162660	Csa1G629050	Csa7G447800	Csa2G248740
Csa2G162660	Csa1G629070	Csa7G447800	Csa2G248710
Csa2G162660	Csa1G629730	Csa7G447800	Csa2G248700
Csa2G162660	Csa1G630300	Csa7G447800	Csa2G248640
Csa2G162660	Csa1G633410	Csa7G447800	Csa2G245480
Csa2G162660	Csa1G638460	Csa7G447800	Csa2G245430
Csa2G162660	Csa1G641020	Csa7G447800	Csa2G238780
Csa2G162660	Csa1G652290	Csa7G447800	Csa2G237150
Csa2G162660	Csa1G656460	Csa7G447800	Csa1G024160
Csa2G162660	Csa4G563700	Csa7G447800	Csa1G024920
Csa2G162660	Csa4G552160	Csa7G447800	Csa1G024930
Csa2G162660	Csa4G419580	Csa7G447800	Csa1G024940
Csa2G162660	Csa7G446670	Csa7G447800	Csa1G025070
Csa2G162660	Csa7G446860	Csa7G447800	Csa1G029620
Csa2G162660	Csa7G446900	Csa7G447800	Csa1G031890
Csa2G162660	Csa7G446920	Csa7G447800	Csa1G032470
Csa2G162660	Csa7G446970	Csa7G447800	Csa1G033040
Csa2G162660	Csa7G447060	Csa7G447800	Csa1G033200
Csa2G162660	Csa7G447990	Csa7G447800	Csa1G038930
Csa2G162660	Csa7G448020	Csa7G447800	Csa3G110640
Csa2G162660	Csa7G448810	Csa7G447800	Csa3G110660
Csa2G162660	Csa7G448860	Csa7G447800	Csa3G111240
Csa2G162660	Csa7G450500	Csa7G447800	Csa3G112780
Csa2G162660	Csa7G450490	Csa7G447800	Csa4G055370
Csa2G162660	Csa7G450630	Csa7G447800	Csa4G055320
Csa2G162660	Csa7G450750	Csa7G447800	Csa4G052640
Csa2G162660	Csa7G451300	Csa7G447800	Csa5G172860
Csa2G162660	Csa7G451350	Csa7G447800	Csa5G171700
Csa2G162660	Csa7G451390	Csa7G447800	Csa2G005920
Csa2G162660	Csa7G452090	Csa7G447800	Csa2G005360
Csa2G162660	Csa7G452290	Csa7G447800	Csa2G004740
Csa2G162660	Csa6G081510	Csa7G447800	Csa2G000800
Csa2G162660	Csa6G080320	Csa7G447800	Csa2G000660
Csa2G162660	Csa6G079790	Csa7G447800	Csa2G000440
Csa2G162660	Csa6G079780	Csa7G447800	Csa1G524640
Csa2G162660	Csa6G078680	Csa7G447800	Csa1G523110
Csa2G162660	Csa6G078650	Csa7G447800	Csa1G523050
Csa2G162660	Csa6G078630	Csa7G447800	Csa6G139250
Csa2G162660	Csa6G078510	Csa7G447800	Csa6G137590
Csa2G162660	Csa6G076840	Csa7G447800	Csa6G134390

Csa2G162660	Csa6G076820	Csa7G447800	Csa6G133680
Csa2G162660	Csa6G076800	Csa7G447800	Csa6G127430
Csa2G162660	Csa6G076750	Csa7G447800	Csa6G127330
Csa2G162660	Csa6G076720	Csa7G447800	Csa6G126260
Csa2G162660	Csa6G075120	Csa7G447800	Csa6G118340
Csa2G162660	Csa6G074550	Csa7G447800	Csa6G115610
Csa2G162660	Csa6G067420	Csa7G447800	Csa6G109800
Csa2G162660	Csa6G062270	Csa7G447800	Csa6G109670
Csa2G162660	Csa6G057150	Csa7G447800	Csa6G109660
Csa2G162660	Csa6G056510	Csa7G447800	Csa6G108590
Csa2G162660	Csa6G056490	Csa7G447800	Csa6G108560
Csa2G162660	Csa6G052770	Csa7G447800	Csa6G104610
Csa2G162660	Csa6G052670	Csa7G447800	Csa6G104080
Csa2G162660	Csa6G052070	Csa7G447800	Csa1G042520
Csa2G162660	Csa6G051520	Csa7G447800	Csa1G042850
Csa2G162660	Csa6G046410	Csa7G447800	Csa1G042890
Csa2G162660	Csa6G042460	Csa7G447800	Csa1G042970
Csa2G162660	Csa6G042440	Csa7G447800	Csa4G627820
Csa2G162660	Csa6G042370	Csa7G447800	Csa4G637130
Csa2G162660	Csa6G042360	Csa7G447800	Csa4G637730
Csa2G162660	Csa6G040560	Csa7G447800	Csa4G639130
Csa2G162660	Csa6G030440	Csa7G447800	Csa4G639740
Csa2G162660	Csa6G022340	Csa7G447800	Csa6G445020
Csa2G162660	Csa6G022330	Csa7G447800	Csa6G445040
Csa2G162660	Csa6G022310	Csa7G447800	Csa6G445070
Csa2G162660	Csa6G018610	Csa7G447800	Csa6G445180
Csa2G162660	Csa6G017090	Csa7G447800	Csa6G446440
Csa2G162660	Csa6G017060	Csa7G447800	Csa6G446460
Csa2G162660	Csa6G017030	Csa7G447800	Csa4G361880
Csa2G162660	Csa6G016970	Csa7G447800	Csa4G362910
Csa2G162660	Csa6G016930	Csa7G447800	Csa4G364040
Csa2G162660	Csa6G016920	Csa7G447800	Csa1G071210
Csa2G162660	Csa2G292810	Csa7G447800	Csa1G071260
Csa2G162660	Csa2G292780	Csa7G447800	Csa1G071270
Csa2G162660	Csa2G292200	Csa7G447800	Csa1G071940
Csa2G162660	Csa2G292190	Csa7G447800	Csa1G073860
Csa2G162660	Csa2G287110	Csa7G447800	Csa1G074990
Csa2G162660	Csa2G286460	Csa7G447800	Csa1G077220
Csa2G162660	Csa2G286470	Csa7G447800	Csa1G077760
Csa2G162660	Csa2G277000	Csa7G447800	Csa7G428210
Csa2G162660	Csa2G277600	Csa7G447800	Csa7G428180
Csa2G162660	Csa2G277610	Csa7G447800	Csa7G428120
Csa2G162660	Csa2G277630	Csa7G447800	Csa7G426560
Csa2G162660	Csa5G211540	Csa7G447800	Csa7G420170
Csa2G162660	Csa5G210010	Csa7G447800	Csa7G419570
Csa2G162660	Csa5G203410	Csa7G447800	Csa7G414510
Csa2G162660	Csa5G203390	Csa7G447800	Csa5G169080
Csa2G162660	Csa5G201320	Csa7G447800	Csa5G168980

Csa2G162660	Csa5G199810	Csa7G447800	Csa5G168790
Csa2G162660	Csa5G198740	Csa7G447800	Csa5G167210
Csa2G162660	Csa7G016600	Csa7G447800	Csa4G312850
Csa2G162660	Csa7G012400	Csa7G447800	Csa4G314460
Csa2G162660	Csa4G370550	Csa7G447800	Csa4G315020
Csa2G162660	Csa4G370570	Csa7G447800	Csa4G329560
Csa2G162660	Csa5G291680	Csa7G447800	Csa4G337340
Csa2G162660	Csa5G118170	Csa7G447800	Csa2G006060
Csa2G162660	Csa5G118180	Csa7G447800	Csa2G007960
Csa2G162660	Csa5G129320	Csa7G447800	Csa2G008080
Csa2G162660	Csa5G129340	Csa7G447800	Csa2G009290
Csa2G162660	Csa4G159320	Csa7G447800	Csa2G009610
Csa2G162660	Csa4G164350	Csa7G447800	Csa2G009620
Csa2G162660	Csa4G165910	Csa7G447800	Csa2G011490
Csa2G162660	Csa3G305660	Csa7G447800	Csa2G012110
Csa2G162660	Csa5G114620	Csa7G447800	Csa1G058140
Csa2G162660	Csa5G114570	Csa7G447800	Csa1G058080
Csa2G162660	Csa7G004050	Csa7G447800	Csa1G051760
Csa2G162660	Csa7G004110	Csa7G447800	Csa1G051660
Csa2G162660	Csa7G004120	Csa7G447800	Csa1G051650
Csa2G162660	Csa7G004710	Csa7G447800	Csa6G000100
Csa2G162660	Csa3G751430	Csa7G447800	Csa6G001720
Csa2G162660	Csa3G750380	Csa7G447800	Csa6G004520
Csa2G162660	Csa3G750370	Csa7G447800	Csa6G004600
Csa2G162660	Csa3G748780	Csa7G447800	Csa6G006710
Csa2G162660	Csa3G748240	Csa7G447800	Csa6G006790
Csa2G162660	Csa3G748200	Csa7G447800	Csa6G006840
Csa2G162660	Csa3G745020	Csa7G447800	Csa6G008060
Csa2G162660	Csa6G301070	Csa7G447800	Csa6G008690
Csa2G162660	Csa6G301600	Csa7G447800	Csa6G011720
Csa2G162660	Csa6G303210	Csa7G447800	Csa6G013350
Csa2G162660	Csa7G179620	Csa7G447800	Csa6G406540
Csa2G162660	Csa2G317420	Csa7G447800	Csa6G188110
Csa2G162660	Csa2G315900	Csa7G447800	Csa6G181580
Csa2G162660	Csa2G310390	Csa7G447800	Csa3G623980
Csa2G162660	Csa7G009770	Csa7G447800	Csa3G610280
Csa2G162660	Csa7G009210	Csa7G447800	Csa1G597810
Csa2G162660	Csa7G009170	Csa7G447800	Csa1G598870
Csa2G162660	Csa7G009130	Csa7G447800	Csa1G599560
Csa2G162660	Csa7G007880	Csa7G447800	Csa1G600140
Csa2G162660	Csa2G165670	Csa7G447800	Csa1G600180
Csa2G162660	Csa2G169770	Csa7G447800	Csa1G600190
Csa2G162660	Csa2G171850	Csa7G447800	Csa1G600210
Csa2G162660	Csa2G173020	Csa7G447800	Csa1G601010
Csa2G162660	Csa2G173070	Csa7G447800	Csa1G605110
Csa2G162660	Csa2G174130	Csa7G447800	Csa1G613460
Csa2G162660	Csa2G178720	Csa7G447800	Csa1G613620
Csa2G162660	Csa2G190770	Csa7G447800	Csa1G614650

Csa2G162660	Csa2G193360	Csa7G447800	Csa5G224130
Csa2G162660	Csa6G161200	Csa7G447800	Csa5G223110
Csa2G162660	Csa6G155030	Csa7G447800	Csa5G221940
Csa2G162660	Csa6G153460	Csa7G447800	Csa5G220910
Csa2G162660	Csa6G152940	Csa7G447800	Csa5G215130
Csa2G162660	Csa6G151640	Csa7G447800	CsaUNG017140
Csa2G162660	Csa6G150490	Csa7G447800	Csa1G528510
Csa2G162660	Csa6G149410	Csa7G447800	Csa1G530140
Csa2G162660	Csa6G147670	Csa7G447800	Csa7G048050
Csa2G162660	Csa6G147620	Csa7G447800	Csa7G048000
Csa2G162660	Csa6G140840	Csa7G447800	Csa7G047370
Csa2G162660	Csa2G211450	Csa7G447800	Csa7G047290
Csa2G162660	Csa2G215490	Csa7G447800	Csa7G045500
Csa2G162660	Csa2G222070	Csa7G447800	Csa7G044950
Csa2G162660	Csa7G368080	Csa7G447800	Csa7G044910
Csa2G162660	Csa7G368090	Csa7G447800	Csa7G044870
Csa2G162660	Csa7G368100	Csa7G447800	Csa7G043610
Csa2G162660	Csa7G372880	Csa7G447800	Csa7G043020
Csa2G162660	Csa7G372960	Csa7G447800	Csa3G638540
Csa2G162660	Csa7G376370	Csa7G447800	Csa3G636390
Csa2G162660	Csa7G378470	Csa7G447800	Csa3G634360
Csa2G162660	Csa7G378500	Csa7G447800	Csa3G629740
Csa2G162660	Csa7G378510	Csa7G447800	Csa3G627690
Csa2G162660	Csa7G378520	Csa7G447800	Csa1G533530
Csa2G162660	Csa7G378540	Csa7G447800	Csa1G533630
Csa2G162660	Csa7G378550	Csa7G447800	Csa1G533660
Csa2G162660	Csa7G161030	Csa7G447800	Csa4G268070
Csa2G162660	Csa1G049460	Csa7G447800	Csa4G280520
Csa2G162660	Csa1G050000	Csa7G447800	Csa4G286310
Csa2G162660	Csa1G050210	Csa7G447800	Csa2G307850
Csa2G162660	Csa1G050220	Csa7G447800	Csa1G588520
Csa2G162660	Csa1G050250	Csa7G447800	Csa1G589090
Csa2G162660	Csa1G050290	Csa7G447800	Csa1G589650
Csa2G162660	Csa1G050310	Csa7G447800	Csa3G599460
Csa2G162660	Csa1G050450	Csa7G447800	Csa3G595200
Csa2G162660	Csa1G050470	Csa7G447800	Csa3G589540
Csa2G162660	Csa1G050520	Csa7G447800	Csa1G000600
Csa2G162660	Csa1G050580	Csa7G447800	Csa1G000610
Csa2G162660	Csa1G051600	Csa7G447800	Csa1G000700
Csa2G162660	Csa5G021310	Csa7G447800	Csa1G000730
Csa2G162660	Csa2G348290	Csa7G447800	Csa1G002040
Csa2G162660	Csa2G348810	Csa7G447800	Csa1G002080
Csa2G162660	Csa2G348820	Csa7G447800	Csa1G002160
Csa2G162660	Csa2G349050	Csa7G447800	Csa5G640510
Csa2G162660	Csa2G349090	Csa7G447800	Csa5G638360
Csa2G162660	Csa2G349620	Csa7G447800	Csa5G637800
Csa2G162660	Csa2G350340	Csa7G447800	Csa5G637790
Csa2G162660	Csa2G351020	Csa7G447800	Csa5G637160

Csa2G162660	Csa2G352400	Csa7G447800	Csa5G636570
Csa2G162660	Csa2G354030	Csa7G447800	Csa5G636510
Csa2G162660	Csa2G354680	Csa7G447800	Csa5G633190
Csa2G162660	Csa2G354730	Csa7G447800	Csa5G631520
Csa2G162660	Csa2G354750	Csa7G447800	Csa5G631510
Csa2G162660	Csa2G354950	Csa7G447800	Csa5G630930
Csa2G162660	Csa1G586880	Csa7G447800	Csa2G364570
Csa2G162660	Csa1G587400	Csa7G447800	Csa2G369080
Csa2G162660	Csa4G098710	Csa7G447800	Csa2G369740
Csa2G162660	Csa4G107370	Csa7G447800	Csa2G369840
Csa2G162660	Csa4G107430	Csa7G447800	Csa2G370350
Csa2G162660	Csa4G109020	Csa7G447800	Csa2G372160
Csa2G370430	Csa4G193240	Csa7G447800	Csa2G372740
Csa2G370430	Csa6G401320	Csa7G447800	Csa2G372750
Csa2G370430	Csa5G423880	Csa7G447800	Csa2G372820
Csa2G370430	Csa7G209560	Csa7G447800	Csa2G373590
Csa2G370430	Csa6G212890	Csa7G447800	Csa2G375760
Csa2G370430	Csa5G517190	Csa7G447800	Csa2G376790
Csa2G370430	Csa5G524700	Csa7G447800	Csa2G379120
Csa2G370430	Csa5G550220	Csa7G447800	Csa2G379300
Csa2G370430	Csa5G547590	Csa7G447800	Csa2G379350
Csa2G370430	Csa3G360140	Csa7G447800	Csa2G382710
Csa2G370430	Csa3G354520	Csa7G447800	Csa2G384430
Csa2G370430	Csa3G354500	Csa7G447800	Csa1G572440
Csa2G370430	Csa4G358660	Csa7G447800	Csa5G190470
Csa2G370430	Csa4G141210	Csa7G447800	Csa5G180840
Csa2G370430	Csa7G395260	Csa7G447800	Csa5G179740
Csa2G370430	Csa4G028980	Csa7G447800	Csa5G179230
Csa2G370430	Csa5G628660	Csa7G447800	Csa5G175910
Csa2G370430	Csa3G280960	Csa7G447800	Csa5G175780
Csa2G370430	Csa3G252480	Csa7G447800	Csa5G173520
Csa2G370430	Csa3G047780	Csa7G447800	Csa1G537530
Csa2G370430	Csa3G063110	Csa7G447800	Csa1G538820
Csa2G370430	Csa3G066750	Csa7G447800	Csa1G539350
Csa2G370430	Csa3G073930	Csa7G447800	Csa2G223720
Csa2G370430	Csa6G504410	Csa7G447800	Csa2G224260
Csa2G370430	Csa6G506030	Csa7G447800	Csa2G264610
Csa2G370430	Csa6G507260	Csa7G447800	Csa4G454660
Csa2G370430	Csa6G510860	Csa7G447800	Csa1G257870
Csa2G370430	Csa6G511740	Csa7G447800	Csa1G276450
Csa2G370430	Csa6G511760	Csa7G447800	Csa6G191560
Csa2G370430	Csa6G511770	Csa7G447800	Csa6G191550
Csa2G370430	Csa6G511820	Csa7G447800	Csa6G190450
Csa2G370430	Csa6G513550	Csa7G447800	Csa5G317890
Csa2G370430	Csa6G516640	Csa7G447800	Csa5G023910
Csa2G370430	Csa6G516730	Csa7G447800	Csa4G129620
Csa2G370430	Csa6G519660	Csa7G447800	Csa4G129560
Csa2G370430	Csa6G520430	Csa7G447800	Csa4G128010

Csa2G370430	Csa6G520990	Csa7G447800	Csa3G734960
Csa2G370430	Csa6G522670	Csa7G447800	Csa3G734270
Csa2G370430	Csa6G522710	Csa7G447800	Csa3G734150
Csa2G370430	Csa6G523980	Csa7G447800	Csa3G733980
Csa2G370430	Csa6G526330	Csa7G447800	Csa3G732620
Csa2G370430	Csa6G538760	Csa7G447800	Csa3G730820
Csa2G370430	Csa5G262270	Csa7G447800	Csa3G728100
Csa2G370430	Csa1G561360	Csa7G447800	Csa3G710850
Csa2G370430	Csa1G561400	Csa7G447800	Csa3G708170
Csa2G370430	Csa3G402460	Csa7G447800	Csa3G707170
Csa2G370430	Csa6G362970	Csa7G447800	Csa2G079650
Csa2G370430	Csa6G365180	Csa7G447800	Csa2G075330
Csa2G370430	Csa4G595990	Csa7G447800	Csa2G070310
Csa2G370430	Csa3G080340	Csa7G447800	Csa4G064070
Csa2G370430	Csa3G078250	Csa7G447800	Csa4G063460
Csa2G370430	Csa3G483780	Csa7G447800	Csa4G056710
Csa2G370430	Csa3G483760	Csa7G447800	Csa1G182070
Csa2G370430	Csa3G483750	Csa7G447800	Csa1G181480
Csa2G370430	Csa4G291890	Csa7G447800	Csa1G181420
Csa2G370430	Csa4G293160	Csa7G447800	Csa1G181350
Csa2G370430	Csa4G293300	Csa7G447800	Csa1G181340
Csa2G370430	Csa4G295440	Csa7G447800	Csa1G180760
Csa2G370430	Csa4G296160	Csa7G447800	Csa1G179740
Csa2G370430	Csa4G296230	Csa7G447800	Csa1G569300
Csa2G370430	Csa4G303130	Csa7G447800	Csa1G570090
Csa2G370430	Csa4G308550	Csa7G447800	Csa1G570110
Csa2G370430	Csa4G308580	Csa7G447800	Csa5G070180
Csa2G370430	Csa5G312840	Csa7G447800	Csa6G410060
Csa2G370430	Csa4G430820	Csa7G447800	Csa6G411220
Csa2G370430	Csa2G417830	Csa7G447800	Csa6G422830
Csa2G370430	Csa3G099670	Csa7G447800	Csa6G423450
Csa2G370430	Csa3G106000	Csa7G447800	Csa6G425040
Csa2G370430	Csa5G593380	Csa7G447800	Csa6G425750
Csa2G370430	Csa5G593330	Csa7G447800	Csa6G425840
Csa2G370430	Csa5G591760	Csa7G447800	Csa6G426370
Csa2G370430	Csa5G590720	Csa7G447800	Csa5G284510
Csa2G370430	Csa5G590030	Csa7G447800	Csa6G385070
Csa2G370430	Csa5G589960	Csa7G447800	Csa4G669240
Csa2G370430	Csa5G583280	Csa7G447800	Csa3G651720
Csa2G370430	Csa6G338120	Csa7G447800	Csa5G140460
Csa2G370430	Csa6G357030	Csa7G447800	Csa5G140530
Csa2G370430	Csa7G343310	Csa7G447800	Csa5G146890
Csa2G370430	Csa7G338120	Csa7G447800	Csa2G138720
Csa2G370430	Csa7G336530	Csa7G447800	Csa2G139850
Csa2G370430	Csa7G325200	Csa7G447800	Csa3G696880
Csa2G370430	Csa7G290470	Csa7G447800	Csa1G002830
Csa2G370430	Csa6G450370	Csa7G447800	Csa1G004230
Csa2G370430	Csa6G452680	Csa7G447800	Csa1G005710

Csa2G370430	Csa6G452690	Csa7G447800	Csa1G005740
Csa2G370430	Csa7G388450	Csa7G447800	Csa1G008410
Csa2G370430	Csa1G062920	Csa7G447800	Csa1G009670
Csa2G370430	Csa1G063480	Csa7G447800	Csa1G009680
Csa2G370430	Csa1G063590	Csa7G447800	Csa1G011590
Csa2G370430	Csa1G064710	Csa7G447800	Csa1G015040
Csa2G370430	Csa6G428000	Csa7G447800	Csa1G015610
Csa2G370430	Csa6G430720	Csa7G447800	Csa1G015690
Csa2G370430	Csa2G408960	Csa7G447800	Csa1G015760
Csa2G370430	Csa2G405050	Csa7G447800	Csa1G015860
Csa2G370430	Csa2G404990	Csa7G447800	Csa1G015880
Csa2G370430	Csa2G403710	Csa7G447800	Csa1G022500
Csa2G370430	Csa2G401440	Csa7G447800	Csa1G023070
Csa2G370430	Csa2G401370	Csa7G447800	Csa1G023080
Csa2G370430	Csa2G036660	Csa7G447800	Csa1G153520
Csa2G370430	Csa2G035520	Csa7G447800	Csa1G145960
Csa2G370430	Csa2G032760	Csa7G447800	Csa1G134270
Csa2G370430	Csa2G031190	Csa7G447800	Csa4G043950
Csa2G370430	Csa2G030610	Csa7G447800	Csa4G043840
Csa2G370430	Csa2G021690	Csa7G447800	Csa4G043830
Csa2G370430	Csa2G021680	Csa7G447800	Csa4G038760
Csa2G370430	Csa1G422430	Csa7G447800	Csa4G038740
Csa2G370430	Csa1G423110	Csa7G447800	Csa4G036590
Csa2G370430	Csa1G256200	Csa7G447800	Csa4G036580
Csa2G370430	Csa1G256210	Csa7G447800	Csa5G139860
Csa2G370430	Csa1G256720	Csa7G447800	Csa5G139590
Csa2G370430	Csa4G026910	Csa7G447800	Csa5G139580
Csa2G370430	Csa4G026900	Csa7G447800	Csa5G139170
Csa2G370430	Csa4G026280	Csa7G447800	Csa5G139100
Csa2G370430	Csa4G025050	Csa7G447800	Csa5G138490
Csa2G370430	Csa4G017110	Csa7G447800	Csa7G412870
Csa2G370430	Csa4G008240	Csa7G447800	Csa7G407770
Csa2G370430	Csa4G007600	Csa7G447800	Csa7G407730
Csa2G370430	Csa4G003090	Csa7G447800	Csa7G407680
Csa2G370430	Csa4G002500	Csa7G447800	Csa7G407660
Csa2G370430	Csa4G001800	Csa7G447800	Csa7G407610
Csa2G370430	Csa4G00990	Csa7G447800	Csa7G406990
Csa2G370430	Csa4G000870	Csa7G447800	Csa7G396470
Csa2G370430	Csa1G044870	Csa7G447800	Csa7G396430
Csa2G370430	Csa1G044880	Csa7G447800	Csa7G396420
Csa2G370430	Csa1G045750	Csa7G447800	Csa1G096100
Csa2G370430	Csa1G045760	Csa7G447800	Csa1G458990
Csa2G370430	Csa1G046100	Csa7G447800	Csa1G467060
Csa2G370430	Csa1G046180	Csa7G447800	Csa1G467100
Csa2G370430	Csa7G027870	Csa7G447800	Csa1G469730
Csa2G370430	Csa7G029410	Csa7G447800	Csa1G478080
Csa2G370430	Csa6G483410	Csa7G447800	Csa1G479630
Csa2G370430	Csa6G484600	Csa7G447800	Csa1G505960

Csa2G370430	Csa6G486670	Csa7G447800	Csa7G065130
Csa2G370430	Csa6G486940	Csa7G447800	Csa7G067530
Csa2G370430	Csa6G487810	Csa7G447800	Csa7G069720
Csa2G370430	Csa6G488360	Csa7G447800	Csa7G070810
Csa2G370430	Csa6G489380	Csa7G447800	Csa7G071440
Csa2G370430	Csa6G489980	Csa7G447800	Csa7G071470
Csa2G370430	Csa6G492280	Csa7G447800	Csa7G071560
Csa2G370430	Csa6G495020	Csa7G447800	Csa7G071670
Csa2G370430	Csa6G497020	Csa7G447800	Csa7G072870
Csa2G370430	Csa6G497150	Csa7G447800	Csa7G073450
Csa2G370430	Csa6G498930	Csa7G447800	Csa7G074880
Csa2G370430	Csa6G498960	Csa7G447800	Csa1G084830
Csa2G370430	Csa6G498970	Csa7G447800	Csa2G340400
Csa2G370430	Csa6G499190	Csa7G447800	Csa2G336670
Csa2G370430	Csa6G499780	Csa7G447800	Csa2G336140
Csa2G370430	Csa6G500420	Csa7G447800	Csa2G336080
Csa2G370430	Csa6G500590	Csa7G447800	Csa2G334540
Csa2G370430	Csa6G500670	Csa7G447800	Csa2G361790
Csa2G370430	Csa4G045070	Csa7G447800	Csa2G361640
Csa2G370430	Csa4G046670	Csa7G447800	Csa2G360740
Csa2G370430	Csa4G046720	Csa7G447800	Csa2G360700
Csa2G370430	Csa4G046790	Csa7G447800	Csa2G358880
Csa2G370430	Csa4G048010	Csa7G447800	Csa2G271380
Csa2G370430	Csa4G051360	Csa7G447800	Csa2G270810
Csa2G370430	Csa4G051470	Csa7G447800	Csa2G270750
Csa2G370430	Csa3G181990	Csa7G447800	Csa2G270150
Csa2G370430	Csa3G182100	Csa7G447800	Csa2G270140
Csa2G370430	Csa3G184030	Csa7G447800	Csa7G201830
Csa2G370430	Csa2G245500	Csa7G447800	Csa1G704590
Csa2G370430	Csa2G237130	Csa7G447800	Csa1G715230
Csa2G370430	Csa1G024130	Csa7G447800	Csa5G650570
Csa2G370430	Csa1G024170	Csa7G447800	Csa5G650520
Csa2G370430	Csa1G024210	Csa7G447800	Csa5G649910
Csa2G370430	Csa1G025020	Csa7G447800	Csa5G648740
Csa2G370430	Csa1G025950	Csa7G447800	Csa5G647490
Csa2G370430	Csa1G031790	Csa7G447800	Csa5G647480
Csa2G370430	Csa1G031860	Csa7G447800	Csa5G647340
Csa2G370430	Csa1G033010	Csa7G447800	Csa5G646670
Csa2G370430	Csa1G033250	Csa7G447800	Csa5G643880
Csa2G370430	Csa1G039030	Csa7G447800	Csa5G643380
Csa2G370430	Csa1G041550	Csa7G447800	Csa7G234670
Csa2G370430	Csa1G042170	Csa7G447800	Csa7G237830
Csa2G370430	Csa1G042200	Csa7G447800	Csa7G238440
Csa2G370430	Csa2G006000	Csa7G447800	Csa7G257340
Csa2G370430	Csa2G005340	Csa7G447800	Csa7G278200
Csa2G370430	Csa2G003600	Csa7G447800	Csa7G279240
Csa2G370430	Csa2G000630	Csa7G447800	Csa7G281360
Csa2G370430	Csa2G000470	Csa7G447800	Csa7G281380

Csa2G370430	Csa1G515470	Csa7G447800	Csa3G776860
Csa2G370430	Csa6G135470	Csa7G447800	Csa3G776900
Csa2G370430	Csa6G133710	Csa7G447800	Csa3G777610
Csa2G370430	Csa6G128570	Csa7G447800	Csa3G778360
Csa2G370430	Csa6G128030	Csa7G447800	Csa3G778370
Csa2G370430	Csa6G124190	Csa7G447800	Csa3G778990
Csa2G370430	Csa6G124130	Csa7G447800	Csa3G782760
Csa2G370430	Csa6G123990	Csa7G447800	Csa3G783840
Csa2G370430	Csa6G123980	Csa7G447800	Csa3G785400
Csa2G370430	Csa6G113510	Csa7G447800	Csa3G790960
Csa2G370430	Csa6G109120	Csa7G447800	Csa3G800710
Csa2G370430	Csa1G042280	Csa7G447800	Csa3G808380
Csa2G370430	Csa1G042440	Csa7G447800	Csa3G809400
Csa2G370430	Csa1G042600	Csa7G447800	Csa3G207390
Csa2G370430	Csa1G042790	Csa7G447800	Csa3G207350
Csa2G370430	Csa1G042920	Csa7G447800	Csa3G200700
Csa2G370430	Csa4G628910	Csa7G447800	Csa5G365160
Csa2G370430	Csa4G631570	Csa7G447800	Csa5G375760
Csa2G370430	Csa4G637760	Csa7G447800	Csa3G684170
Csa2G370430	Csa4G638370	Csa7G447800	Csa3G686210
Csa2G370430	Csa4G639210	Csa7G447800	Csa3G687750
Csa2G370430	Csa6G448720	Csa7G447800	Csa3G690840
Csa2G370430	Csa1G071940	Csa7G447800	Csa1G226410
Csa2G370430	Csa1G073650	Csa7G447800	Csa2G302120
Csa2G370430	Csa1G075060	Csa7G447800	Csa2G299870
Csa2G370430	Csa6G502810	Csa7G447800	Csa2G299880
Csa2G370430	Csa7G428210	Csa7G447800	Csa2G297190
Csa2G370430	Csa7G420790	Csa7G447800	Csa2G296070
Csa2G370430	Csa7G420740	Csa7G447800	Csa2G296010
Csa2G370430	Csa7G420700	Csa7G447800	Csa3G180440
Csa2G370430	Csa7G419590	Csa7G447800	Csa3G178560
Csa2G370430	Csa7G414510	Csa7G447800	Csa3G178490
Csa2G370430	Csa5G166430	Csa7G447800	Csa3G177950
Csa2G370430	Csa2G008070	Csa7G447800	Csa3G176320
Csa2G370430	Csa2G008080	Csa7G447800	Csa3G172920
Csa2G370430	Csa2G008110	Csa7G447800	Csa3G171230
Csa2G370430	Csa2G010270	Csa7G447800	Csa3G168390
Csa2G370430	Csa1G056980	Csa7G447800	Csa3G166340
Csa2G370430	Csa1G056940	Csa7G447800	Csa3G166300
Csa2G370430	Csa6G005130	Csa7G447800	Csa3G166280
Csa2G370430	Csa6G008760	Csa7G447800	Csa3G164500
Csa2G370430	Csa6G013320	Csa7G447800	Csa3G154370
Csa2G370430	Csa6G013330	Csa7G447800	Csa3G150120
Csa2G370430	Csa3G302100	Csa7G447800	Csa3G150110
Csa2G370430	Csa6G405960	Csa7G447800	Csa3G149380
Csa2G370430	Csa6G188110	Csa7G447800	Csa3G144740
Csa2G370430	Csa6G188070	Csa7G447800	Csa3G144220
Csa2G370430	Csa6G188050	Csa7G447800	Csa3G144180

Csa2G370430	Csa6G180980	Csa7G447800	Csa3G144140
Csa2G370430	Csa3G611380	Csa7G447800	Csa3G135110
Csa2G370430	Csa1G599450	Csa7G447800	Csa3G134010
Csa2G370430	Csa1G604600	Csa7G447800	Csa3G133970
Csa2G370430	Csa1G605720	Csa7G447800	Csa3G133360
Csa2G370430	Csa1G612900	Csa7G447800	Csa3G133260
Csa2G370430	Csa1G524760	Csa7G447800	Csa3G133110
Csa2G370430	Csa1G528580	Csa7G447800	Csa3G129750
Csa2G370430	Csa1G529120	Csa7G447800	Csa3G129630
Csa2G370430	Csa7G047340	Csa7G447800	Csa3G129490
Csa2G370430	Csa7G044260	Csa7G447800	Csa3G127170
Csa2G370430	Csa3G644850	Csa7G447800	Csa3G126860
Csa2G370430	Csa3G644790	Csa7G447800	Csa3G126810
Csa2G370430	Csa3G637990	Csa7G447800	Csa3G126140
Csa2G370430	Csa7G219810	Csa7G447800	Csa3G124760
Csa2G370430	Csa4G269120	Csa7G447800	Csa3G124240
Csa2G370430	Csa4G286930	Csa7G447800	Csa3G123190
Csa2G370430	Csa1G002690	Csa7G447800	Csa3G121040
Csa2G370430	Csa5G638470	Csa7G447800	Csa3G119830
Csa2G370430	Csa5G638410	Csa7G447800	Csa3G119820
Csa2G370430	Csa5G633280	Csa7G447800	Csa3G119670
Csa2G370430	Csa5G633250	Csa7G447800	Csa3G119580
Csa2G370430	Csa5G630820	Csa7G447800	Csa3G119560
Csa2G370430	Csa2G367240	Csa7G447800	Csa3G118730
Csa2G370430	Csa2G368950	Csa7G447800	Csa3G115100
Csa2G370430	Csa2G369070	Csa7G447800	Csa6G238650
Csa2G370430	Csa2G369760	Csa7G447800	Csa5G056620
Csa2G370430	Csa2G370420	Csa7G447800	Csa7G379080
Csa2G370430	Csa2G370470	Csa7G447800	Csa2G108690
Csa2G370430	Csa2G380630	Csa7G447800	Csa2G118280
Csa2G370430	Csa2G382700	Csa7G447800	Csa2G122000
Csa2G370430	Csa2G385000	Csa7G447800	Csa2G130670
Csa2G370430	Csa5G182720	Csa7G447800	Csa2G138190
Csa2G370430	Csa5G179230	Csa7G447800	Csa3G416650
Csa2G370430	Csa5G177690	Csa7G447800	Csa3G415090
Csa2G370430	Csa5G177110	Csa7G447800	Csa4G649660
Csa2G370430	Csa5G176010	Csa7G447800	Csa4G651780
Csa2G370430	Csa5G175740	Csa7G447800	Csa4G651840
Csa2G370430	Csa5G174580	Csa7G447800	Csa4G652070
Csa2G370430	Csa1G538140	Csa7G447800	Csa4G652740
Csa2G370430	Csa1G541390	Csa7G447800	Csa4G653410
Csa2G370430	Csa2G263980	Csa7G447800	Csa4G653480
Csa2G370430	Csa4G443130	Csa7G447800	Csa4G090320
Csa2G370430	Csa1G257900	Csa7G447800	Csa4G088720
Csa2G370430	Csa1G257950	Csa7G447800	Csa4G083690
Csa2G370430	Csa6G199780	Csa7G447800	Csa4G083500
Csa2G370430	Csa6G198280	Csa7G447800	Csa5G494420
Csa2G370430	Csa6G193620	Csa7G447800	Csa5G495960

Csa2G370430	Csa6G191580	Csa7G447800	Csa5G496480
Csa2G370430	Csa6G395130	Csa7G447800	Csa5G497020
Csa2G370430	Csa5G023900	Csa7G447800	Csa5G276460
Csa2G370430	Csa4G129040	Csa7G447800	Csa5G272920
Csa2G370430	Csa3G734930	Csa7G447800	Csa5G269890
Csa2G370430	Csa3G734230	Csa7G447800	Csa2G083760
Csa2G370430	Csa3G732620	Csa7G447800	Csa2G084270
Csa2G370430	Csa3G731870	Csa7G447800	Csa4G242880
Csa2G370430	Csa3G731720	Csa7G447800	Csa7G101780
Csa2G370430	Csa3G731210	Csa7G447800	Csa5G568300
Csa2G370430	Csa3G731150	Csa7G447800	Csa5G569320
Csa2G370430	Csa3G730850	Csa7G447800	Csa5G570390
Csa2G370430	Csa3G730840	Csa7G447800	Csa5G571440
Csa2G370430	Csa3G730810	Csa7G447800	Csa5G571500
Csa2G370430	Csa3G730760	Csa7G447800	Csa5G576650
Csa2G370430	Csa2G075400	Csa7G447800	Csa5G576660
Csa2G370430	Csa4G064020	Csa7G447800	Csa5G577450
Csa2G370430	Csa1G181330	Csa7G447800	Csa5G578990
Csa2G370430	Csa7G037620	Csa7G447800	Csa1G433060
Csa2G370430	Csa7G038680	Csa7G447800	Csa1G427530
Csa2G370430	Csa1G569320	Csa7G447800	Csa5G613450
Csa2G370430	Csa1G569400	Csa7G447800	Csa5G613440
Csa2G370430	Csa6G409330	Csa7G447800	Csa5G612900
Csa2G370430	Csa6G409910	Csa7G447800	Csa5G610520
Csa2G370430	Csa6G421570	Csa7G447800	Csa5G610420
Csa2G370430	Csa6G421650	Csa7G447800	Csa5G610360
Csa2G370430	Csa6G426880	Csa7G447800	Csa5G609810
Csa2G370430	Csa4G669240	Csa7G447800	Csa5G609740
Csa2G370430	Csa3G653460	Csa7G447800	Csa5G608570
Csa2G370430	Csa5G146210	Csa7G447800	Csa5G608320
Csa2G370430	Csa5G146930	Csa7G447800	Csa5G608250
Csa2G370430	Csa5G146970	Csa7G447800	Csa5G608190
Csa2G370430	Csa2G138770	Csa7G447800	Csa5G608140
Csa2G370430	Csa2G140370	Csa7G447800	Csa5G607450
Csa2G370430	Csa2G147920	Csa7G447800	Csa5G606780
Csa2G370430	Csa7G049240	Csa7G447800	Csa5G606580
Csa2G370430	Csa1G007870	Csa7G447800	Csa5G606270
Csa2G370430	Csa1G008440	Csa7G447800	Csa5G605730
Csa2G370430	Csa1G009590	Csa7G447800	Csa5G605080
Csa2G370430	Csa1G009770	Csa7G447800	Csa5G604410
Csa2G370430	Csa1G010940	Csa7G447800	Csa5G603980
Csa2G370430	Csa1G011590	Csa7G447800	Csa5G602750
Csa2G370430	Csa1G012130	Csa7G447800	Csa5G602190
Csa2G370430	Csa1G013770	Csa7G447800	Csa5G601610
Csa2G370430	Csa1G014490	Csa7G447800	Csa5G600940
Csa2G370430	Csa1G015710	Csa7G447800	Csa5G598740
Csa2G370430	Csa1G015820	Csa7G447800	Csa5G598060
Csa2G370430	Csa1G022520	Csa7G447800	Csa5G593440

Csa2G370430	Csa1G023040	Csa7G447800	Csa5G593400
Csa2G370430	Csa1G023070	Csa7G447800	Csa1G575100
Csa2G370430	Csa1G152500	Csa7G447800	Csa1G575090
Csa2G370430	Csa1G145970	Csa7G447800	Csa1G575040
Csa2G370430	Csa1G144280	Csa7G447800	Csa1G574980
Csa2G370430	Csa1G136780	Csa7G447800	Csa1G573670
Csa2G370430	Csa4G043920	Csa7G447800	Csa4G075190
Csa2G370430	Csa5G139260	Csa7G447800	Csa1G689640
Csa2G370430	Csa5G139090	Csa7G447800	Csa1G690280
Csa2G370430	Csa7G407570	Csa7G447800	Csa1G103270
Csa2G370430	Csa7G407550	Csa7G447800	Csa1G320310
Csa2G370430	Csa7G404800	Csa7G447800	CsaUNG009910
Csa2G370430	Csa7G396350	Csa7G447800	Csa4G000550
Csa2G370430	Csa1G086390	Csa7G447800	Csa4G646130
Csa2G370430	Csa1G086920	Csa7G447800	CsaUNG005730
Csa2G370430	Csa1G103260	Csa7G447800	Csa3G539670
Csa2G370430	Csa2G062650	Csa7G447800	Csa3G509940
Csa2G370430	Csa1G448920	Csa7G447800	Csa5G162040
Csa2G370430	Csa1G467120	Csa7G447800	Csa5G157240
Csa2G370430	Csa1G479610	Csa7G447800	Csa5G156190
Csa2G370430	Csa1G479630	Csa7G447800	Csa5G155580
Csa2G370430	Csa1G481210	Csa7G447800	Csa5G153170
Csa2G370430	Csa1G495280	Csa7G447800	Csa5G152920
Csa2G370430	Csa7G064050	Csa7G447800	Csa5G152860
Csa2G370430	Csa7G065140	Csa7G447800	Csa5G150970
Csa2G370430	Csa7G066310	Csa7G447800	Csa5G149870
Csa2G370430	Csa7G066830	Csa7G447800	Csa5G148610
Csa2G370430	Csa7G071590	Csa7G447800	Csa5G148550
Csa2G370430	Csa7G073530	Csa7G447800	Csa3G743420
Csa2G370430	Csa7G073750	Csa7G447800	Csa3G740820
Csa2G370430	Csa1G084320	Csa7G447800	Csa3G740220
Csa2G370430	Csa2G338890	Csa7G447800	Csa3G739040
Csa2G370430	Csa2G336150	Csa7G447800	Csa3G738990
Csa2G370430	Csa2G362450	Csa7G447800	Csa3G738980
Csa2G370430	Csa2G361800	Csa7G447800	Csa3G736960
Csa2G370430	Csa2G361450	Csa7G447800	Csa3G736900
Csa2G370430	Csa2G360850	Csa7G447800	Csa3G736820
Csa2G370430	Csa2G360590	Csa7G447800	Csa3G734980
Csa2G370430	Csa2G360030	Csa7G447800	Csa5G626030
Csa2G370430	Csa2G271400	Csa7G447800	Csa5G623840
Csa2G370430	Csa1G366980	Csa7G447800	Csa5G623580
Csa2G370430	Csa1G397110	Csa7G447800	Csa5G622840
Csa2G370430	Csa5G645150	Csa7G447800	Csa5G622660
Csa2G370430	Csa7G236280	Csa7G447800	Csa5G622550
Csa2G370430	Csa7G239050	Csa7G447800	Csa5G622530
Csa2G370430	Csa7G253210	Csa7G447800	Csa5G622500
Csa2G370430	Csa7G259350	Csa7G447800	Csa3G880550
Csa2G370430	Csa7G272130	Csa7G447800	Csa3G881590

Csa2G370430	Csa7G284440	Csa7G447800	Csa3G881630
Csa2G370430	Csa3G777560	Csa7G447800	Csa3G881650
Csa2G370430	Csa3G778340	Csa7G447800	Csa3G881690
Csa2G370430	Csa3G778440	Csa7G447800	Csa3G881790
Csa2G370430	Csa3G780530	Csa7G447800	Csa3G881820
Csa2G370430	Csa3G782690	Csa7G447800	Csa3G881910
Csa2G370430	Csa3G808360	Csa7G447800	Csa3G881920
Csa2G370430	Csa3G207940	Csa7G447800	Csa3G882970
Csa2G370430	Csa3G202720	Csa7G447800	Csa3G882990
Csa2G370430	Csa3G199600	Csa7G447800	Csa3G889750
Csa2G370430	Csa5G373210	Csa7G447800	Csa3G889780
Csa2G370430	Csa2G297760	Csa7G447800	Csa3G889830
Csa2G370430	Csa2G295940	Csa7G447800	Csa3G889910
Csa2G370430	Csa3G172970	Csa7G447800	Csa3G889920
Csa2G370430	Csa3G151510	Csa7G447800	Csa3G891660
Csa2G370430	Csa3G151410	Csa7G447800	Csa3G893440
Csa2G370430	Csa3G146550	Csa7G447800	Csa3G894460
Csa2G370430	Csa3G143580	Csa7G447800	Csa3G895630
Csa2G370430	Csa3G141900	Csa7G447800	Csa3G895690
Csa2G370430	Csa3G141820	Csa7G447800	Csa3G895750
Csa2G370430	Csa3G135070	Csa7G447800	Csa3G895870
Csa2G370430	Csa3G135010	Csa7G447800	Csa3G895900
Csa2G370430	Csa3G134790	Csa7G447800	Csa3G901040
Csa2G370430	Csa3G134730	Csa7G447800	Csa3G901190
Csa2G370430	Csa3G133320	Csa7G447800	Csa3G902320
Csa2G370430	Csa3G132550	Csa7G447800	Csa3G902390
Csa2G370430	Csa3G132010	Csa7G447800	Csa3G902400
Csa2G370430	Csa3G131970	Csa7G447800	Csa3G903520
Csa2G370430	Csa3G130320	Csa7G447800	Csa3G910720
Csa2G370430	Csa3G129660	Csa7G447800	Csa3G912370
Csa2G370430	Csa3G126240	Csa7G447800	Csa3G914030
Csa2G370430	Csa3G126200	Csa7G447800	Csa3G914050
Csa2G370430	Csa3G124980	Csa7G447800	Csa6G308420
Csa2G370430	Csa3G124840	Csa7G447800	Csa2G324430
Csa2G370430	Csa3G122490	Csa7G447800	Csa2G324450
Csa2G370430	Csa3G121750	Csa7G447800	Csa2G437050
Csa2G370430	Csa3G121740	Csa7G447800	Csa2G435510
Csa2G370430	Csa3G121730	Csa7G447800	Csa1G173210
Csa2G370430	Csa3G121050	Csa7G447800	Csa1G169420
Csa2G370430	Csa3G120480	Csa7G447800	Csa1G165730
Csa2G370430	Csa3G119850	Csa7G447800	Csa3G038170
Csa2G370430	Csa6G238650	Csa7G447800	Csa3G036560
Csa2G370430	Csa3G408540	Csa7G447800	Csa3G019360
Csa2G370430	Csa4G648530	Csa7G447800	Csa3G017010
Csa2G370430	Csa4G648560	Csa7G447800	Csa3G011850
Csa2G370430	Csa4G651890	Csa7G447800	Csa3G009460
Csa2G370430	Csa4G651970	Csa7G447800	Csa3G009450
Csa2G370430	Csa4G652140	Csa7G447800	Csa3G008910

Csa2G370430	Csa4G652810	Csa7G447800	Csa3G008330
Csa2G370430	Csa4G652880	Csa7G447800	Csa3G005540
Csa2G370430	Csa4G658500	Csa7G447800	Csa3G005030
Csa2G370430	Csa4G658580	Csa7G447800	Csa3G002900
Csa2G370430	Csa4G663700	Csa7G447800	Csa3G002830
Csa2G370430	Csa4G095550	Csa7G447800	Csa3G002660
Csa2G370430	Csa4G088750	Csa7G447800	Csa3G002590
Csa2G370430	Csa5G273440	Csa7G447800	Csa3G002500
Csa2G370430	Csa5G266840	Csa7G447800	Csa3G002490
Csa2G370430	Csa5G568810	Csa7G447800	Csa6G309960
Csa2G370430	Csa5G571480	Csa7G447800	Csa6G309980
Csa2G370430	Csa5G576650	Csa7G447800	Csa6G014820
Csa2G370430	Csa5G579610	Csa7G447800	Csa6G014730
Csa2G370430	Csa5G582200	Csa7G447800	Csa6G013940
Csa2G370430	Csa1G425940	Csa7G447800	Csa6G381850
Csa2G370430	Csa5G613470	Csa7G447800	Csa4G182220
Csa2G370430	Csa5G612820	Csa7G447800	Csa4G179150
Csa2G370430	Csa5G611700	Csa7G447800	Csa1G699560
Csa2G370430	Csa5G611610	Csa7G447800	Csa1G699580
Csa2G370430	Csa5G608070	Csa7G447800	Csa1G700690
Csa2G370430	Csa5G606910	Csa7G447800	Csa1G701380
Csa2G370430	Csa5G606630	Csa7G447800	Csa1G701390
Csa2G370430	Csa5G606580	Csa7G447800	Csa1G615710
Csa2G370430	Csa5G606480	Csa7G447800	Csa1G616250
Csa2G370430	Csa5G606320	Csa7G447800	Csa4G187830
Csa2G370430	Csa5G605040	Csa7G447800	Csa4G188990
Csa2G370430	Csa5G605020	Csa7G447800	Csa5G441650
Csa2G370430	Csa5G605010	Csa7G447800	Csa3G812170
Csa2G370430	Csa5G604230	Csa7G447800	Csa3G812230
Csa2G370430	Csa1G574870	Csa7G447800	Csa3G812750
Csa2G370430	Csa1G573620	Csa7G447800	Csa3G816030
Csa2G370430	Csa1G690300	Csa7G447800	Csa3G816160
Csa2G370430	Csa4G000680	Csa7G447800	Csa3G816170
Csa2G370430	Csa4G000600	Csa7G447800	Csa3G817710
Csa2G370430	Csa4G000580	Csa7G447800	Csa3G817750
Csa2G370430	Csa4G646410	Csa7G447800	Csa3G819910
Csa2G370430	Csa3G543180	Csa7G447800	Csa3G820500
Csa2G370430	Csa5G162630	Csa7G447800	Csa3G821590
Csa2G370430	Csa5G162080	Csa7G447800	Csa3G822190
Csa2G370430	Csa5G158530	Csa7G447800	Csa3G822250
Csa2G370430	Csa5G157400	Csa7G447800	Csa3G822390
Csa2G370430	Csa5G157330	Csa7G447800	Csa3G823060
Csa2G370430	Csa5G155600	Csa7G447800	Csa3G824200
Csa2G370430	Csa5G154850	Csa7G447800	Csa3G824870
Csa2G370430	Csa5G154200	Csa7G447800	Csa3G825010
Csa2G370430	Csa5G152790	Csa7G447800	Csa3G826680
Csa2G370430	Csa5G149330	Csa7G447800	Csa3G827250
Csa2G370430	Csa3G740120	Csa7G447800	Csa3G827290

Csa2G370430	Csa3G736830	Csa7G447800	Csa3G827300
Csa2G370430	Csa3G736780	Csa7G447800	Csa3G827400
Csa2G370430	Csa3G735030	Csa7G447800	Csa3G829120
Csa2G370430	Csa5G626030	Csa7G447800	Csa3G829150
Csa2G370430	Csa5G623760	Csa7G447800	Csa3G829170
Csa2G370430	Csa5G622680	Csa7G447800	Csa3G836500
Csa2G370430	Csa5G622560	Csa7G447800	Csa3G838700
Csa2G370430	Csa5G622550	Csa7G447800	Csa3G838750
Csa2G370430	Csa2G415560	Csa7G447800	Csa3G840410
Csa2G370430	Csa3G878800	Csa7G447800	Csa3G840960
Csa2G370430	Csa3G878820	Csa7G447800	Csa3G841480
Csa2G370430	Csa3G878860	Csa7G447800	Csa3G842110
Csa2G370430	Csa3G878890	Csa7G447800	Csa3G844920
Csa2G370430	Csa3G878940	Csa7G447800	Csa3G846010
Csa2G370430	Csa3G879490	Csa7G447800	Csa3G847610
Csa2G370430	Csa3G881780	Csa7G447800	Csa3G847620
Csa2G370430	Csa3G881840	Csa7G447800	Csa3G848820
Csa2G370430	Csa3G881870	Csa7G447800	Csa3G848860
Csa2G370430	Csa3G883010	Csa7G447800	Csa3G850530
Csa2G370430	Csa3G888530	Csa7G447800	Csa3G852600
Csa2G370430	Csa3G889710	Csa7G447800	Csa3G852630
Csa2G370430	Csa3G889790	Csa7G447800	Csa3G855310
Csa2G370430	Csa3G889970	Csa7G447800	Csa3G855350
Csa2G370430	Csa3G894510	Csa7G447800	Csa3G855410
Csa2G370430	Csa3G894550	Csa7G447800	Csa3G856010
Csa2G370430	Csa3G901150	Csa7G447800	Csa3G857580
Csa2G370430	Csa3G902950	Csa7G447800	Csa3G872080
Csa2G370430	Csa2G324420	Csa7G447800	Csa3G872160
Csa2G370430	Csa2G324450	Csa7G447800	Csa3G874320
Csa2G370430	Csa5G457760	Csa7G447800	Csa7G058560
Csa2G370430	Csa1G169930	Csa7G447800	Csa6G087700
Csa2G370430	Csa1G166260	Csa7G447800	Csa6G087870
Csa2G370430	Csa3G038100	Csa7G447800	Csa6G087980
Csa2G370430	Csa3G036410	Csa7G447800	Csa6G088010
Csa2G370430	Csa3G019930	Csa7G447800	Csa6G089270
Csa2G370430	Csa3G019900	Csa7G447800	Csa6G091980
Csa2G370430	Csa3G019390	Csa7G447800	Csa6G094690
Csa2G370430	Csa3G018320	Csa7G447800	Csa6G095330
Csa2G370430	Csa3G017320	Csa7G447800	Csa6G095920
Csa2G370430	Csa3G015870	Csa7G447800	Csa6G103540
Csa2G370430	Csa3G011760	Csa7G447800	Csa1G264520
Csa2G370430	Csa3G011650	Csa7G447800	Csa1G267270
Csa2G370430	Csa3G008920	Csa7G447800	Csa1G268290
Csa2G370430	Csa3G008880	Csa7G447800	Csa4G639770
Csa2G370430	Csa3G006610	Csa7G447800	Csa4G639870
Csa2G370430	Csa3G005590	Csa7G447800	Csa4G639900
Csa2G370430	Csa3G002380	Csa7G447800	Csa4G641650
Csa2G370430	Csa3G001770	Csa7G447800	Csa4G641660

Csa2G370430	Csa6G014570	Csa7G447800	Csa4G641700
Csa2G370430	Csa6G365720	Csa7G447800	Csa4G641760
Csa2G370430	Csa6G365730	Csa7G447800	Csa4G642540
Csa2G370430	Csa6G366420	Csa7G447800	Csa4G644640
Csa2G370430	Csa6G366480	Csa7G447800	Csa4G645830
Csa2G370430	Csa6G376250	Csa7G447800	Csa4G646020
Csa2G370430	Csa6G382370	Csa7G447800	Csa4G646060
Csa2G370430	Csa4G179170	Csa7G447800	Csa4G499830
Csa2G370430	Csa4G179160	Csa7G447800	Csa1G629050
Csa2G370430	Csa1G696440	Csa7G447800	Csa1G629150
Csa2G370430	Csa1G699630	Csa7G447800	Csa1G629200
Csa2G370430	Csa4G190020	Csa7G447800	Csa1G638460
Csa2G370430	Csa3G813290	Csa7G447800	Csa1G654920
Csa2G370430	Csa3G815460	Csa7G447800	Csa1G660180
Csa2G370430	Csa3G816120	Csa7G447800	Csa4G571770
Csa2G370430	Csa3G816680	Csa7G447800	Csa4G563700
Csa2G370430	Csa3G820520	Csa7G447800	Csa4G561690
Csa2G370430	Csa3G824750	Csa7G447800	Csa4G552160
Csa2G370430	Csa3G826690	Csa7G447800	Csa4G551130
Csa2G370430	Csa3G827370	Csa7G447800	Csa4G420210
Csa2G370430	Csa3G829160	Csa7G447800	Csa4G418560
Csa2G370430	Csa3G835880	Csa7G447800	Csa7G446900
Csa2G370430	Csa3G838720	Csa7G447800	Csa7G446920
Csa2G370430	Csa3G848160	Csa7G447800	Csa7G446970
Csa2G370430	Csa3G848200	Csa7G447800	Csa7G447990
Csa2G370430	Csa3G848850	Csa7G447800	Csa7G448020
Csa2G370430	Csa3G860240	Csa7G447800	Csa7G448840
Csa2G370430	Csa3G865330	Csa7G447800	Csa7G451300
Csa2G370430	Csa3G872070	Csa7G447800	Csa7G451350
Csa2G370430	Csa3G872140	Csa7G447800	Csa7G451390
Csa2G370430	Csa6G087940	Csa7G447800	Csa6G081510
Csa2G370430	Csa6G087990	Csa7G447800	Csa6G080340
Csa2G370430	Csa6G091290	Csa7G447800	Csa6G080330
Csa2G370430	Csa6G091990	Csa7G447800	Csa6G080320
Csa2G370430	Csa1G268310	Csa7G447800	Csa6G078630
Csa2G370430	Csa1G269880	Csa7G447800	Csa6G078530
Csa2G370430	Csa4G639900	Csa7G447800	Csa6G078510
Csa2G370430	Csa4G639950	Csa7G447800	Csa6G076800
Csa2G370430	Csa4G641640	Csa7G447800	Csa6G076720
Csa2G370430	Csa4G641690	Csa7G447800	Csa6G075110
Csa2G370430	Csa4G641770	Csa7G447800	Csa6G074550
Csa2G370430	Csa4G642500	Csa7G447800	Csa6G067420
Csa2G370430	Csa4G644620	Csa7G447800	Csa6G057180
Csa2G370430	Csa4G644640	Csa7G447800	Csa6G056510
Csa2G370430	Csa4G645900	Csa7G447800	Csa6G056490
Csa2G370430	Csa4G646020	Csa7G447800	Csa6G046470
Csa2G370430	Csa1G630300	Csa7G447800	Csa6G046410
Csa2G370430	Csa1G642540	Csa7G447800	Csa6G046330

Csa2G370430	Csa4G554180	Csa7G447800	Csa6G046300
Csa2G370430	Csa4G551140	Csa7G447800	Csa6G030440
Csa2G370430	Csa4G425740	Csa7G447800	Csa6G022330
Csa2G370430	Csa7G447700	Csa7G447800	Csa6G022310
Csa2G370430	Csa7G447750	Csa7G447800	Csa6G017090
Csa2G370430	Csa7G448820	Csa7G447800	Csa6G017060
Csa2G370430	Csa7G448890	Csa7G447800	Csa6G016950
Csa2G370430	Csa7G451310	Csa7G447800	Csa2G292830
Csa2G370430	Csa7G451920	Csa7G447800	Csa2G292810
Csa2G370430	Csa6G077460	Csa7G447800	Csa2G286480
Csa2G370430	Csa6G074550	Csa7G447800	Csa2G285930
Csa2G370430	Csa6G053320	Csa7G447800	Csa2G277000
Csa2G370430	Csa6G042330	Csa7G447800	Csa2G277600
Csa2G370430	Csa6G041170	Csa7G447800	Csa2G277620
Csa2G370430	Csa6G040580	Csa7G447800	Csa5G201320
Csa2G370430	Csa6G032500	Csa7G447800	Csa5G199810
Csa2G370430	Csa2G285930	Csa7G447800	Csa4G371070
Csa2G370430	Csa2G279230	Csa7G447800	Csa5G291680
Csa2G370430	Csa7G014570	Csa7G447800	Csa5G118170
Csa2G370430	Csa7G012410	Csa7G447800	Csa5G128230
Csa2G370430	Csa7G012400	Csa7G447800	Csa5G128250
Csa2G370430	Csa7G012380	Csa7G447800	Csa4G164350
Csa2G370430	Csa4G371070	Csa7G447800	Csa3G303130
Csa2G370430	Csa5G114620	Csa7G447800	Csa3G305660
Csa2G370430	Csa7G010300	Csa7G447800	Csa3G751430
Csa2G370430	Csa7G009790	Csa7G447800	Csa3G748780
Csa2G370430	Csa7G007840	Csa7G447800	Csa3G746620
Csa2G370430	Csa2G169720	Csa7G447800	Csa3G745020
Csa2G370430	Csa6G151680	Csa7G447800	Csa6G301070
Csa2G370430	Csa6G151120	Csa7G447800	Csa6G301600
Csa2G370430	Csa6G149400	Csa7G447800	Csa6G302710
Csa2G370430	Csa6G149360	Csa7G447800	Csa6G303210
Csa2G370430	Csa6G147590	Csa7G447800	Csa7G010300
Csa2G370430	Csa6G147460	Csa7G447800	Csa7G009170
Csa2G370430	Csa2G200410	Csa7G447800	Csa7G009130
Csa2G370430	Csa2G200440	Csa7G447800	Csa7G007880
Csa2G370430	Csa1G050000	Csa7G447800	Csa2G165670
Csa2G370430	Csa1G050400	Csa7G447800	Csa2G169770
Csa2G370430	Csa1G050420	Csa7G447800	Csa2G171830
Csa2G370430	Csa2G348250	Csa7G447800	Csa2G190740
Csa2G370430	Csa2G348820	Csa7G447800	Csa2G193330
Csa2G370430	Csa2G348970	Csa7G447800	Csa2G193360
Csa2G370430	Csa2G349590	Csa7G447800	Csa2G193370
Csa2G370430	Csa2G349620	Csa7G447800	Csa2G193380
Csa2G370430	Csa2G350220	Csa7G447800	Csa6G153490
Csa2G370430	Csa2G350230	Csa7G447800	Csa6G147460
Csa2G370430	Csa2G350490	Csa7G447800	Csa6G146940
Csa2G370430	Csa2G351660	Csa7G447800	Csa2G200410

Csa2G370430	Csa2G354020	Csa7G447800	Csa2G211450
Csa2G370430	Csa2G354690	Csa7G447800	Csa2G213970
Csa2G370430	Csa2G354700	Csa7G447800	Csa2G215490
Csa2G370430	Csa2G354950	Csa7G447800	Csa2G222110
Csa2G370430	Csa2G357260	Csa7G447800	Csa7G368090
Csa2G370430	Csa1G586780	Csa7G447800	Csa7G373520
Csa2G370430	Csa1G586800	Csa7G447800	Csa7G374590
Csa2G370430	Csa4G099230	Csa7G447800	Csa7G378470
Csa2G370430	Csa4G107450	Csa7G447800	Csa7G378480
Csa2G370430	Csa4G109010	Csa7G447800	Csa7G378500
Csa2G370430	Csa4G111620	Csa7G447800	Csa7G378560
		Csa7G447800	Csa1G050220
		Csa7G447800	Csa1G050360
		Csa7G447800	Csa5G021310
		Csa7G447800	Csa2G346000
		Csa7G447800	Csa2G348840
		Csa7G447800	Csa2G348900
		Csa7G447800	Csa2G349630
		Csa7G447800	Csa2G350290
		Csa7G447800	Csa2G351020
		Csa7G447800	Csa2G351700
		Csa7G447800	Csa2G354780
		Csa7G447800	Csa2G354860
		Csa7G447800	Csa2G354920
		Csa7G447800	Csa2G356080
		Csa7G447800	Csa2G357270
		Csa7G447800	Csa4G109020
